# HIV latency and integration site placement in five cell-based models

**DOI:** 10.1186/1742-4690-10-90

**Published:** 2013-08-16

**Authors:** Scott Sherrill-Mix, Mary K Lewinski, Marylinda Famiglietti, Alberto Bosque, Nirav Malani, Karen E Ocwieja, Charles C Berry, David Looney, Liang Shan, Luis M Agosto, Matthew J Pace, Robert F Siliciano, Una O’Doherty, John Guatelli, Vicente Planelles, Frederic D Bushman

**Affiliations:** 1 Department of Microbiology, University of Pennsylvania School of Medicine, Philadelphia, PA, USA; 2 Department of Medicine, University of California at San Diego, La Jolla, CA, USA; 3 Division of Immunology, Transplantation and Infectious Diseases, San Raffaele Scientific Institute, Milano, Italy; 4 Department of Pathology, University of Utah, Salt Lake City, UT, USA; 5 Division of Biostatistics and Bioinformatics, Department of Family and Preventive Medicine, University of California at San Diego, La Jolla, CA, USA; 6 Department of Medicine, VA San Diego Healthcare System, University of California San Diego, La Jolla, California, USA; 7 Department of Medicine, Johns Hopkins University School of Medicine, Baltimore, MD, USA; 8 Department of Pathology and Laboratory Medicine, University of Pennsylvania School of Medicine, Philadelphia, Pennsylvania, USA; 9 Department of Microbial Pathogenesis, Yale University School of Medicine, New Haven, CT, USA

**Keywords:** HIV-1, Latency, Cure, Cell model, Integration sites, Meta-analysis, Central memory CD4 ^+^ T cells

## Abstract

**Background:**

HIV infection can be treated effectively with antiretroviral agents, but the persistence of a latent reservoir of integrated proviruses prevents eradication of HIV from infected individuals. The chromosomal environment of integrated proviruses has been proposed to influence HIV latency, but the determinants of transcriptional repression have not been fully clarified, and it is unclear whether the same molecular mechanisms drive latency in different cell culture models.

**Results:**

Here we compare data from five different *in vitro* models of latency based on primary human T cells or a T cell line. Cells were infected in vitro and separated into fractions containing proviruses that were either expressed or silent/inducible, and integration site populations sequenced from each. We compared the locations of 6,252 expressed proviruses to those of 6,184 silent/inducible proviruses with respect to 140 forms of genomic annotation, many analyzed over chromosomal intervals of multiple lengths. A regularized logistic regression model linking proviral expression status to genomic features revealed no predictors of latency that performed better than chance, though several genomic features were significantly associated with proviral expression in individual models. Proviruses in the same chromosomal region did tend to share the same expressed or silent/inducible status if they were from the same cell culture model, but not if they were from different models.

**Conclusions:**

The silent/inducible phenotype appears to be associated with chromosomal position, but the molecular basis is not fully clarified and may differ among *in vitro* models of latency.

## Background

Highly active antiretroviral therapy (HAART) can suppress HIV-1 replication in infected patients, but the ability of HIV to persist as an inducible reservoir of latent proviruses [1-3] obstructs eradication of the virus and functional cure [4]. These latent proviruses are long lived [5,6] and relatively invisible to the immune system [2,7]. The potential for even a single virus to restart infection despite successful antiviral therapy means that it may be necessary to eliminate all latent proviruses to eradicate HIV from an infected person.

After integration, a positive feedback loop of Tat transactivation appears to partition proviral gene activity into either of two stable states [8-10]—abundant Tat driving high proviral expression or little Tat leading to quiescent latency. Similar to the positional effect variegation observed in fruit fly chromosomal rearrangements [11,12], studies on cell clones with single integrations show that differing integration sites can have large differences in proviral expression [13-15]. These data suggest that integration site location, along with the cellular environment [15-18], influences the balance between latency and proviral expression.

Associations between latency and genomic features have also been reported in collections of integration sites from cell culture models although the consistency of these effects across model systems and their relationships to latency in patients remains uncertain. Lewinski et al. [19] reported that proviruses integrated in gene deserts, alphoid repeats and highly expressed genes are more likely to have low expression. Shan et al. [20] reported an association between latency and integration in the same transcriptional orientation as host genes. Pace et al. [21] found that silent and expressed provirus integration sites differed in the abundance and expression levels of nearby genes, GC content, CpG islands and alphoid repeats. In model systems with defined integration sites, Lenasi et al. [22] reported decreased and Han et al. [23] reported increased viral transcription when the provirus is downstream of a highly expressed host gene.

Cell-based models of latency are important for many aspects of HIV research, including screening small molecules that can reverse latency and potentially allow eradication [24,25]. Location-driven differences in expression are preserved even after DNA methyltransferase and histone deacetylase inhibitor treatments [13], which suggests that integration location has the potential to confound “shock and kill” anti-latency treatments [26,27]. A greater understanding of the effects of integration site location on latency could thus affect antiretroviral development.

To search for features of integration site associated with latency, we generated a set of inducible and expressed integration sites using a primary central memory CD4 ^+^ T cell model of latency [28,29], collected four previously reported integration site datasets and modeled the effects of genomic features near the integration site on the expression status of these proviruses. Although some genomic features associated with latency in individual models, no feature was consistently associated with proviral expression across all five cell culture models. However, closely neighboring proviruses within the same cellular model shared the same latency status much more often than expected by chance suggesting that chromosomal position of integration affects latency but that the mechanism remains unclear or differs between cell culture models. Thus these data help inform the design of experiments in HIV eradication research.

## Results

The combination of integration site data newly reported here (set named “Central Memory CD4 ^+^”) with previously published data (sets named “Jurkat”, “Bcl-2 transduced CD4 ^+^”, “Active CD4 ^+^ & Resting CD4 ^+^”) provides a collection of 12,436 integration sites (Table 1) where the expression status of the provirus—silent/inducible or expressed—is known. In three of the datasets, Jurkat, Central Memory CD4 ^+^ and Bcl-2 transduced CD4 ^+^, the proviruses were sorted based on inducibility. In the Resting CD4 ^+^ and Active CD4 ^+^ datasets, cells were sorted only based on proviral expression. Previous studies have shown that most silent proviruses in this model system are inducible [30].

**Table 1:** **HIV-1 integration datasets from*in vitro* models of latency**

**Title**	**Cell type**	**Virus**	**Time of harvest after infection**	**Sequencing**	**Generation of expressed vs. silent/inducible**	**Citation**	**Silent/inducible unique sites**	**Expressed unique sites**
Jurkat	Jurkat cells	HIV vector pEV731(LTR-Tat-IRES-GFP)	2 weeks	Sanger	TNF *α*, GFP expression	[19]	463inducible	643
Bcl-2 transduced CD4 ^+^	Primary CD4 ^+^T cells (Bcl-2transduced)	HIV NL4-3- *Δ*6-drEGFP(inactivated *gag*, *vif*,*vpr*, *vpu*, *nef* and *env*replaced by GFP)	3 days + 3-4weeks + 3 days	Sanger	Anti-CD3, anti-CD28 antibodies, GFP expression	[20]	446inducible	273
Active CD4 ^+^	Primary activeCD4 ^+^ T cells	HIV NL4-3	3 days	454	High vs. low Gag	[21]	1604silent	1274
Resting CD4 ^+^	Primary restingCD4 ^+^ T cells	HIV NL4-3	3 days	454	High vs. low Gag	[21]	1942silent	784
Central Memory CD4 ^+^	Primary centralmemory CD4 ^+^T cells	HIV NL4-3 *Δ*Nef GFP	2 days/9 days	IonTorrent	Anti-CD3, anti-CD28 antibodies, GFP expression	This paper	1729inducible	3278

### Global model

**Figure 1:**
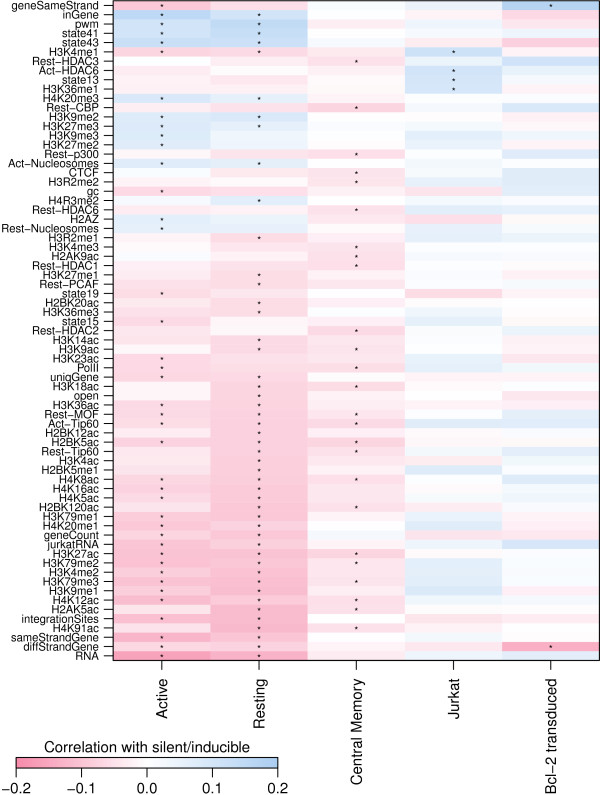
**Correlations of genomic features and latency.** Spearman rank correlation between proviral expression status and genomic features. Only genomic features with at least one correlation with latency with a false discovery rate *q*-value >0.01 (marked by asterisks) are shown.

If a genomic feature and latency are monotonically related then we should be able to detect this relationship using Spearman rank correlation. In addition if a feature has a consistent effect across models we should see a consistent pattern in the direction of correlation. A simple first look for correlation between genomic features (Table 2) and latency status yielded inconsistent results among the five samples with no variables having a significant Spearman rank correlation across all, or even four out of five, of the samples (Figure 1). This suggests that there is not a consistent simple monotonic relationship between the genomic variable and latency, or that any such correlations are modest and not detectable across all studies given the available statistical power. We return to some of the stronger trends below.

**Table 2:** Genomic data available for comparison to HIV integration sites

**Group**	**Type**	**Source**	**Number**	**Types**
T cell expression	RNA-Seq	Unpublished	1	RNA
Jurkat expression	RNA-Seq	Encode [31]	1	wgEncodeHudsonalphaRnaSeq
Integration sites	Locations	Unpublished	1	Sites
DNase sensitivity	DNA-Seq/peaks	Encode [31]	1	wgEncodeOpenChromDnase
Methylation	DNA-Seq	[32]	1	Methyl
CpG	Locations	UCSC [33]	1	cpgIslandExt
Sequence-based	Continuous	—	4	% GC, HIV PWM score, distance to centrosome, chromosomal position
Repeats	Locations	UCSC [33]	16	DNA, LINE, Low_complexity, LTR, Other, RC, RNA, rRNA, Satellite, scRNA, Simple_repeat, SINE, snRNA, srpRNA, tRNA, alphoid
Histone acetylation	ChIP-Seq/Peaks	[34]	18	H2AK5ac, H2AK9ac, H2BK120ac, H2BK12ac, H2BK20ac, H2BK5ac, H3K14ac, H3K18ac, H3K23ac, H3K27ac, H3K36ac, H3K4ac, H3K9ac, H4K12ac, H4K16ac, H4K5ac, H4K8ac, H4K91ac
Histone methylation and other proteins	ChIP-Seq/Peaks	[35]	23	CTCF, H2AZ, H2BK5me1, H3K27me1, H3K27me2, H3K27me3, H3K36me1, H3K36me3, H3K4me1, H3K4me2, H3K4me3, H3K79me1, H3K79me2, H3K79me3, H3K9me1, H3K9me2, H3K9me3, H3R2me1, H3R2me2, H4K20me1, H4K20me3, H4R3me2, PolII
Chromatin state	Binary	[59]	51	State_1_,state_2_,…,state_51_
HATs and HDACs	ChIP-Seq	[36]	11	Resting-HDAC1, Resting-HDAC2, Resting-HDAC3, Resting-HDAC6, Resting-p300, Resting-CBP, Resting-MOF, Resting-PCAF, Resting-Tip60, Active-HDAC6, Active-Tip60
Nucleosome	ChIP-Seq	[37]	2	Resting-Nucleosomes, Active-Nucleosomes
UCSC genes	Locations	[38]	4	In gene, in gene (same strand), gene count, distance to nearest gene, in exon, in intron

To investigate whether a combination of variables may affect latency, we fit a lasso-regularized logistic regression, as implemented in the R package glmnet [39], to predict latency using the genomic variables. The relationship between silent/inducible status and each genomic variable was allowed to vary between models by including the interaction of genomic features with dummy variables indicating cellular model. The *λ* smoothing parameter of the lasso regression was optimized by finding the *λ* with lowest classification error in 480-fold cross validation and finding the simplest model with misclassification error within one standard error.

**Figure 2:**
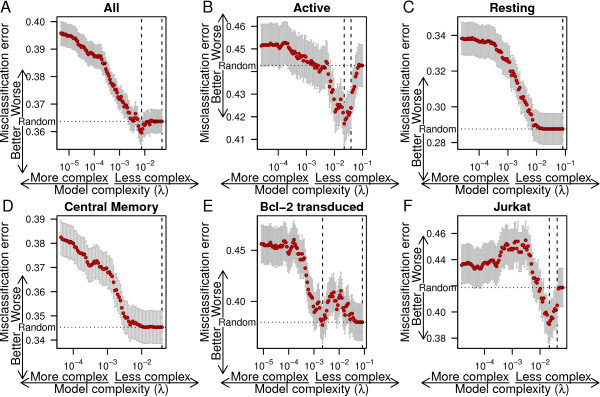
**Lasso regressions predicting latency.** Misclassification error from cross validation for lasso regressions of silent/inducible status on genomic features as a function of *λ*, the regularization coefficient for the lasso regression, for all cell culture models combined **(A)** and each individual cell culture model **(B-F)**. The number of variables included and size of coefficients in the model increases to the left. Whiskers show the standard error of mean misclassification error. Dashed vertical lines indicate the minimum misclassification error and the simplest model within one standard error. Dotted horizontal line indicates the misclassification error expected from random guessing.

The proportion of silent/inducible sites varied between the samples. To avoid the model overfitting on this source of variation, an indicator variable for each sample was included in the base model. The base model with no genomic variables was selected as the best model by cross validation (Figure 2A). This suggest that there is not a consistent linear relationship between an additive combination of genomic variables and latency across all models.

When each dataset was fit individually with leave-one-out cross validation, improvements in cross-validated misclassification error were only observed in the Active CD4 ^+^ (5.8% decrease in misclassification error, standard error: 2.1) and Jurkat (6.7% decrease in misclassification error, standard error: 3.5) samples (Figure 2B-F). There was no overlap in variables selected for the Active CD4 ^+^ and Jurkat samples.

Finding little global association between latency and genomic features, we investigated whether predictors of latency reported previously by single studies were consistently associated with latency across studies.

### Cellular transcription

Model systems with defined integration sites show upstream transcription can interfere with viral transcription [40] and that cellular transcription in the same orientation may interfere with viral transcription [22] or increase viral transcription [23] and in opposite orientations may decrease transcription [23]. In integration site studies, integration outside genes appears to increase latency [19] but high transcription of nearby host cell genes may cause increased latency [19,20]. In addition, Tat or other viral proteins may affect cellular transcription [41,42].

**Figure 3:**
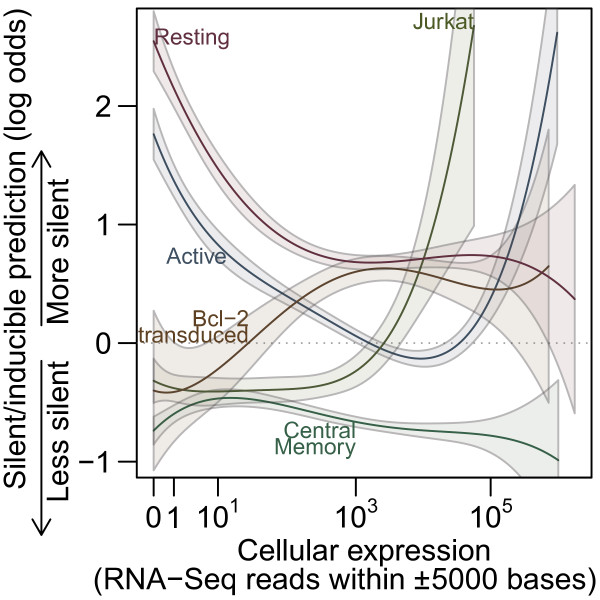
**Cellular expression and latency.** Predictions from a logistic regression of silent/inducible status on cellular RNA expression. High y-axis values are predicted to be silent/inducible. Dotted line shows where equal odds of silent/inducible and expressed are predicted. Solid lines show predictions from the regression for each sample and shaded regions indicate one standard error from the modeled predictions.

To look at transcription and latency, we ran a logistic regression of silent/inducible status on a quartic function of RNA expression, as determined by RNA-Seq reads within 5,000 bases in Jurkat cells for the Jurkat sample or CD4 ^+^ T cells for the remaining samples, interacted with indicator variables encoding cell culture model. There appears to be little agreement between samples (Figure 3). The Resting CD4 ^+^ and Active CD4 ^+^ datasets show an enrichment in silent proviruses in regions with low gene expression. The other three studies show the opposite or no relationship for low expression regions. The two samples showing increased silence in areas of low expression (Resting CD4 ^+^ and Active CD4 ^+^) are from a study that did not check whether inactive viruses could be activated. One possible explanation is that regions with low gene transcription may harbor proviruses that are not easily activated, though some other discrepancy between *in vitro* systems could also explain the difference. Both the Jurkat and Active CD4 ^+^ samples appear to increase in latency with increasing expression while the remaining three studies did not show a strong trend.

### Orientation bias

**Figure 4:**
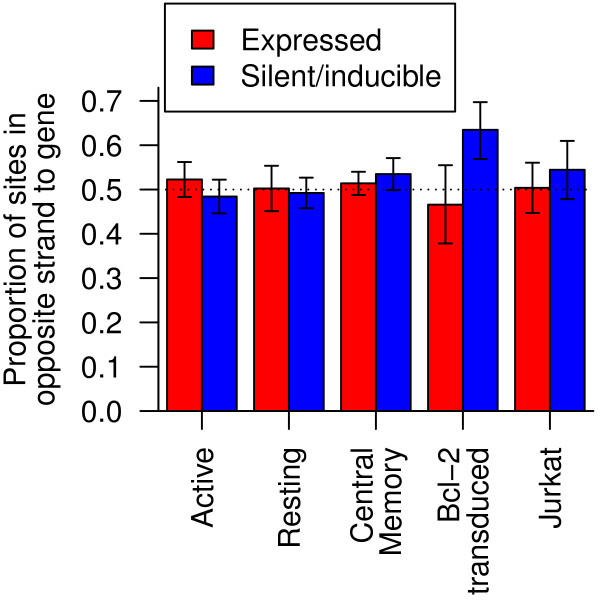
**Strand orientation and latency.** The proportion of provirus integrated in the opposite strand compared to cellular genes in silent/inducible (blue) and expressed (red) samples. Error bars show the 95% Clopper-Pearson binomial confidence interval. Dotted line shows the 0.5 proportion expected by chance.

Shan et al. [20] reported that inducible proviruses were oriented in the same strand as the host cell genes into which they had integrated more often than chance. This orientation bias was still reproduced after our reprocessing of the Bcl-2 transduced CD4 ^+^ sample from Shan et al. [20]. However, the proportion of provirus oriented in the same strand as host genes did not differ significantly from 50% in the other samples (Figure 4). Perhaps orientation bias and transcriptional interference are especially sensitive to parameters of the model system.

### Gene deserts

**Figure 5:**
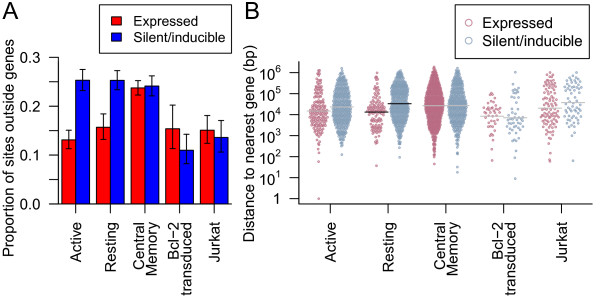
**Genes and latency.(A)** The proportion of provirus integrated outside genes in silent/inducible (blue) and expressed (red) samples. Error bars show the 95% Clopper-Pearson binomial confidence interval. **(B)** The nearest distance to any gene for integration sites (points) outside genes in the five samples. Points are spread in proportion to kernel density estimates. Horizontal lines indicate sample means where there was a significant difference in means between silent/inducible and expressed provirus (black) or no significant difference (grey).

Lewinski et al. [19] reported increased latency in gene deserts. In the collected data, integration outside known genes was associated with latency (Fisher’s exact test, *p*>^−6^). This seemed to largely be driven by the Active CD4 ^+^ and Resting CD4 ^+^ samples with significant association found individually in only those two samples (both *p*>^−8^) and no significant association observed in the other three samples (Figure 5A). Looking only at integration sites outside genes, silent sites in the Resting CD4 ^+^ sample had a mean distance to the nearest gene 2.5 times greater than that of expressed sites (95% CI: 2.2– 6.2×, *p*>^−6^, Welch two sample t-test on log transformed distance) (Figure 5B). The Active CD4 ^+^ sample had a small difference that did not survive Bonferroni correction.

Lewinski et al. [19] also reported decreased latency near CpG islands and reasoned this was tied to the increased latency in gene deserts. In the Resting CD4 ^+^ sample, silent sites were on average further from CpG islands than expressed sites (Bonferroni corrected Welch’s two sample T test, *p*=0.006), but there was no significant relationship between silent/inducible status and log distance to CpG island after Bonferroni correction if the integration site’s location inside or outside of a gene was accounted for first (analysis of deviance).

### Alphoid repeats

Alphoid repeats are repetitive DNA sequences found largely in the heterochromatin of centromeres [43]. Integration near heterochromatic alphoid repeats has been reported to associate with latency [14,19,21]. Looking only at uniquely mapping sites, there was no statistically significant association between latency and location inside an alphoid repeat in pooled or individual samples (Fisher’s exact test).

**Figure 6:**
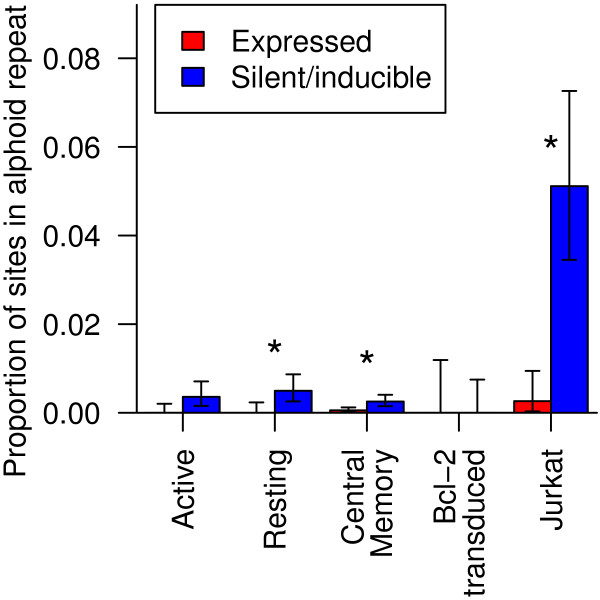
**Alphoid repeats and latency.** The proportion of integration sites with matches in alphoid repeats in silent/inducible (blue) and expressed (red) cells in five samples. Error bars show the 95% Clopper-Pearson binomial confidence interval. Asterisks indicate significant associations between integrations within an alphoid repeat and proviral expression status (Bonferroni corrected Fisher’s exact test *p*>0.05).

Since alphoid repeats are both problematic to assemble in genomes and difficult to map onto, we reasoned that some alphoid hits might be lost or miscounted in the filtering procedures of the standard workup. To counteract this, we treated each sequence read as an independent observation of a proviral integration and included sequence reads with more than one best scoring alignment. For multiply aligned reads, we considered the read to have been inside an alphoid repeat if any of its best scoring alignments fell within a repeat. We found 74 reads with potential alphoid mappings. Integration inside alphoid repeats was significantly associated with the expression status of a provirus in the Resting CD4 ^+^, Jurkat and Central Memory CD4 ^+^ datasets (Bonferroni corrected Fisher’s exact test, all *p*>0.05) and approached significance in the Active CD4 ^+^ dataset (*p*=0.053) (Figure 6). The Bcl-2 transduced CD4 ^+^ data did not contain any integration sites in alphoid repeats, probably due to 1) the relatively low number of integration sites in the dataset and 2) to the requirement for cleavage at two Pst1 restriction sites, which are not found in the consensus sequence of alphoid repeats [44]. Of the 1340 repeat types in the RepeatMasker database [44], only alphoid repeats achieved a significant association with proviral expression in more than two datasets.

### Acetylation

Histone marks or chromatin remodeling, especially involving the key “Nuc-1” histone near the transcription start site in the viral LTR, appear to affect viral expression [15,45,46]. Based on this effect, histone deacetylase inhibitors have been developed as potential HIV treatments and show some promise in disrupting latency [27]. In these genome-wide datasets, we do not have information on the state of individual LTR nucleosomes. However, repressive chromatin does seem to spread to nearby locations if not blocked by insulators [11,12] and the state of neighboring chromatin could affect proviral transcription independently of provirus-associated histones.

**Figure 7:**
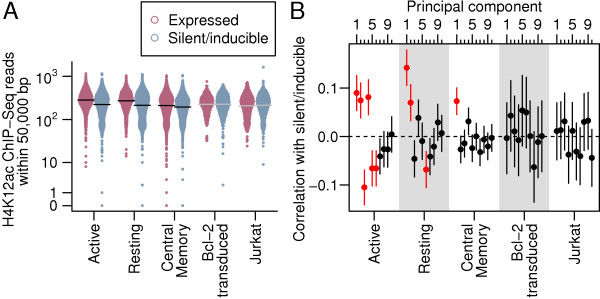
**Acetylation and latency.(A)** The number of ChIP-seq reads for H4K12ac, the histone mark with the lowest Fisher’s method *p*-value for correlation with latency, within 50,000 bases across the five samples. Integration sites (points) are spread in proportion to kernel density estimates. Horizontal lines indicate sample means where there was a significant difference (black) in means between silent/inducible and expressed provirus or no significant difference (grey). **(B)** The correlation (points) and its 95% confidence interval (vertical lines) between principal components of acetylation and silent/inducible status for each of the five samples. Red indicates correlations with a Bonferroni-corrected *p*-value >0.05.

We found that the number of ChIP-seq reads near an integration site from several histone acetylation marks (Figure 1) were associated with efficient expression in the Active CD4 ^+^, Resting CD4 ^+^ and Central Memory CD4 ^+^ samples. H4K12ac had the strongest association (Bonferroni corrected Fisher’s method combination of Spearman’s *ρ*, *p* > 10^−25^) with silence/latency (Figure 7A).

Although the appearance of several significantly associated acetylation marks might suggest acetylation exerts a considerable effect on the expression of a provirus, there are strong correlations among these marks, so their effects may not be independent. To account for the correlations between these variables, we performed a principal component analysis (PCA) to convert the correlated acetylation marks into a series of uncorrelated principal components that capture much of the variance within a few components. Here, the first principal component explained 59% of the variance and the first ten components 84%. Several of these principal components again displayed significant associations with latency in the Active CD4 ^+^, Resting CD4 ^+^ and Central Memory CD4 ^+^ samples but no significant correlations in the Bcl-2 transduced CD4 ^+^ or Jurkat samples (Figure 7B). A logistic regression of expression status on the first ten principal components and sample did not reduce misclassification error from a base model including only sample in 480-fold cross validation (base model misclassification error: 36.4%, PCA model: 36.5%). This suggests that acetylation of neighboring chromatin does not exert strong effects on latency in all samples.

### Clustering

**Figure 8:**
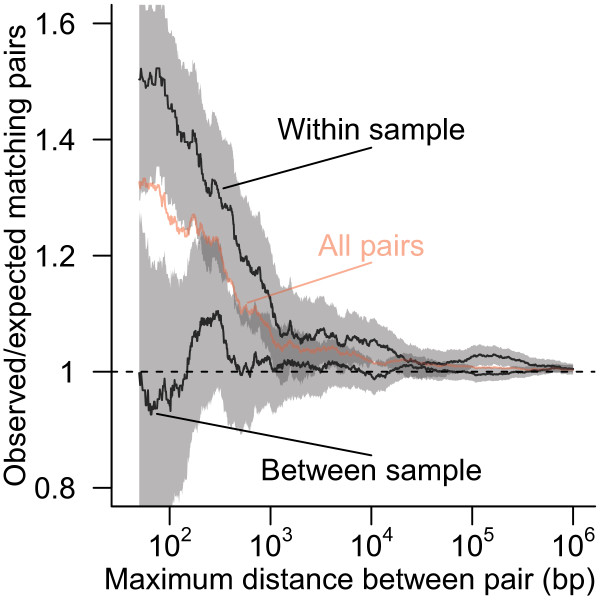
**Shared expression status between near neighbors.** The ratio of the number of pairs of proviruses with matching expression status to the number of matches expected by random pairings given the frequency of silent/inducible proviruses. All possible pairs of proviruses integrated within a given distance of each other on the same chromosome (red line) were separated into two sets; one with both proviruses from within the same cell culture model and one with proviruses paired between two different cell culture models (black lines). The shaded region shows the 95% Clopper-Pearson binomial confidence interval for within and between sample pairings. The dashed horizontal line shows the ratio of 1 expected if there is no association between the expression status of neighboring proviruses.

We reasoned that if there was a strong relationship between latency and chromosomal position, then integration sites that are near one another on the same chromosome should share the same expression status more often than expected by chance. To test this, we compared how often pairs of proviruses shared the same expression status in relation to the distance between the two sites (Figure 8). Pairs of sites with little distance between integration locations did share the same expression status more often than expected by chance (e.g. neighbors closer than 100 bp, Fisher exact test *p*=0.0002). Breaking out the data to separate between sample and within sample pairings showed that this matching was limited to neighbors within the same experimental model (Figure 8), emphasizing that chromosomal environment does appear to influence latency, but the factors involved differ among experimental models of latency.

## Discussion

Here we compared the latency status of HIV-1 proviruses in five model systems with the genomic features surrounding their integration sites. Surprisingly, no relationships between genomic features near the integration location and latency achieved significance in all models. Proviruses from the same cellular model integrated in nearby positions did share the same latency status much more often than predicted by chance, indicating the existence of local features influencing latency, but these were not consistent among models. This suggests that whatever features are affecting latency are highly local and model-specific, and that we may not have access to all relevant chromosomal features (e.g. [47-50]).

In addition to differences in experimental conditions, methodological issues have the potential to obscure patterns. Examples include multiply infected cells, inactivated viruses and inaccurate assessment of HIV gene activity—each of these are discussed below.

A latent provirus integrated into the same cell as an expressed provirus will be erroneously sorted as expressed, potentially confounding analysis. A low multiplicity of infection (MOI) will help to avoid this problem, but there is still the potential for a significant proportion of the cells studied to contain multiple integrations. This problem arises because although cells with multiple integrations form a small proportion of total cells, most of the total are cells lacking an integrated provirus and thus are excluded by experimental design. For example, assuming integrations are Poisson distributed with an MOI of 0.1 (1 integration per 10 cells), 90.5% of cells will not contain a provirus, 9% of cells will contain one proviral integration and 0.5% of cells will contain multiple integrations. The cells without an integration are not amplified by HIV-targeted PCR leaving only 9.5% of the total cells. Of these cells actually under study, 4.9% will contain multiple integrations. Thus the signal from expressed proviruses may be muted by the presence of latent proviruses in the expressed population.

The replication cycle of HIV is error prone, and a significant proportion of virions contain mutated genomes [51]. In studies that do not check for inducibility, mutant proviruses integrated in regions of the genome otherwise favorable to proviral expression can be sorted into the latent pool due to mutational inactivation. This problem of inactivated provirus is worse when latent provirus are rare and exacerbated further when looking at latency in the cells of HIV patients due to selective enrichment of inactivated proviruses incapable of spreading infection [2]. Here, the effects of mutation are minimized in the datasets that required inducible viral expression (Jurkat, Bcl-2 transduced CD4 ^+^, Central Memory CD4 ^+^) but may be a confounder in the two datasets that were sorted based on lack of viral expression only (Active CD4 ^+^, Resting CD4 ^+^).

Inaccurate staining or leaky markers may also result in misclassification of proviruses. False positives and false negatives will result in incorrectly sorted latent and expressed integrations. For example, if 5% of cells not containing Gag are labeled as Gag+ and there are an equal amount of latent and expressed integration sites, then 4.8% of integrations labeled expressed will actually be latent. If a category is rare, false staining has even greater potential to cause error. For example, if only 5% of sites are latent and a Gag stain has a false negative rate of 5%, then we would expect 48.7% of sites classified as latent to actually be mislabeled expressed integrations.

Attempts to induce latent proviruses in patients have so far focused on using histone deacetylase inhibitors, raising interest in associations with histone acetylation in these data. An important caveat in results from these genome-wide data is that histone modification near the integrated provirus may not be representative of modification within the provirus at the key “Nuc-1” nucleosome of the transcription start site [46], though local correlations in chromatin states are well established from studies of position effect variegation [11,12]. We found that some histone acetylation marks were significantly associated with viral expression in some but not all samples (Figures 1 and 7). This lack of association may be due to a lack of power in these studies, but the confidence intervals suggest that any correlations between acetylations and latency are unlikely to be strong. These weak correlations raise the possibility that there are populations of latent proviruses that are not associated with acetylation and may not be inducible by histone deacetylase inhibitors.

## Conclusions

This study highlights that the choice of model system can have a large effect on measurements of latency. Further studies are needed to determine which *in vitro* models best reflect latency *in vivo*. Different cell culture models may report genuinely different mechanisms of latency. While we did see some relationship between histone acetylation and latency, paralleling a recent clinical trial of SAHA [27], associations with histone acetylation did not explain a large fraction of the difference between latent and expressed proviruses in any of the five models. One possible explanation is that there may be multiple mechanisms that maintain proviruses in a latent state. To be successful, shock-and-kill treatments must induce and destroy all latent proviruses to eliminate HIV from an infected individual, raising the question of whether multiple simultaneous inducing treatments will be necessary.

## Availability of supporting data

Sequence reads from the Central Memory CD4 ^+^ sample reported here, the Resting CD4 ^+^ and Active CD4 ^+^ data reported by Pace et al. [21], the Bcl-2 transduced CD4 ^+^ data reported by Shan et al. [20] and reprocessed data originally reported by Lewinski et al. [19] are available at the Sequence Read Archive under accession number SRP028573.

## Methods

### Integration sites

Naive CD4 ^+^ T cells were purified by negative selection from peripheral blood mononuclear cells. The cells were activated with anti-CD3 and anti-CD28 (+TGF-beta, anti-IL-12, and anti-IL-4) to generate “non-polarized” cells (the *in vitro* equivalent of central memory T cells). Five days after isolation, cells were infected with an NL4-3-based virus with GFP in place of Nef and the LAI envelope (X4) provided in trans at a concentration of 500 ng of p24 as measured by ELISA per million cells. Based on previous experience with this model, this amount of p24 should produce an MOI of approximately 0.15. Cells were cultured in the presence of IL-2. Two days post-infection, cells were sorted for GFP+; this active population expresses GFP even when treated with flavopiridol, although for this study they were not treated. The inducible population was the set of GFP negative cells from the initial sort that, 9 days post-infection, were activated with anti-CD3 and anti-CD28 and sorted for GFP production.

Genomic DNA from the inducible and expressed populations was digested with MseI, ligated to an adapter, and amplified by ligation-mediated PCR essentially as in Wu et al. [52] and Mitchell et al. [53] except that the nested PCR primers included sequence for the Ion Torrent P1 adapter and adapter A sequence with a 5 base barcode sequence specific to the inducible or expressed conditions. Amplicons were sequenced using an Ion Torrent Personal Genome Machine (PGM) according to manufacturer’s instructions using an Ion 316 chip and the Ion PGM 200 Sequencing kit (Life Technologies). The sequence reads were sorted into samples by barcode. All reads were required to match the expected 5^′^ sequence with a Levenshtein edit distance less than 3 from the expected barcode, 5^′^ primer and HIV long terminal repeat (LTR). The 5^′^ primer and HIV sequence, along with the 3^′^ primer if present, were trimmed from the read. Sequences with less than 24 bases remaining or containing any eight base window with an average quality less than 15 were discarded. Duplicate reads and reads forming an exact substring of a longer read were removed.

### Previously published data

We collected integration sites from three previously reported studies (Table 1), for a total of four expressed versus silent/inducible pairs of samples. These studies used primary CD4 ^+^ T cells or Jurkat cells infected with HIV or HIV-derived constructs as cell culture models of latency. Flow cytometry allowed cells expressing viral encoded proteins to be sorted from non-expressing cells. In two of the studies, these non-expressing populations were stimulated to ensure that the provirus could be aroused from latency. Specific differences in protocol between the study sets are summarized below.

#### Jurkat

Lewinski et al. [19] infected Jurkat cells with a VSV-G pseudotyped, GFP-expressing pEV731 HIV construct (LTR-Tat-IRES-GFP) [13] at an MOI of 0.1. The cells were sorted into GFP+ and GFP- two to four days after infection. GFP+ cells were sorted again two weeks after infection and cells that were again GFP+ were collected for integration site sequencing. GFP- cells were sorted for GFP negativity twice more then stimulated with TNFalpha. Cells that were GFP+ after stimulation were collected for integration site sequencing. DNA was digested with MseI or a combination of NheI, SpeI and XbaI, ligated to adapters for nested PCR, amplified and sequenced by Sanger capillary electrophoresis.

#### Bcl-2 transduced CD4 ^+^

Shan et al. [20] transduced CD4 ^+^ T cells with Bcl-2, costimulated with bound anti-CD3 and soluble anti-CD28 antibodies, interleukin-2 and T cell growth factor and then infected with X4-pseudotyped GFP-expressing NL4-3- *Δ*6-drEGFP construct [54] at an MOI of less than 0.1. DNA was extracted, digested with PstI and circularized [55]. HIV-human junctions were amplified by reverse PCR and sequenced using Sanger capillary electrophoresis.

#### Active CD4 ^+^ & Resting CD4 ^+^

Pace et al. [21] spinoculated CD4 ^+^ T cells with HIV NL4-3 at an MOI of 0.1. After 96 hours, the cells were stained for intracellular Gag CD25, CD69 and HLA-DR and sorted into four subpopulations based on activation state and Gag expression; activated Gag-, activated Gag+, resting Gag- and resting Gag+. The ability of the viruses to reactivate was not tested although previous studies have shown that the majority are likely inducible [30]. Genomic DNA was extracted and digested with restriction enzymes MseI and Tsp509 and ligated to adapters. Proviral LTR-host genome junctions were sequenced by 454 pyrosequencing after nested PCR.

### Alignment

All datasets were processed using the hiReadsProcessor R package [56]. Adaptor trimmed reads were aligned to UCSC freeze hg19 using BLAT [57]. Genomic alignments were scored and required to start within the first three bases of a read with 98% identity. Alignments for a given read with a BLAT score less than the maximum score for that read were discarded. Reads giving rise to multiple best scoring genomic alignments were excluded, while reads with a single best hit were dereplicated and converged if within 5 bp of each other. The Bcl-2 transduced CD4 ^+^ sample was sequenced from U3 in the 5^′^ HIV LTR while the other samples were sequenced from U5 in the 3^′^ LTR. To account for the 5 base duplication of host DNA caused by HIV integration, the chromosomal coordinates of the Bcl-2 transduced CD4 ^+^ sample were adjusted by ±4 bases.

To allow for alignment difficulties in the analysis of genomic repeats, reads with multiple best scoring alignments, along with the single best hit reads used above, were included in the repeat analyses. If any best scoring alignment for a read fell within a repeat, then that read was considered to map to that repeat.

### Genomic features

A total of 140 whole genome features for CD4 ^+^ T-cells were gathered from data sources indicated in Table 2. For features encoded as peaks or hotspots, the log of the distance of each integration site to the nearest border was used for modeling. Integration sites from HIV 89.6 infection in primary CD4 ^+^ T cells (unpublished data) were used to count nearby integrations and determine a ±20 bp position weight matrix for integration targets. Illumina RNA-Seq from active CD4 ^+^ cells (unpublished data) was used to estimate raw cellular expression and fragments per kilobase of transcript per million mapped reads for genes as calculated by Cufflinks [58]. For sequence-based data like RNA-Seq and ChIP-Seq, the number of reads aligned within a ± 50, 500, 5,000 50,000 and 500,000 bp windows of each integration site were counted and log transformed. In addition, chromatin state classifications derived from a hidden Markov model based on histone marks and a few binding factors [59] were included as binary variables. All data from previous genomic freezes were converted to hg19 using liftover [60].

### Analysis

All statistical analysis was performed in R 2.15.2 [61]. The analyses are described in a reproducible report (Additional file 1). The annotated integration site data necessary to perform the analyses (Additional files 2 and 3) and the compilable code (Additional file 4) to generate this reproducible report are provided as supplemental information. The new Central Memory CD4 ^+^ data set was analyzed as in Berry et al. [62] (Additional file 5). The integration patterns appeared similar to previously reported HIV integration site datasets [63].

## Competing interests

The authors declare that they have no competing interests.

## Authors’ contributions

SS-M led the computational analysis, with assistance from CCB and NM. MKL, DL and JG analyzed integration sites using IonTorrent sequencing. MF, AB and VP prepared DNA from latent and activated T cells using the Central Memory CD4 ^+^ model. LS, RFS, MJP, LMA and UO’D contributed data and suggestions. SS-M, KEO and FDB planned the overall study, and SS-M and FDB wrote the paper. All authors read and approved the final manuscript.

## Supplementary Material

Additional file 1 **Summary of statistical analyses.** A pdf file showing the R code used for statistical analysis. Click here for file

Additional file 2 **Integration locations and surrounding genomic features.** A gzipped csv file containing all uniquely mapped integration sites and measures of the genomic features surrounding the integration sites. Click here for file

Additional file 3 **Integration locations and repeats.** A gzipped csv file containing integration sites with single and multiple genomic alignments and whether any of those alignments fell within genomic repeats. Click here for file

Additional file 4 **Compilable summary of statistical analyses.** A Sweave Rnw file that can be compiled with R, LaTeX and the data from Additional files 2 and 3 to generate Additional file 1. Click here for file

Additional file 5 **Genomic feature analysis of Central Memory CD4**^**+**^** and Bcl-2 transduced CD4**^**+**^** data.** A pdf file reporting the association of genomic features with integrations in the Central Memory CD4 ^+^ and Bcl-2 transduced CD4 ^+^ datasets following the methods of Berry et al. [62]. Click here for file
