# Supplementary material for: HIV latency and integration site placement in five cell-based models
Source: Retrovirology. 2013 Aug 16;10:90. doi: 10.1186/1742-4690-10-90 (PMC3765678; doi:10.1186/1742-4690-10-90)
Supplement: Additional file 1 — Summary of statistical analyses. A pdf file showing the R code used for statistical analysis. [file 1742-4690-10-90-S1.pdf]

1 Supplementary data

Additional File 2 is a gzipped csv file that includes a row for each uniquely mapped provirus and its surrounding genomic annotations. The csv file should have 12436 rows (excluding header) with 6252 expressed and 6184 latent proviruses.

```
> integrationData<-read.csv('AdditionalFile2.csv.gz',stringsAsFactors=FALSE)
> nrow(integrationData)
```

```
[1] 12436
```

```
> table(integrationData$isLatent)
```

```
FALSE  TRUE
 6252  6184
```

2 Lasso regression

The lasso regressions take a while to run so I've turned down the number of cross validations here (set `eval=FALSE` below to completely skip this step). Leave one out and 480-fold cross validation were used in the paper but processing may take a few days without parallel processing. Lasso regression requires the R glmnet package.

```
> notFitColumns<-c('id', 'chr', 'pos', 'strand', 'sample', 'isLatent')
> samples<-unique(as.character(integrationData$sample))
> sampleMatrix<-do.call(cbind, lapply(samples, function(x)integrationData$sample==x))
> colnames(sampleMatrix)<-gsub(' ', '_', samples)
> interact<-function(predMatrix, columns, addNames=NULL){
+         out<-do.call(cbind, lapply(1:ncol(columns), function(x)predMatrix*columns[, x]))
+         if(!is.null(addNames)){
+                 if(length(addNames)!=ncol(columns)){
+             stop(simpleError('Names not same length as columns'))
+         }
+                 colnames(out)<-sprintf("%s_%s", rep(addNames,
+                 each=ncol(predMatrix)), rep(colnames(predMatrix), length(addNames)))
+         }
+         return(out)
+ }
> fitData<-as.matrix(integrationData[, !colnames(integrationData) %in% notFitColumns])
> fitData2<-as.matrix(cbind(interact(fitData, sampleMatrix,
+             colnames(sampleMatrix)), fitData, sampleMatrix))
```

```
> library(glmnet)
> penalties<-rep(1, ncol(fitData2))
> penalties[ncol(fitData2)-(ncol(sampleMatrix):1)+1]<-0
> lassoFit<-cv.glmnet(fitData2, integrationData$isLatent, family='binomial',
+     type.measure='class', nfolds=3, penalty.factor=penalties)
> seperateFits<-lapply(samples, function(x)cv.glmnet(fitData[integrationData$sample==x, ],
+         integrationData$isLatent[integrationData$sample==x], family='binomial',
+         type.measure='class', nfolds=3))
> names(seperateFits)<-samples
```

```
>         if(exists('lassoFit')){
+                 par(mfrow=c(2,3))
+                 plot(lassoFit,main='All samples')
+                 dummy<-sapply(names(seperateFits),
+                         function(x)plot(seperateFits[[x]],main=x)
+                 )
```

```
+         }else{
+                 plot(1,1,main='Lasso regression not run.')
+         }
```

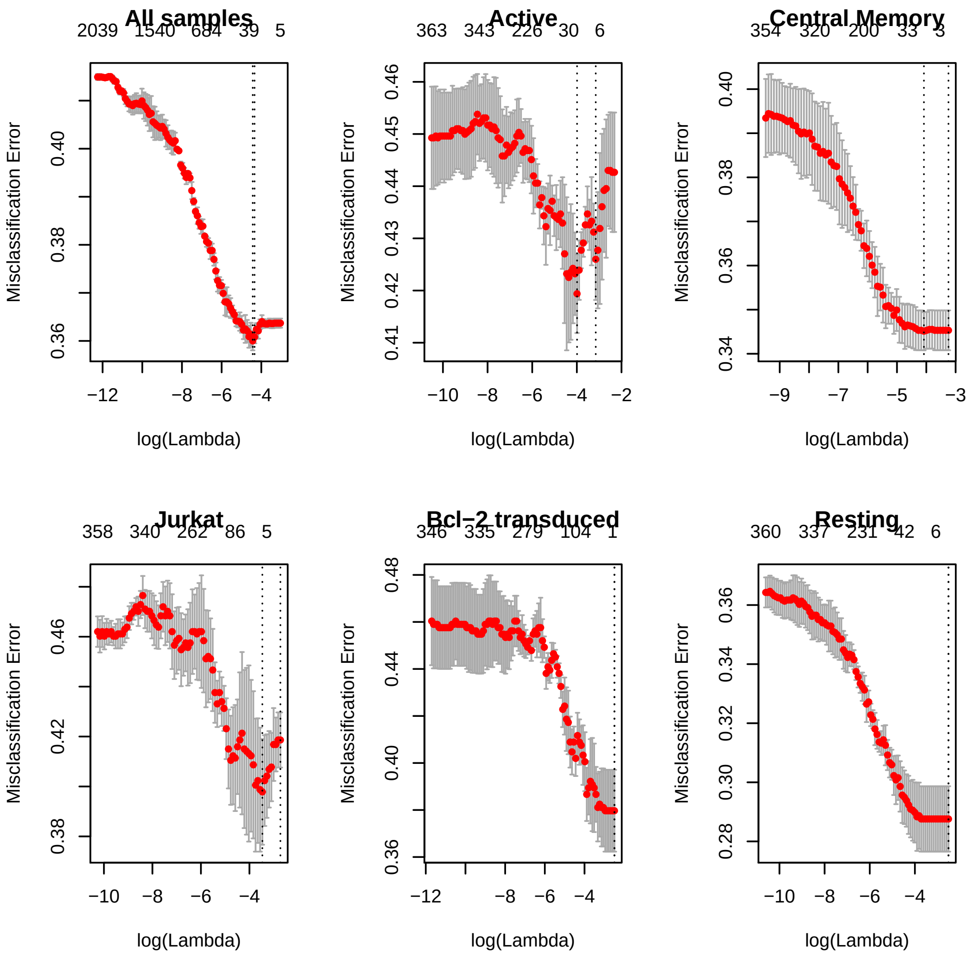

3 Correlation

We looked for correlation between the genomic variables and expression status of the proviruses.

```
> corMat<-apply(fitData, 2, function(x)sapply(samples, function(y){
+         selector<-integrationData$sample==y
+         if(sd(x[selector])==0)return(0)
+         isLatent<-integrationData[selector, 'isLatent']
+         cor(as.numeric(isLatent), x[selector], method='spearman')
+ }))
> quantile(corMat, seq(0, 1, .1))

          0%          10%          20%          30%          40%          50% 
-0.185223020 -0.081555830 -0.048938130 -0.030895834 -0.018053321 -0.005613895 
         60%          70%          80%          90%         100% 
 0.003580982  0.017822483  0.036694554  0.062003356  0.170642314 
```

If we looked for genomic variables consistently correlated or anti-correlated with proviral expression status with an FDR q-value less than 0.01, no variable was significantly correlated in more than 3 samples.

```
> pMat<-apply(fitData, 2, function(x)sapply(samples, function(y){
+         selector<-integrationData$sample==y
+         if(sd(x[selector])==0)return(NA)
+         isLatent<-integrationData[selector, 'isLatent']
+         cor.test(as.numeric(isLatent), x[selector],
+             method='spearman', exact=FALSE)$p.value
+ }))
> adjustPMat<-pMat
> adjustPMat[, ]<-p.adjust(pMat, 'fdr')
> downPMat<-upPMat<-adjustPMat
> downPMat[corMat>0]<-1
> upPMat[corMat<0]<-1
> table(apply(upPMat<.01&!is.na(upPMat), 2, sum))

  0   1   2   3
298  27  38  10

> table(apply(downPMat<.01&!is.na(downPMat), 2, sum))

  0   1   2   3
216  36  63  58
```

4 RNA expression

We fit a logistic regression to a polynomial of log RNA-Seq reads within 5000 bases from Jurkat cells for the Jurkat sample and T cells for the rest.

```
> rna<-ifelse(integrationData$sample=='Jurkat',
+     integrationData$log_jurkatRNA, integrationData$rna_5000)
> rna2<-rna^2
> rna3<-rna^3                          #
> rna4<-rna^4
> glmData<-data.frame(isLatent=integrationData$isLatent, sample=integrationData$sample,
+     rna, rna2, rna3, rna4)
> glmMod<-glm(isLatent~sample*rna+sample*rna2+sample*rna3+sample*rna4,
+     data=glmData, family='binomial')
> summary(glmMod)

Call:
glm(formula = isLatent ~ sample * rna + sample * rna2 + sample *
    rna3 + sample * rna4, family = "binomial", data = glmData)

Deviance Residuals:
    Min       1Q   Median       3Q      Max
-2.2899  -0.9864  -0.8676   1.0960   1.6007

Coefficients:
                              Estimate Std. Error z value Pr(>|z|)
(Intercept)                  1.7623655  0.2138859   8.240  < 2e-16 ***
sampleBcl-2 transduced      -2.1625912  0.7061524  -3.062  0.00219 **
sampleCentral Memory        -2.5010063  0.2437685 -10.260  < 2e-16 ***
sampleJurkat                -2.0800202  0.2836871  -7.332 2.27e-13 ***
sampleResting                0.7840481  0.3312247   2.367  0.01793 *
rna                         -0.6567268  0.2344422  -2.801  0.00509 **
rna2                         0.1387703  0.0770589   1.801  0.07173 .
rna3                        -0.0167219  0.0094076  -1.777  0.07549 .
rna4                         0.0007572  0.0003845   1.969  0.04891 *
```

```
sampleBcl-2 transduced:rna   0.5750186  0.6366537   0.903  0.36643
sampleCentral Memory:rna     0.9067758  0.2750955   3.296  0.00098 ***
sampleJurkat:rna             0.5294036  0.3867163   1.369  0.17101
sampleResting:rna            0.0366276  0.3436248   0.107  0.91511
sampleBcl-2 transduced:rna2 -0.0369353  0.1878816  -0.197  0.84415
sampleCentral Memory:rna2   -0.2106715  0.0915492  -2.301  0.02138 *
sampleJurkat:rna2           -0.0766215  0.1641153  -0.467  0.64059
sampleResting:rna2          -0.0760450  0.1086998  -0.700  0.48419
sampleBcl-2 transduced:rna3  0.0032503  0.0213743   0.152  0.87913
sampleCentral Memory:rna3    0.0237064  0.0112661   2.104  0.03536 *
sampleJurkat:rna3            0.0042183  0.0263910   0.160  0.87301
sampleResting:rna3           0.0153132  0.0128711   1.190  0.23415
sampleBcl-2 transduced:rna4 -0.0002532  0.0008267  -0.306  0.75939
sampleCentral Memory:rna4   -0.0009877  0.0004627  -2.135  0.03280 *
sampleJurkat:rna4            0.0001725  0.0014215   0.121  0.90339
sampleResting:rna4          -0.0008049  0.0005119  -1.572  0.11585
---
Signif. codes:  0 '***' 0.001 '**' 0.01 '*' 0.05 '.' 0.1 ' ' 1

(Dispersion parameter for binomial family taken to be 1)

    Null deviance: 17240  on 12435  degrees of freedom
Residual deviance: 15874  on 12411  degrees of freedom
AIC: 15924

Number of Fisher Scoring iterations: 4
```

5 Strand orientation

We used a Fisher's exact test to check if silent/inducible proviruses were enriched when integrated in the same strand orientation as cellular genes.

```
> selector<-integrationData$inGene==1
> strandTable<-with(integrationData[selector, ],
+     table(ifelse(isLatent, 'Silent/Inducible', 'Active'),
+     ifelse(inGeneSameStrand==1, 'Same', 'Diff'), sample))
> apply(strandTable, 3, fisher.test)

$Active

        Fisher's Exact Test for Count Data

data:  array(newX[, i], d.call, dn.call)
p-value = 0.06061
alternative hypothesis: true odds ratio is not equal to 1
95 percent confidence interval:
 0.7219466 1.0081995
sample estimates:
odds ratio
 0.8532127



$`Bcl-2 transduced`

        Fisher's Exact Test for Count Data
```

```
data:  array(newX[, i], d.call, dn.call)
p-value = 2.177e-05
alternative hypothesis: true odds ratio is not equal to 1
95 percent confidence interval:
 1.446896 2.872562
sample estimates:
odds ratio
  2.036148


$`Central Memory`

        Fisher's Exact Test for Count Data

data:  array(newX[, i], d.call, dn.call)
p-value = 0.2907
alternative hypothesis: true odds ratio is not equal to 1
95 percent confidence interval:
 0.9386167 1.2320238
sample estimates:
odds ratio
   1.07529


$Jurkat

        Fisher's Exact Test for Count Data

data:  array(newX[, i], d.call, dn.call)
p-value = 0.1674
alternative hypothesis: true odds ratio is not equal to 1
95 percent confidence interval:
 0.9207548 1.5699893
sample estimates:
odds ratio
  1.202007


$Resting

        Fisher's Exact Test for Count Data

data:  array(newX[, i], d.call, dn.call)
p-value = 0.5732
alternative hypothesis: true odds ratio is not equal to 1
95 percent confidence interval:
 0.7825231 1.1405158
sample estimates:
odds ratio
 0.9447415
```

6 Acetylation

To reduce correlation between acetylation marks, we generated the first ten principal components of the acetylation data and ran a logistic regression against them. We compared the cross validated performance of this regression with a base model only including which dataset the integration site came from. The

cross-validation here has been reduced for efficiency but 480-fold cross-validation was used in the paper.

```
> acetyl<-integrationData[, !grepl('logDist', colnames(integrationData)) &
+         grepl('ac', colnames(integrationData))]
> acetylPCA<-princomp(acetyl)
> cumsum(acetylPCA$sdev[1:10]^2/sum(acetylPCA$sdev^2))

   Comp.1    Comp.2    Comp.3    Comp.4    Comp.5    Comp.6    Comp.7    Comp.8
0.5947268 0.6786611 0.7267433 0.7610502 0.7833616 0.7964470 0.8093295 0.8215027
   Comp.9   Comp.10
0.8299358 0.8372584

> cv.glm<-function(model, K=nrow(thisData), subsets=NULL){
+     modelCall<-model$call
+     thisData<-eval(modelCall$data)
+     n<-nrow(thisData)
+     if(is.null(subsets))subsets<-split(1:n, sample(rep(1:K, length.out=n)))
+     preds<-lapply(subsets, function(outGroup){
+         subsetData<-thisData[-outGroup, , drop=FALSE]
+         predData<-thisData[outGroup, , drop=FALSE]
+         thisModel<-modelCall
+         thisModel$data<-subsetData
+         return(predict(eval(thisModel), predData))
+     })
+     pred<-unlist(preds)[order(unlist(subsets))]
+     subsetId<-rep(1:K, sapply(subsets, length))[order(unlist(subsets))]
+     return(data.frame(pred, subsetId))
+ }
> inData<-data.frame('isLatent'=integrationData$isLatent,
+         'sample'=as.factor(integrationData$sample), acetylPCA$score[, 1:10])
> modelPreds<-cv.glm(glm(isLatent~sample+Comp.1+Comp.2+Comp.3+Comp.4+Comp.5+
+                 Comp.6+Comp.7+Comp.8+Comp.9+Comp.10, family='binomial', data=inData), K=5)
> basePreds<-cv.glm(glm(isLatent~sample, family='binomial', data=inData),
+         subsets=split(1:nrow(inData), modelPreds$subsetId), K=5)
> modelCorrect<-sum((modelPreds$pred>0)==integrationData$isLatent)
> baseCorrect<-sum((basePreds$pred>0)==integrationData$isLatent)
> prop.test(c(baseCorrect, modelCorrect), rep(nrow(integrationData), 2))

        2-sample test for equality of proportions with continuity correction

data:  c(baseCorrect, modelCorrect) out of rep(nrow(integrationData), 2)
X-squared = 0.0627, df = 1, p-value = 0.8023
alternative hypothesis: two.sided
95 percent confidence interval:
 -0.01043491  0.01365137
sample estimates:
   prop 1    prop 2
0.6362978 0.6346896
```

7 Gene deserts

We used Fisher's exact test to look for an association between integration outside a gene and proviral expression status.

```
> geneTable<-table(integrationData$isLatent,
+     integrationData$inGene, integrationData$sample)
> apply(geneTable, 3, fisher.test)
```

```
$Active

        Fisher's Exact Test for Count Data

data:  array(newX[, i], d.call, dn.call)
p-value < 2.2e-16
alternative hypothesis: true odds ratio is not equal to 1
95 percent confidence interval:
 0.3629548 0.5446204
sample estimates:
odds ratio
 0.4452621


$`Bcl-2 transduced`

        Fisher's Exact Test for Count Data

data:  array(newX[, i], d.call, dn.call)
p-value = 0.1052
alternative hypothesis: true odds ratio is not equal to 1
95 percent confidence interval:
 0.9203418 2.3478599
sample estimates:
odds ratio
  1.472224


$`Central Memory`

        Fisher's Exact Test for Count Data

data:  array(newX[, i], d.call, dn.call)
p-value = 0.7803
alternative hypothesis: true odds ratio is not equal to 1
95 percent confidence interval:
 0.8525329 1.1253952
sample estimates:
odds ratio
 0.9791165


$Jurkat

        Fisher's Exact Test for Count Data

data:  array(newX[, i], d.call, dn.call)
p-value = 0.5443
alternative hypothesis: true odds ratio is not equal to 1
95 percent confidence interval:
 0.7909269 1.6167285
sample estimates:
odds ratio
  1.127836
```

```
$Resting

        Fisher's Exact Test for Count Data

data:  array(newX[, i], d.call, dn.call)
p-value = 3.071e-08
alternative hypothesis: true odds ratio is not equal to 1
95 percent confidence interval:
 0.4384828 0.6864112
sample estimates:
odds ratio
 0.5500205
```

We used a two-sample t-test to investigate whether there was a significant difference in distance to the nearest gene between expressed and silent/inducible proviruses integrated outside genes.

```
> geneDistData<-integrationData[!integrationData$inGene,
+     c('isLatent', 'logDist_nearest', 'sample')]
> by(geneDistData, geneDistData$sample, function(x)t.test(logDist_nearest~isLatent,
+         data=x))

geneDistData$sample: Active

        Welch Two Sample t-test

data:  logDist_nearest by isLatent
t = -2.4539, df = 287.731, p-value = 0.01472
alternative hypothesis: true difference in means is not equal to 0
95 percent confidence interval:
 -0.80738340 -0.08867607
sample estimates:
mean in group FALSE  mean in group TRUE
           9.608737           10.056767

------------------------------------------------------------
geneDistData$sample: Bcl-2 transduced

        Welch Two Sample t-test

data:  logDist_nearest by isLatent
t = 0.4098, df = 86.2, p-value = 0.683
alternative hypothesis: true difference in means is not equal to 0
95 percent confidence interval:
 -0.6309351  0.9586004
sample estimates:
mean in group FALSE  mean in group TRUE
           9.036872            8.873039

------------------------------------------------------------
geneDistData$sample: Central Memory

        Welch Two Sample t-test

data:  logDist_nearest by isLatent
t = -0.0719, df = 861.606, p-value = 0.9427
alternative hypothesis: true difference in means is not equal to 0
95 percent confidence interval:
```

```
 -0.2371374  0.2203819
sample estimates:
mean in group FALSE  mean in group TRUE
           10.19225            10.20063

------------------------------------------------------------
geneDistData$sample: Jurkat

        Welch Two Sample t-test

data:  logDist_nearest by isLatent
t = -1.8217, df = 139.564, p-value = 0.07064
alternative hypothesis: true difference in means is not equal to 0
95 percent confidence interval:
 -1.26342086  0.05167979
sample estimates:
mean in group FALSE  mean in group TRUE
           9.925782           10.531652

------------------------------------------------------------
geneDistData$sample: Resting

        Welch Two Sample t-test

data:  logDist_nearest by isLatent
t = -5.1275, df = 193.491, p-value = 7.096e-07
alternative hypothesis: true difference in means is not equal to 0
95 percent confidence interval:
 -1.2687917 -0.5638568
sample estimates:
mean in group FALSE  mean in group TRUE
           9.489931           10.406255
```

To check for a relationship between silent/inducible status and distance to CpG islands, we used a two sample t-test on the logged distance and saw a significant difference between silent/inducible and expressed proviruses (before accounting for a correlation between being near CpG islands and in genes)

```
> t.test(integrationData$logDist_cpg~integrationData$isLatent)

        Welch Two Sample t-test

data:  integrationData$logDist_cpg by integrationData$isLatent
t = -2.0233, df = 12381.27, p-value = 0.04306
alternative hypothesis: true difference in means is not equal to 0
95 percent confidence interval:
 -0.105657514 -0.001675563
sample estimates:
mean in group FALSE  mean in group TRUE
           10.16362            10.21728

> sapply(unique(integrationData$sample),
+         function(x)with(integrationData[integrationData$sample==x,],
+                p.adjust(
+                        t.test(logDist_cpg~isLatent)$p.value
+                ,method='bonferroni',n=5))
+ )
          Active   Central Memory           Jurkat Bcl-2 transduced
     0.512040457      1.000000000      1.000000000      1.000000000
```

```
        Resting
    0.005866539
```

Many CpG islands are found near genes. To account for this relationship, we used an ANOVA test including whether the integration site was inside a gene prior to including CpG islands. After including integration inside genes, CpG islands were not significantly associated with silent/inducible status of the proviruses with all samples grouped or individually after Bonferonni correction for multiple comparisons.

```
> anova(with(integrationData,
+         glm(isLatent~I(logDist_nearest==0)+logDist_cpg,family='binomial'))
+         ,test='Chisq')
```

```
Analysis of Deviance Table

Model: binomial, link: logit

Response: isLatent

Terms added sequentially (first to last)


                        Df Deviance Resid. Df Resid. Dev  Pr(>Chi)    
NULL                                    12435      17240              
I(logDist_nearest == 0)  1  26.2682     12434      17213 2.971e-07 ***
logDist_cpg              1   1.1328     12433      17212    0.2872    
---
Signif. codes:  0 '***' 0.001 '**' 0.01 '*' 0.05 '.' 0.1 ' ' 1
```

```
> sapply(unique(integrationData$sample),function(x){
+         p.adjust(
+                 anova(with(integrationData[integrationData$sample==x,],
+                         glm(isLatent~I(logDist_nearest==0)+logDist_cpg,family='binomial'))
+                 ,test='Chisq')['logDist_cpg','Pr(>Chi)']
+         ,method='bonferroni',n=5)
+ })
```

```
         Active   Central Memory          Jurkat Bcl-2 transduced 
      1.0000000        1.0000000        1.0000000        1.0000000 
        Resting 
      0.2007788 
```

8 Alphoid repeats

When analyzing repetitive elements, we treated each read as an independent observation and included reads with multiple alignments to the genome. Additional File 3 is a gzipped csv file containing a row for each read with multiple alignments and one row for each dereplicated integration site with a single alignment with the count variable indicating the number of reads dereplicated to that integration site. There should be 26, 190 rows (excluding header) with 14, 494 rows of expressed provirus and 11, 696 rows of silent/inducible provirus.

```
> repeats<-read.csv('AdditionalFile3.csv.gz', check.names=FALSE, stringsAsFactors=FALSE)
> nrow(repeats)
```

```
[1] 26190
```

```
> summary(repeats$isLatent)
```

```
   Mode   FALSE    TRUE    NA's 
logical   14494   11696       0 
```

```
> notRepeatColumns<-c('id', 'isLatent', 'sample', 'count')
```

To analyze whether there was an association between proviral expression status and integration within alphoid repeats, we used Fisher's exact test with a Bonferroni correction for five samples. For comparison, we looked at the association between proviral expression and the other repeats in the RepeatMasker database. We did not Bonferroni correct for the multiple repeat types so that the repeats could be compared with the analysis of alphoid repeats (for which we had an a priori hypothesis for an association with latency).

```
> dummyX<-rep(c(TRUE, FALSE), 2)
> dummyY<-rep(c(TRUE, FALSE), each=2)
> repeatData<-repeats[, !colnames(repeats) %in% notRepeatColumns]
> repeatData<-repeatData[, apply(repeatData, 2, sum)>0]
> testRepeats<-function(x, repeats){
+     sapply(samples, function(thisSample, repeats){
+         selector<-repeats$sample==thisSample
+         repLatent<-rep(repeats$isLatent[selector], repeats$count[selector])
+         repRepeat<-rep(x[selector], repeats$count[selector])
+         fisher.test(table(c(dummyX, repLatent), c(dummyY, repRepeat))-1)$p.value
+     }, repeats)
+ }
> repeatPs<-apply(repeatData, 2,testRepeats,repeats[, notRepeatColumns])
> table(apply(repeatPs*5<.05, 2, sum))

  0   1   2   3
611  76  15   1

> which(apply(repeatPs*5<.05, 2, sum)>=3)

ALR/Alpha
      178

> p.adjust(repeatPs[, 'ALR/Alpha'], 'bonferroni')

          Active   Central Memory           Jurkat Bcl-2 transduced
    5.026890e-02     3.940207e-03     1.027189e-08     1.000000e+00
         Resting
    2.424896e-02
```

9 Neighbors

We looked at all pairs of viruses on the same chromosome separated by no more than a given distance, e.g. 100 bases, either with all samples pooled or split between within sample pairs or between sample pairs.

```
> allNeighbors<-data.frame('id1'=0, 'id2'=0)[0, ]
> ids<-1:nrow(integrationData)
> for(chr in unique(integrationData$chr)){
+         chrSelector<-integrationData$chr==chr
+         neighborPairs<-data.frame('id1'=rep(ids[chrSelector], sum(chrSelector)),
+         'id2'=rep(ids[chrSelector], each=sum(chrSelector)))
+         neighborPairs<-neighborPairs[neighborPairs$id1<neighborPairs$id2, ]
+         allNeighbors<-rbind(allNeighbors, neighborPairs)
+ }
> allNeighbors$dist<-abs(integrationData$pos[allNeighbors$id1]-
+     integrationData$pos[allNeighbors$id2])
> allNeighbors$latent1<-integrationData$isLatent[allNeighbors$id1]
> allNeighbors$latent2<-integrationData$isLatent[allNeighbors$id2]
> allNeighbors$sample1<-integrationData$sample[allNeighbors$id1]
> allNeighbors$sample2<-integrationData$sample[allNeighbors$id2]
> allNeighbors<-allNeighbors[allNeighbors$dist<=1e6, ]
```

The expected number of matching pairs was calculated as $\sum_{j \in \text{samples}} n_{j,d}(\theta_{j,d}\theta_{\neg j,d} + (1-\theta_{j,d})(1-\theta_{\neg j,d}))$ for between sample, $\sum_{j \in \text{samples}} n_{j,d}(\theta_{j,d}^2 + (1-\theta_{j,d})^2)$ for within sample and $n_d(\theta_d^2 + (1-\theta_d)^2)$ for all pairs, where $n_{j,d}$ is the number of pairs of proviruses separated by no more than d base pairs where the first provirus is from sample j, $\theta_{j,d}$ is the proportion of silent/inducible proviruses in sample j appearing in at least one pair of proviruses separated by less than d base pairs and $\neg j$ means all samples except sample j.

```
> dists<-unique(round(10^seq(1, 6, 1)))
> pairings<-do.call(rbind, lapply(dists, function(x, allNeighbors){
+         inSelector<-allNeighbors$dist<=x&allNeighbors$sample1==allNeighbors$sample2
+         outSelector<-allNeighbors$dist<=x&allNeighbors$sample1!=allNeighbors$sample2
+         allSelector<-allNeighbors$dist<=x
+         out<-data.frame('dist'=x,
+         'observedIn'=sum(allNeighbors[inSelector, 'latent1']==
+             allNeighbors[inSelector, 'latent2']),
+         'observedOut'=sum(allNeighbors[outSelector, 'latent1']==
+             allNeighbors[outSelector, 'latent2']),
+         'observedAll'=sum(allNeighbors[allSelector, 'latent1']==
+             allNeighbors[allSelector, 'latent2']),
+         'totalIn'=sum(inSelector),
+         'totalOut'=sum(outSelector),
+         'totalAll'=sum(allSelector)
+     )
+         out$expectedIn<-sum(with(allNeighbors[inSelector, ], sapply(samples, function(x){
+                         inLatent<-c(latent1[sample1==x], latent2[sample2==x])[
+                 !duplicated(c(id1[sample1==x], id2[sample2==x]))]
+                         if(length(inLatent)==0)return(0)
+                         return(sum(sample1==x)*(mean(inLatent)^2+mean(!inLatent)^2))
+         })))
+         out$expectedOut<-sum(with(allNeighbors[outSelector, ], sapply(samples, function(x){
+                         inLatent<-c(latent1[sample1==x], latent2[sample2==x])[
+                 !duplicated(c(id1[sample1==x], id2[sample2==x]))]
+                         outLatent<-c(latent1[sample1!=x], latent2[sample2!=x])[
+                 !duplicated(c(id1[sample1!=x], id2[sample2!=x]))]
+                         if(length(inLatent)==0)return(0)
+                         return(sum(sample1==x)*(mean(inLatent)*mean(outLatent)
+                     +mean(!inLatent)*mean(!outLatent)))
+         })))
+         out$expectedAll<-sum(with(allNeighbors[allSelector, ], {
+                         allLatent<-c(latent1, latent2)[!duplicated(c(id1, id2))]
+                         return(length(latent1)*(mean(allLatent)^2
+                     +mean(!allLatent)^2))
+         }))
+         return(out)
+ }, allNeighbors))
> rownames(pairings)<-pairings$dist
```

To look for more matches than expected by random pairing between neighboring proviruses, we used a one sample Z-test of proportion to compare the observed number of matching pairs with the expected proportion of pairs.

```
> combinations<-c('All'='All', 'Between sample'='Out', 'Within sample'='In')
> lapply(combinations, function(x, pairing){
+     vars<-sprintf(c('observed%s', 'expected%s', 'total%s'), x)
+     expectedProb<-pairing[, vars[2]]/pairing[, vars[3]]
+     prop.test(pairing[, vars[1]], pairing[, vars[3]], p=expectedProb)
+ }, pairings['100', ])
```

```
$All

        1-sample proportions test with continuity correction

data:  pairing[, vars[1]] out of pairing[, vars[3]], null probability expectedProb
X-squared = 13.0021, df = 1, p-value = 0.0003111
alternative hypothesis: true p is not equal to 0.5000141
95 percent confidence interval:
 0.5586837 0.6962353
sample estimates:
   p
0.63


$`Between sample`

        1-sample proportions test with continuity correction

data:  pairing[, vars[1]] out of pairing[, vars[3]], null probability expectedProb
X-squared = 0.2192, df = 1, p-value = 0.6397
alternative hypothesis: true p is not equal to 0.4836763
95 percent confidence interval:
 0.3570532 0.5572662
sample estimates:
        p
0.4554455


$`Within sample`

        1-sample proportions test with continuity correction

data:  pairing[, vars[1]] out of pairing[, vars[3]], null probability expectedProb
X-squared = 24.4456, df = 1, p-value = 7.644e-07
alternative hypothesis: true p is not equal to 0.5561437
95 percent confidence interval:
 0.7140170 0.8776751
sample estimates:
        p
0.8080808
```

10 Compiling this document

This document was generated using R's Sweave function (http://en.wikipedia.org/wiki/Sweave). If you would like to regenerate this document, download Additional Files 2, 3 and 4 and make sure the files are all in the same directory and named AdditionalFile2.csv.gz, AdditionalFile3.csv.gz and AdditionalFile4.Rnw. Then compile by going to that directory and using the commands:
R CMD Sweave AdditionalFile4.Rnw
pdflatex AdditionalFile4.tex

Note that you will need R and LaTeX (and the R package glmnet if you would like to rerun the lasso regressions) installed.
