# Supplementary material for: HIV latency and integration site placement in five cell-based models
Source: Retrovirology. 2013 Aug 16;10:90. doi: 10.1186/1742-4690-10-90 (PMC3765678; doi:10.1186/1742-4690-10-90)
Supplement: Additional file 5 — Genomic feature analysis of Central Memory CD4+ and Bcl-2 transduced CD4+ data. A pdf file reporting the association of genomic features with integrations in the Central Memory CD4 + and Bcl-2 transduced CD4 + datasets following the methods of Berry et al. [62]. [file 1742-4690-10-90-S5.pdf]

Association of Genomic Features with Integration

July 23, 2013

Contents

1 Introduction **2**

2 Preference for Genes **4**
2.1 Acembly Genes 4
2.2 refGenes 6
2.3 ensGenes 8
2.4 genScan Genes 9
2.5 uniGenes 11
2.6 oncogenes 14

3 CpG Island Neighborhoods **16**
3.1 1 kilobase neighborhoods 16
3.2 2 kilobase neighborhoods 17
3.3 5 kilobase neighborhoods 18
3.4 10 kilobase neighborhoods 18
3.5 25 kilobase neighborhoods 19
3.6 50 kilobase neighborhoods 20

4 Gene Density, Expression 'Density', and CpG Island Density **22**
4.1 25 kilobase Window 22
4.2 50 kilobase Window 26
4.3 100 kilobase Window 31
4.4 250 kilobase Window 36
4.5 500 kilobase Window 41
4.6 1 megabase Window 46
4.7 2 megabase Window 51
4.8 4 megabase Window 56
4.9 8 megabase Window 61
4.10 16 megabase Window 66
4.11 32 megabase Window 71

5 Juxtaposition with Gene Start and End Positions **77**
5.1 Acembly Annotations 77
5.2 RefSeq Annotations 81
5.3 genScan Annotations 85
5.4 uniGene Annotations 89

6 GC content **93**

7 Cytobands **94**

1 Introduction

In this document, I examine the association of integration sites with various genomic features.

The data consist of both actual integration sites and sets of control sites, each set chosen to match the spacing (in bases) from the nearest restriction site (according to the direction in which the sequence was read) to an integration site. The numbers of insertion and matching sites for several data sets are shown below:

```
                  type
Origin.of.data.set insertion match
  Bcl-2_Expressed        298   885
  Bcl-2_Inducible        474  1419
  Memory_Expressed      1600  4714
  Memory_Inducible      2980  8807
  Wang-HIV-Avr         21055 82878
  Wang-HIV-Mse         23181 91661
```

The advantage of choosing 'control' sites that match the spacing from the nearest restriction site is that biases due to location and density of restriction sites are eliminated by applying the classical multinomial logit model (reviewed in [2]). This model allows regression procedures to be applied to the study of integration intensity as a function of genomic features. The `clogit` function of the R `survival` library) implements estimation and fitting for such models along with the usual likelihood ratio and Wald tests.

The distribution of relative frequency of insertions across the chromosomes is given in this barplot:

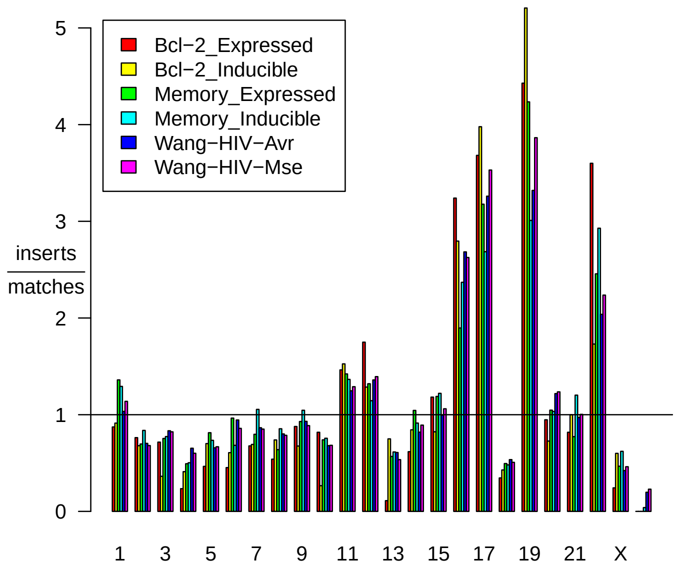

It seems evident that there are some chromosomes that are particularly favored for integration. This is reinforced by a test of statistical significance. The test performed used the likelihood ratio statistic for the multinomial logit model (reviewed in [2]) as implemented by the `clogit` function of the R `survival` library). The null hypothesis tested is that the ratio of true integration events to matched control sites is constant across all chromosomes. This test attains a p-value of $< 2.22e - 16$.

2 Preference for Genes

2.1 Acembly Genes

Here we examine the preference that integration events have for genes. In the following plot we show the relative frequency of integrations in genes according to the 'Acembly' annotation. The bars grouped over the label "In Gene" give the relative frequency of integration events (compared to control sites) between bases located within Acembly gene annotations, while the label "Not in Gene" give the relative frequency of integration events (compared to control sites) between bases not located within Acembly gene annotations.

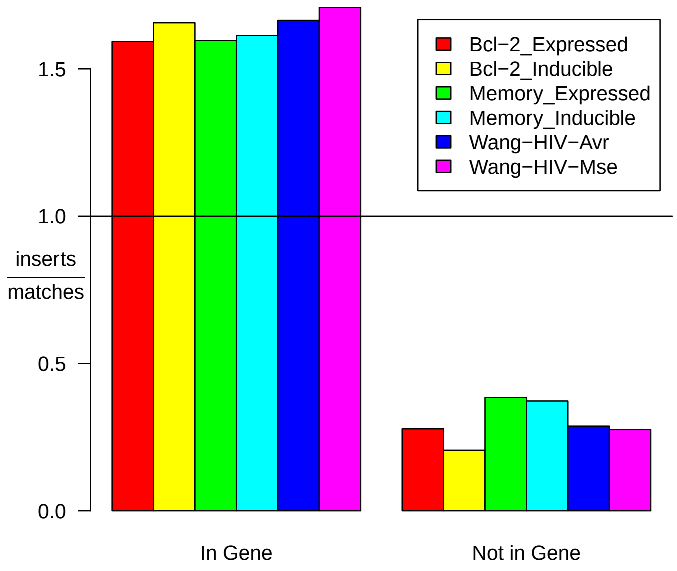

It seems evident that there is a strong tendency for insertions to occur in genes. A formal test of significance bears this out with a p-value of $< 2.22e-16$. Also, it appears that the tendency of different viruses to integrate into genes varies, and a test for this hypothesis attains $1.4558e-12$. Here is the table of coefficients of the log ratio of intensities for true insertion sites versus control insertion sites along with their standard errors, z statistics, and p-values for each data set:

	coef	se	z	p
Bcl-2_Expressed	1.69	0.1890	8.94	3.86e-19
Bcl-2_Inducible	2.09	0.1740	12.00	3.32e-33
Memory_Expressed	1.43	0.0723	19.80	2.93e-87

Memory_Inducible	1.47	0.0533	27.50	9.61e-167
Wang-HIV-Avr	1.76	0.0215	81.70	0.00e+00
Wang-HIV-Mse	1.83	0.0207	88.40	0.00e+00

As is evident, there are some differences in the coefficients. The largest coefficient is seen in the Bcl-2$_{I}nducibledataset, whilethesmallestisseenintheMemory_{E}xpresseddataset.$

In the following plot we show the relative frequency of insertions in exons according to the 'Acembly' annotation. The bars grouped over the label “In Exon” give the relative frequency of integration events (compared to control sites) between bases located in exons according to the Acembly annotation, while the label “Not in Exon” give the relative frequency of integration events (compared to control sites) between bases not located in exons according to the Acembly gene annotation.

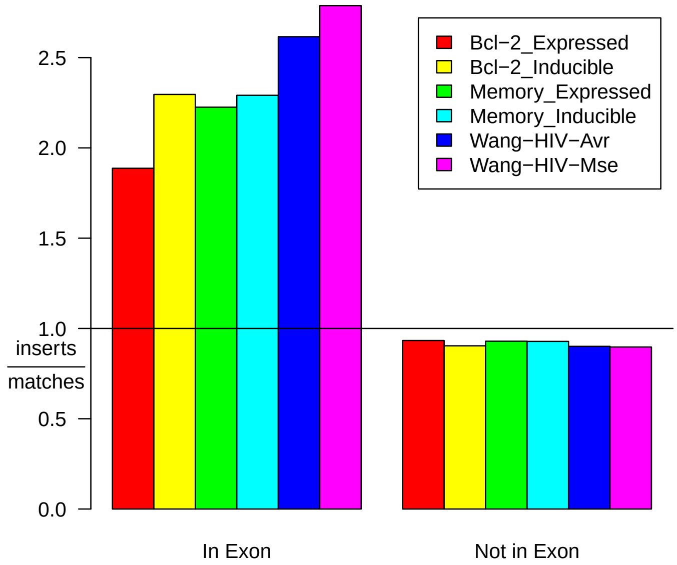

Here is the table of coefficients of the log ratio of intensities for true insertion sites versus control insertion sites along with their standard errors, z statistics, and p-values for each data set:

	coef	se	z	p
Bcl-2_Expressed	0.200	0.2180	0.916	3.60e-01
Bcl-2_Inducible	0.394	0.1750	2.250	2.42e-02
Memory_Expressed	0.364	0.1040	3.500	4.64e-04
Memory_Inducible	0.399	0.0771	5.170	2.30e-07
Wang-HIV-Avr	0.531	0.0254	20.900	5.83e-97

```
Wang-HIV-Mse     0.565 0.0245 23.000 1.53e-117
```

The model on which these coefficients are based include terms for whether the site is in a gene or not. Thus, the effect shown as "In Exon" is net of that due to being in a gene. Note that in the barplot above the 'Not in Exon' bars include both the introns and intergenic regions, so the impression given by the table may differ from that for the barplot.

2.2 refGenes

Here we examine the preference that insertions have for genes. In the following plot we show the relative frequency of insertions in genes according to the 'refGene' annotation.

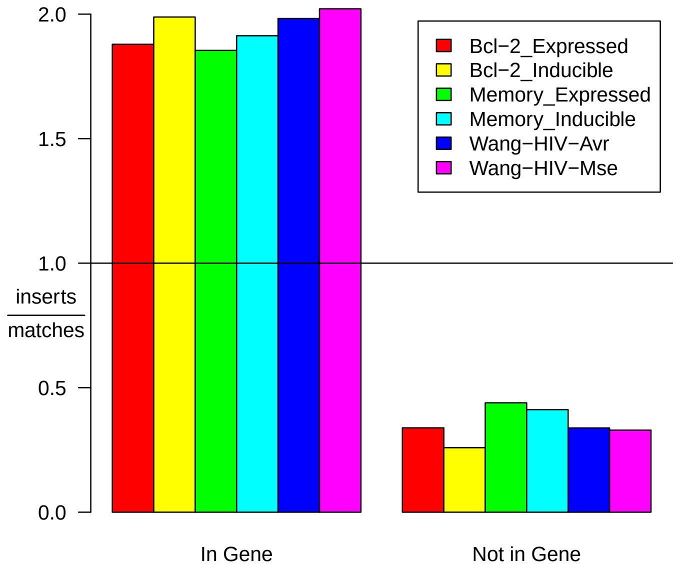

It seems evident that there is a strong tendency for insertions to occur in genes. A formal test of significance bears this out with a p-value of $< 2.22e-16$. Also, it appears that the tendency of different viruses to integrate into genes varies, and a test for this hypothesis attains $1.3276e - 09$. Here is the table of coefficients of the log ratio of intensities for true insertion sites versus control insertion sites along with their standard errors, z statistics, and p-values for each data set:

```
                  coef     se    z          p
Bcl-2_Expressed  1.66 0.1620 10.2  1.48e-24
```

Bcl-2_Inducible	2.02	0.1460	13.8	1.99e-43
Memory_Expressed	1.45	0.0665	21.8	4.67e-105
Memory_Inducible	1.55	0.0500	31.0	4.64e-211
Wang-HIV-Avr	1.77	0.0192	92.5	0.00e+00
Wang-HIV-Mse	1.81	0.0183	98.8	0.00e+00

As is evident, there are some differences in the coefficients. The largest coefficient is seen in the Bcl-2$_{I}nducibledataset, whilethesmallestisseenintheMemory_{E}xpresseddataset.$

In the following plot we show the relative frequency of insertions in exons according to the 'refGene' annotation.

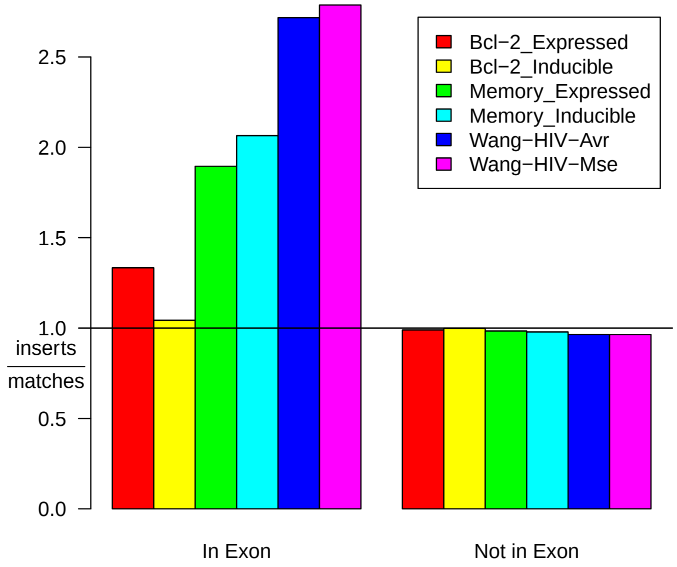

Here is the table of coefficients of the log ratio of intensities for true insertion sites versus control insertion sites along with their standard errors, z statistics, and p-values for each data set:

	coef	se	z	p
Bcl-2_Expressed	-0.25500	0.3560	-0.7140	4.75e-01
Bcl-2_Inducible	-0.70500	0.3100	-2.2800	2.28e-02
Memory_Expressed	0.00566	0.1820	0.0311	9.75e-01
Memory_Inducible	0.08300	0.1230	0.6720	5.01e-01
Wang-HIV-Avr	0.32100	0.0408	7.8800	3.33e-15
Wang-HIV-Mse	0.32500	0.0388	8.3600	6.05e-17

The model on which these coefficients are based include terms for whether

the site is in a gene or not. Thus, the effect shown as ”In Exon” is net of that due to being in a gene.

2.3 ensGenes

Here we examine the preference that insertions have for genes. In the following plot we show the relative frequency of insertions in genes according to the ’ensGene’ annotation.

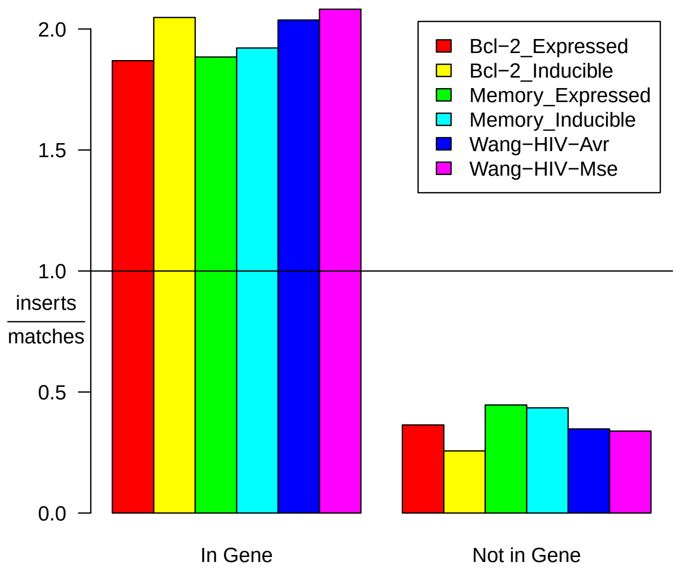

It seems evident that there is a strong tendency for insertions to occur in genes. A formal test of significance bears this out with a p-value of $< 2.22e-16$. Also, it appears that the tendency of different viruses to integrate into genes varies, and a test for this hypothesis attains $6.9371e-13$. Here is the table of coefficients of the log ratio of intensities for true insertion sites versus control insertion sites along with their standard errors, z statistics, and p-values for each data set:

	coef	se	z	p
Bcl-2_Expressed	1.61	0.1600	10.0	1.25e-23
Bcl-2_Inducible	2.08	0.1480	14.0	8.90e-45
Memory_Expressed	1.45	0.0664	21.9	2.15e-106
Memory_Inducible	1.49	0.0488	30.6	4.39e-205
Wang-HIV-Avr	1.77	0.0189	93.8	0.00e+00
Wang-HIV-Mse	1.82	0.0181	100.0	0.00e+00

As is evident, there are some differences in the coefficients. The largest coefficient is seen in the Bcl-2$_{I}nducibledataset, whilethesmallestisseenintheMemory_{E}xpresseddataset.$

In the following plot we show the relative frequency of insertions in exons according to the 'ensGene' annotation.

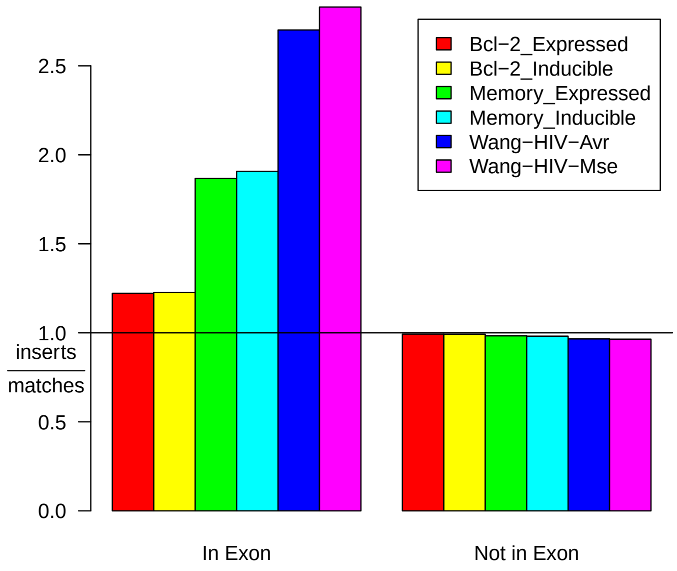

Here is the table of coefficients of the log ratio of intensities for true insertion sites versus control insertion sites along with their standard errors, z statistics, and p-values for each data set:

	coef	se	z	p
Bcl-2_Expressed	-0.3940	0.3690	-1.070	2.86e-01
Bcl-2_Inducible	-0.5680	0.2990	-1.900	5.78e-02
Memory_Expressed	-0.0455	0.1780	-0.256	7.98e-01
Memory_Inducible	0.0254	0.1270	0.201	8.41e-01
Wang-HIV-Avr	0.2870	0.0411	6.980	2.85e-12
Wang-HIV-Mse	0.3180	0.0393	8.080	6.25e-16

The model on which these coefficients are based include terms for whether the site is in a gene or not. Thus, the effect shown as "In Exon" is net of that due to being in a gene.

2.4 genScan Genes

Here we examine the preference that insertions have for genes. In the following plot we show the relative frequency of insertions in genes according to the

'genScan' annotation.

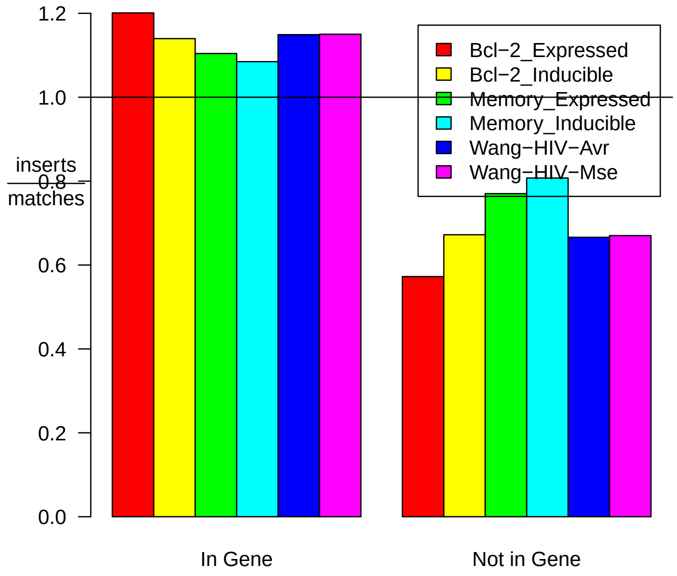

It seems evident that there is a strong tendency for insertions to occur in genes. A formal test of significance bears this out with a p-value of $< 2.22e-16$. Also, it appears that the tendency of different viruses to integrate into genes varies, and a test for this hypothesis attains $9.0126e-06$. Here is the table of coefficients of the log ratio of intensities for true insertion sites versus control insertion sites along with their standard errors, z statistics, and p-values for each data set:

	coef	se	z	p
Bcl-2_Expressed	0.730	0.1670	4.38	1.19e-05
Bcl-2_Inducible	0.515	0.1270	4.05	5.15e-05
Memory_Expressed	0.369	0.0671	5.49	3.91e-08
Memory_Inducible	0.292	0.0485	6.02	1.72e-09
Wang-HIV-Avr	0.544	0.0187	29.20	7.52e-187
Wang-HIV-Mse	0.539	0.0177	30.50	8.43e-205

As is evident, there are some differences in the coefficients. The largest coefficient is seen in the Bcl-$2_Expresseddataset, whilethesmallestisseenintheMemory_Inducibledataset.$

In the following plot we show the relative frequency of insertions in exons according to the 'genScan' annotation.

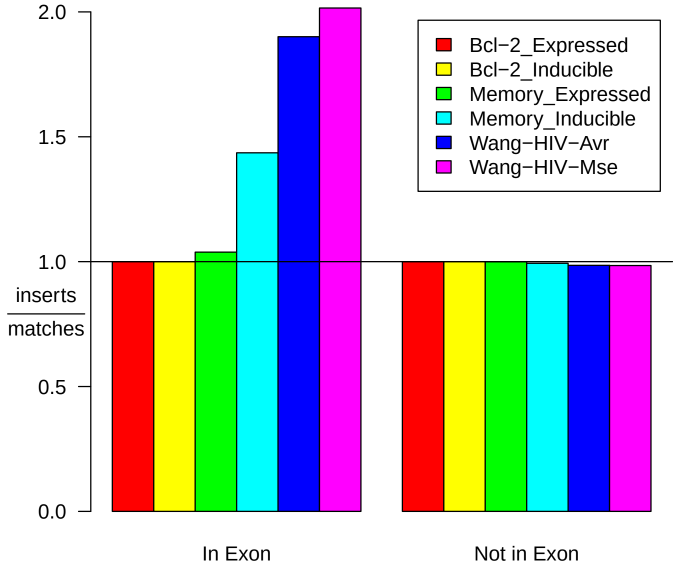

Here is the table of coefficients of the log ratio of intensities for true insertion sites versus control insertion sites along with their standard errors, z statistics, and p-values for each data set:

	coef	se	z	p
Bcl-2_Expressed	-0.2200	0.4750	-0.464	6.43e-01
Bcl-2_Inducible	-0.1360	0.3500	-0.389	6.97e-01
Memory_Expressed	-0.0675	0.2350	-0.287	7.74e-01
Memory_Inducible	0.2890	0.1550	1.860	6.29e-02
Wang-HIV-Avr	0.5240	0.0488	10.700	7.87e-27
Wang-HIV-Mse	0.5740	0.0470	12.200	2.79e-34

The model on which these coefficients are based include terms for whether the site is in a gene or not. Thus, the effect shown as ”In Exon” is net of that due to being in a gene.

2.5 uniGenes

Here we examine the preference that insertions have for genes. In the following plot we show the relative frequency of insertions in genes according to the ’uniGene’ annotation.

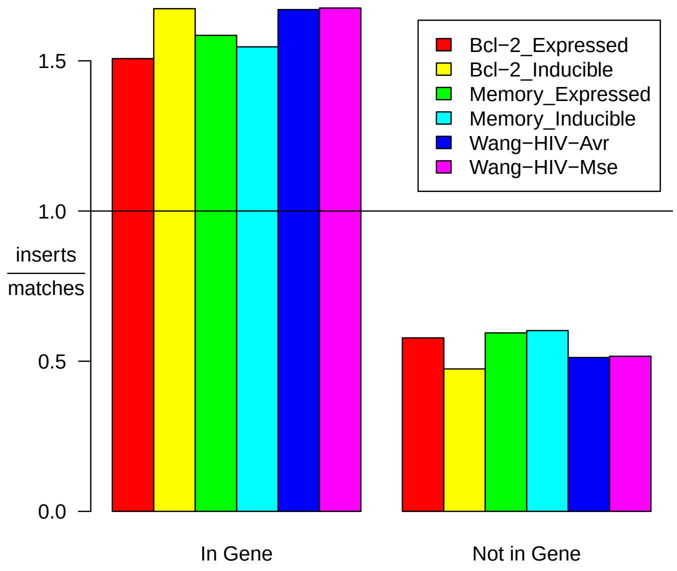

It seems evident that there is a strong tendency for insertions to occur in genes. A formal test of significance bears this out with a p-value of $< 2.22e-16$. Also, it appears that the tendency of different viruses to integrate into genes varies, and a test for this hypothesis attains $1.2543e-07$. Here is the table of coefficients of the log ratio of intensities for true insertion sites versus control insertion sites along with their standard errors, z statistics, and p-values for each data set:

	coef	se	z	p
Bcl-2_Expressed	0.963	0.1450	6.65	2.92e-11
Bcl-2_Inducible	1.240	0.1190	10.40	1.72e-25
Memory_Expressed	0.975	0.0610	16.00	1.41e-57
Memory_Inducible	0.930	0.0445	20.90	7.58e-97
Wang-HIV-Avr	1.180	0.0169	70.00	0.00e+00
Wang-HIV-Mse	1.180	0.0160	73.30	0.00e+00

As is evident, there are some differences in the coefficients. The largest coefficient is seen in the Bcl-2$_{I}nducibledataset, whilethesmallestisseenintheMemory_{I}nducibledataset.$

In the following plot we show the relative frequency of insertions in exons according to the 'uniGene' annotation.

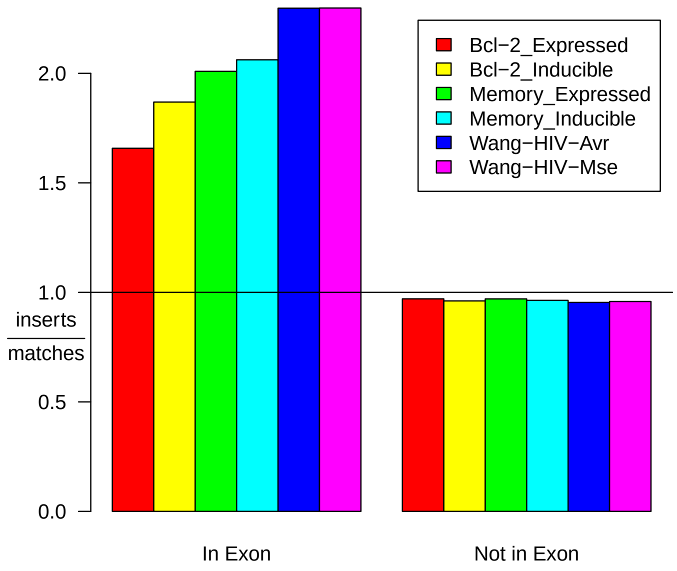

Here is the table of coefficients of the log ratio of intensities for true insertion sites versus control insertion sites along with their standard errors, z statistics, and p-values for each data set:

	coef	se	z	p
Bcl-2_Expressed	0.138	0.2890	0.477	6.33e-01
Bcl-2_Inducible	0.141	0.2220	0.635	5.25e-01
Memory_Expressed	0.271	0.1440	1.880	5.97e-02
Memory_Inducible	0.311	0.0968	3.220	1.29e-03
Wang-HIV-Avr	0.339	0.0330	10.300	8.04e-25
Wang-HIV-Mse	0.346	0.0328	10.600	4.59e-26

The model on which these coefficients are based include terms for whether the site is in a gene or not. Thus, the effect shown as ”In Exon” is net of that due to being in a gene.

2.6 oncogenes

Here we examine the preference that insertions have for oncogenes. In the following plot we show the relative frequency of insertions with 50kb of an oncogene 5' end.

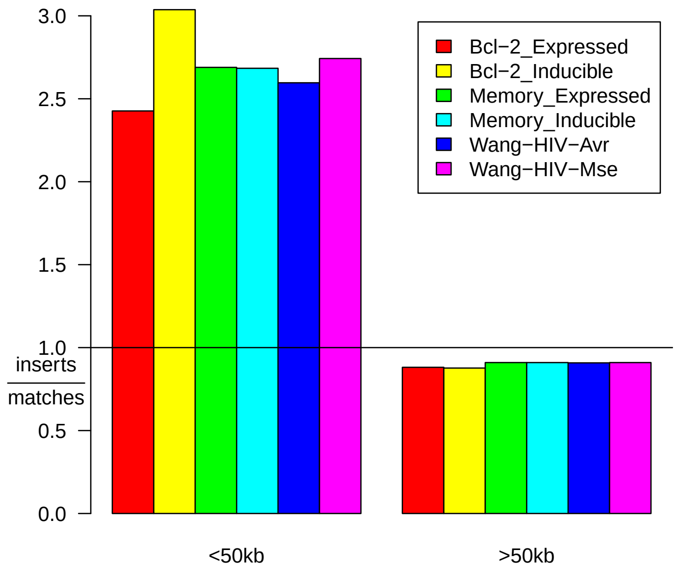

A formal test of oncogenic insertion returns p-value of $< 2.22e-16$. The tendency of different viruses to integrate near oncogenes may vary, and a test for this hypothesis attains 0.68716. Here is the table of coefficients of the log ratio of intensities for true insertion sites versus control insertion sites along with their standard errors, z statistics, and p-values for each data set:

	coef	se	z	p
Bcl-2_Expressed	-1.07	0.2050	-5.20	1.97e-07
Bcl-2_Inducible	-1.19	0.1650	-7.24	4.45e-13
Memory_Expressed	-1.09	0.0997	-10.90	1.39e-27
Memory_Inducible	-1.06	0.0721	-14.70	4.74e-49
Wang-HIV-Avr	-1.05	0.0252	-41.70	0.00e+00
Wang-HIV-Mse	-1.10	0.0248	-44.60	0.00e+00
Bcl-2_Expressed	NA	0.0000	NA	NA
Bcl-2_Inducible	NA	0.0000	NA	NA
Memory_Expressed	NA	0.0000	NA	NA
Memory_Inducible	NA	0.0000	NA	NA
Wang-HIV-Avr	NA	0.0000	NA	NA

```
Wang-HIV-Mse         NA 0.0000     NA       NA
```

As is evident, there are some differences in the coefficients. The largest coefficient is seen in the Wang-HIV-Avr data set, while the smallest is seen in the Bcl-2$_{I}nducibledataset$.

3 CpG Island Neighborhoods

Here we study the effect of being in the neighborhood of CpG Islands. Following Wu et al [3], who found that the neighborhoods within ±1kb of CpG islands are enriched for MLV insertions, we study such neighborhoods.

3.1 1 kilobase neighborhoods

The following plot shows the effect of being in or within ±1kb of a CpG island:

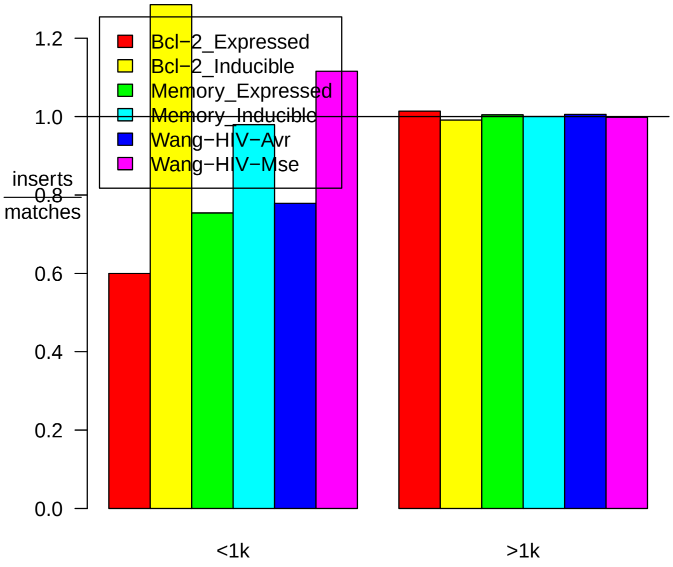

A formal test of significance comparing the difference attains a p-value of 0.019024. A test for differences between viruses attains 0.00011609. Here is the table of coefficients of the log ratio of intensities for true insertion sites versus control insertion sites along with their standard errors, z statistics, and p-values for each data set:

	coef	se	z	p
Bcl-2_Expressed	-0.5280	0.4540	-1.160	2.44e-01
Bcl-2_Inducible	0.2570	0.2850	0.902	3.67e-01
Memory_Expressed	-0.2900	0.2430	-1.200	2.32e-01
Memory_Inducible	-0.0293	0.1800	-0.163	8.70e-01
Wang-HIV-Avr	-0.2560	0.0547	-4.680	2.80e-06
Wang-HIV-Mse	0.1110	0.0557	2.000	4.58e-02

The largest coefficient is seen in the Bcl-2$_{I}nducibledataset, whilethesmallestisseenintheBcl-$ $2_{E}xpresseddataset.$

3.2 2 kilobase neighborhoods

The following plot shows the effect of being in or within ±2kb of a CpG island:

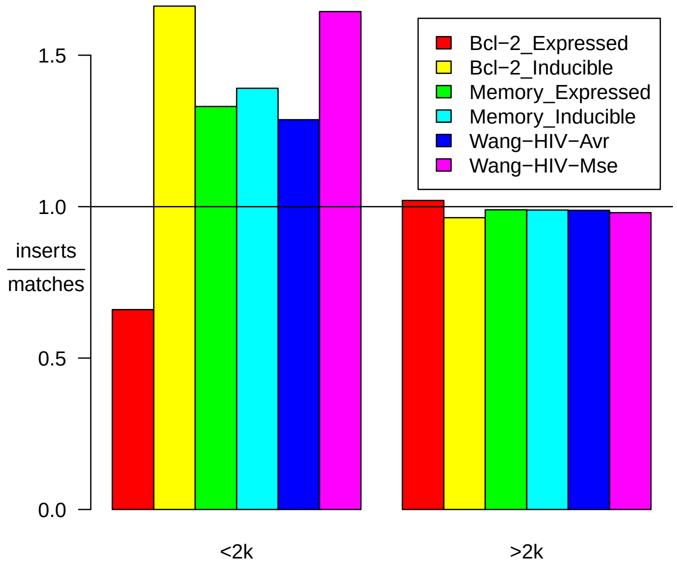

A formal test of significance comparing the difference attains a p-value of $< 2.22e - 16$. A test for differences between viruses attains $4.2006e - 06$. Here is the table of coefficients of the log ratio of intensities for true insertion sites versus control insertion sites along with their standard errors, z statistics, and p-values for each data set:

	coef	se	z	p
Bcl-2_Expressed	-0.432	0.3390	-1.28	2.02e-01
Bcl-2_Inducible	0.526	0.1990	2.65	8.10e-03
Memory_Expressed	0.305	0.1550	1.97	4.84e-02
Memory_Inducible	0.340	0.1150	2.97	2.99e-03
Wang-HIV-Avr	0.264	0.0356	7.41	1.28e-13
Wang-HIV-Mse	0.518	0.0362	14.30	1.25e-46

The largest coefficient is seen in the Bcl-2$_{I}nducibledataset, whilethesmallestisseenintheBcl-$ $2_{E}xpresseddataset.$

3.3 5 kilobase neighborhoods

The following plot shows the effect of being in or within ±5kb of a CpG island:

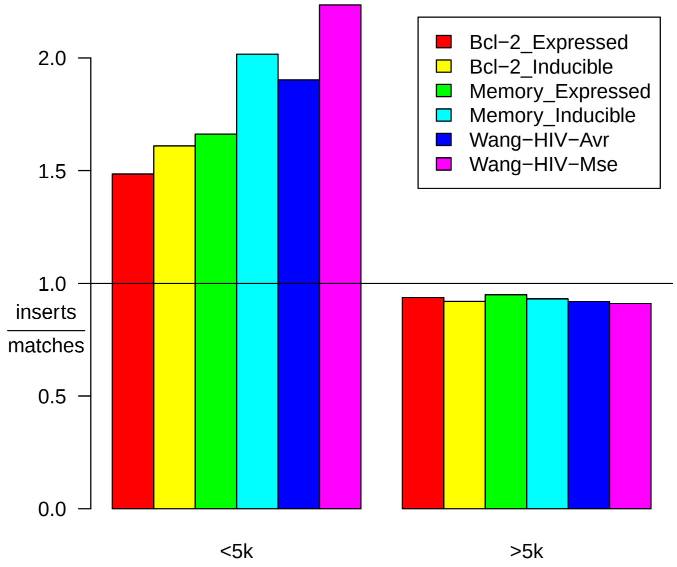

A formal test of significance comparing the difference attains a p-value of $< 2.22e - 16$. A test for differences between viruses attains $3.6905e - 08$. Here is the table of coefficients of the log ratio of intensities for true insertion sites versus control insertion sites along with their standard errors, z statistics, and p-values for each data set:

	coef	se	z	p
Bcl-2_Expressed	0.457	0.1880	2.44	1.49e-02
Bcl-2_Inducible	0.538	0.1420	3.80	1.48e-04
Memory_Expressed	0.560	0.0960	5.83	5.39e-09
Memory_Inducible	0.768	0.0702	10.90	7.74e-28
Wang-HIV-Avr	0.728	0.0229	31.80	4.25e-222
Wang-HIV-Mse	0.901	0.0228	39.60	0.00e+00

The largest coefficient is seen in the Wang-HIV-Mse data set, while the smallest is seen in the Bcl-$2_Expresseddataset.$

3.4 10 kilobase neighborhoods

The following plot shows the effect of being in or within ±10kb of a CpG island:

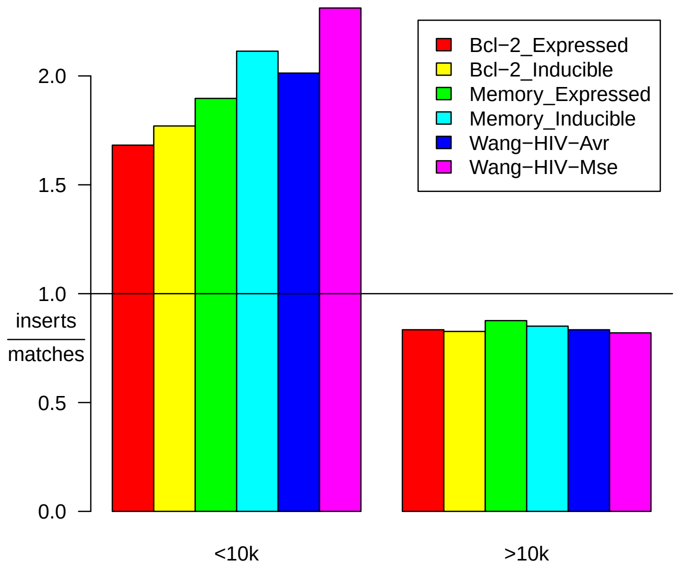

A formal test of significance comparing the difference attains a p-value of $< 2.22e - 16$. A test for differences between viruses attains $2.2187e - 10$. Here is the table of coefficients of the log ratio of intensities for true insertion sites versus control insertion sites along with their standard errors, z statistics, and p-values for each data set:

	coef	se	z	p
Bcl-2_Expressed	0.689	0.1500	4.60	4.33e-06
Bcl-2_Inducible	0.746	0.1190	6.27	3.71e-10
Memory_Expressed	0.760	0.0743	10.20	1.45e-24
Memory_Inducible	0.916	0.0546	16.80	3.29e-63
Wang-HIV-Avr	0.880	0.0184	47.80	0.00e+00
Wang-HIV-Mse	1.040	0.0181	57.60	0.00e+00

The largest coefficient is seen in the Wang-HIV-Mse data set, while the smallest is seen in the Bcl-2$_E xpresseddataset.$

3.5 25 kilobase neighborhoods

The following plot shows the effect of being in or within ±25kb of a CpG island:

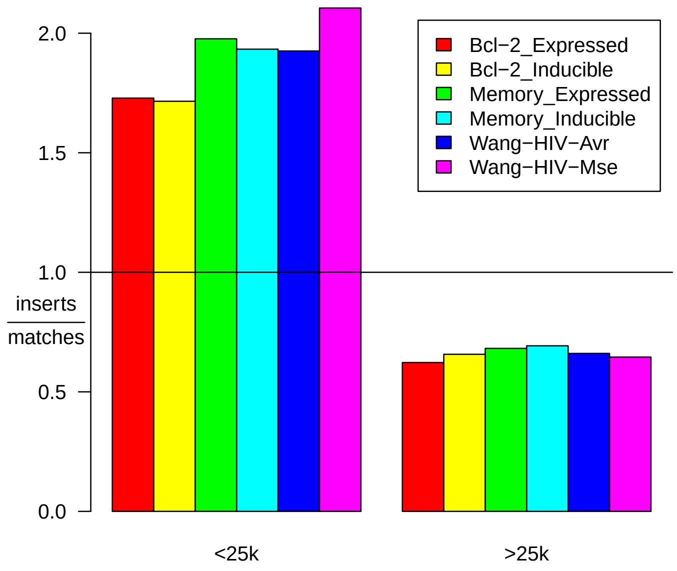

A formal test of significance comparing the difference attains a p-value of $< 2.22e - 16$. A test for differences between viruses attains $1.1841e - 06$. Here is the table of coefficients of the log ratio of intensities for true insertion sites versus control insertion sites along with their standard errors, z statistics, and p-values for each data set:

	coef	se	z	p
Bcl-2_Expressed	0.999	0.1390	7.21	5.61e-13
Bcl-2_Inducible	0.968	0.1110	8.72	2.71e-18
Memory_Expressed	1.060	0.0618	17.20	4.49e-66
Memory_Inducible	1.020	0.0449	22.70	3.36e-114
Wang-HIV-Avr	1.070	0.0162	66.50	0.00e+00
Wang-HIV-Mse	1.190	0.0156	76.30	0.00e+00

The largest coefficient is seen in the Wang-HIV-Mse data set, while the smallest is seen in the Bcl-2$_{I}nducibledataset$.

3.6 50 kilobase neighborhoods

The following plot shows the effect of being in or within ±50kb of a CpG island:

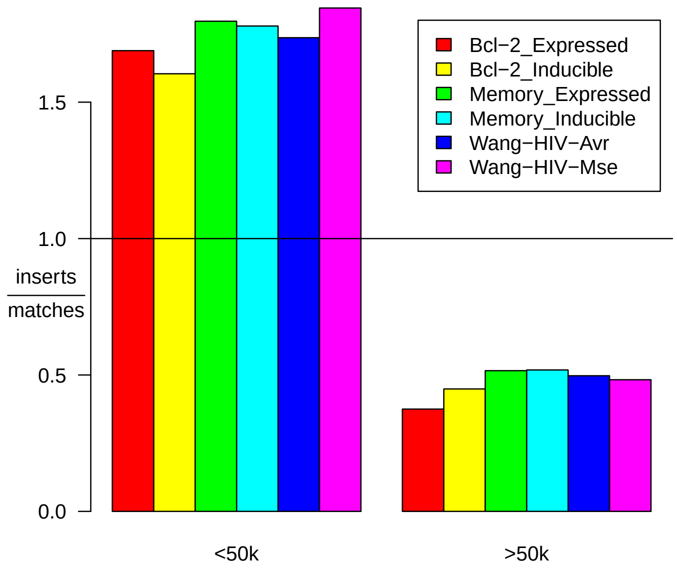

A formal test of significance comparing the difference attains a p-value of $< 2.22e - 16$. A test for differences between viruses attains 0.00091194. Here is the table of coefficients of the log ratio of intensities for true insertion sites versus control insertion sites along with their standard errors, z statistics, and p-values for each data set:

	coef	se	z	p
Bcl-2_Expressed	1.49	0.1640	9.07	1.15e-19
Bcl-2_Inducible	1.24	0.1210	10.30	1.01e-24
Memory_Expressed	1.25	0.0634	19.70	3.12e-86
Memory_Inducible	1.22	0.0458	26.60	9.52e-156
Wang-HIV-Avr	1.25	0.0170	73.70	0.00e+00
Wang-HIV-Mse	1.35	0.0162	82.90	0.00e+00

The largest coefficient is seen in the Bcl-2$_{E}xpresseddataset, whilethesmallestisseenintheMemory_{I}nducible$

4 Gene Density, Expression 'Density', and CpG Island Density

In this section the association with gene density is examined. For expression analysis, the 'genes' that are counted are the genes represented on the microarray. In addition, we the number of such genes expressed at various levels. The levels are

low.ex Count genes whose expression is in the upper half and divide by number of bases

med.ex Count genes whose expression is in the upper $1/8^{th}$ and divide by number of bases

high.ex Count genes whose expression is in the upper $1/16^{th}$ and divide by number of bases

The bolded terms are used as abbreviations in what follows. The abbreviation **dens** is used to indicate gene density as number of genes per base.

4.1 25 kilobase Window

In the barplot that follows we examine the association of insertion sites with gene density in a 25 kilobase window surrounding each locus. More such plots will follow and the method of their construction is always to try to divide the data according to the deciles of density. However, it often happens that there is a very skewed distribution of density and even the 90th percentile is zero. In that case, the barplots simply show the sites for which the density is zero and those for which it is non-zero. If there are fewer than ten groups of bars, the groupings contain ten percent of the sites each except for the leftmost grouping which will contain all of the remaining sites.

Also note that the title of the plot contains clues as to its content; the prefix indicates the type of variable studied while the suffix indicates the window width in the number of bases. The p-value given is the result of fitting a cubic polynomial to the gene density values.

The following expression data and probe set were used for this report:

```
[1] "Jurkat-HU133Plus2"
```

```
[1] "HG-U133"
```

```
                  coef     se    z         p
Bcl-2_Expressed   1.19 0.1420  8.4  4.36e-17
Bcl-2_Inducible   1.46 0.1180 12.3  6.96e-35
Memory_Expressed  1.25 0.0627 19.9  2.64e-88
Memory_Inducible  1.31 0.0464 28.3 4.54e-176
Wang-HIV-Avr      1.37 0.0165 83.2  0.00e+00
Wang-HIV-Mse      1.44 0.0158 91.1  0.00e+00
```

Here are the results for expression density. First, we count just genes that are in the upper half.

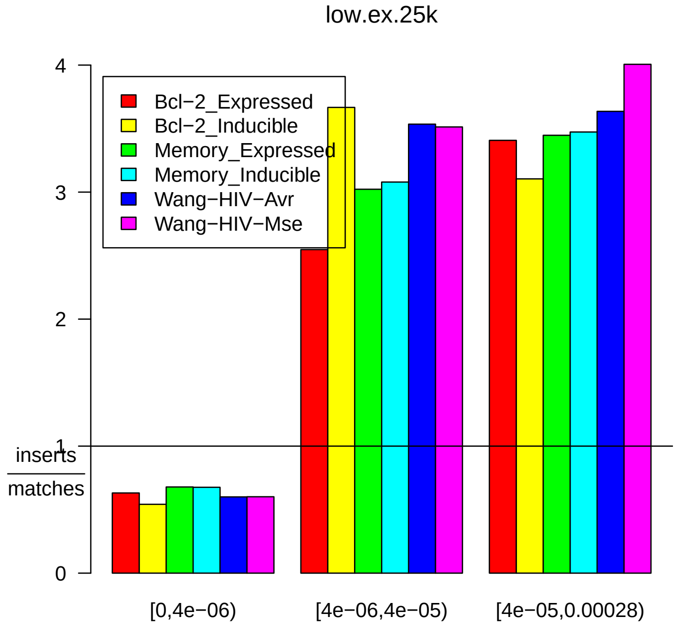

	coef	se	z	p
Bcl-2_Expressed	1.63	0.1630	9.99	1.66e-23
Bcl-2_Inducible	1.82	0.1260	14.40	4.95e-47
Memory_Expressed	1.58	0.0714	22.10	2.89e-108
Memory_Inducible	1.58	0.0520	30.40	2.70e-203
Wang-HIV-Avr	1.78	0.0181	98.60	0.00e+00
Wang-HIV-Mse	1.84	0.0176	105.00	0.00e+00

Now we count genes in the upper $1/8^{th}$:

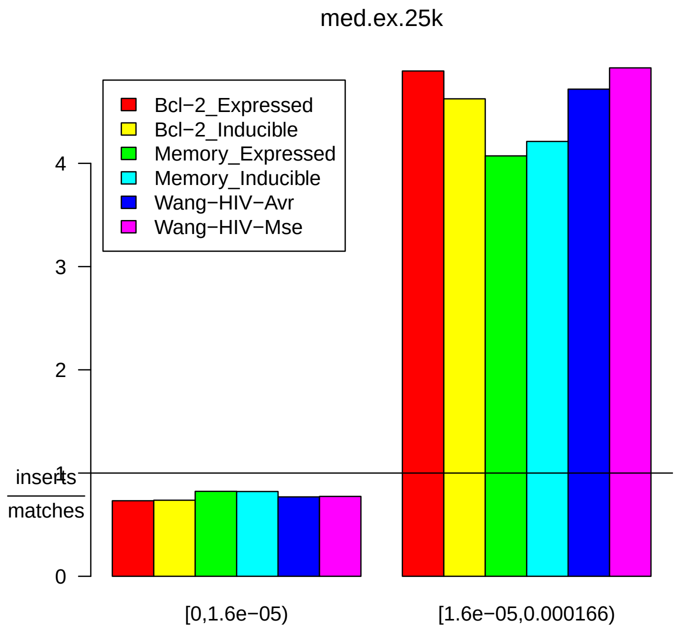

	coef	se	z	p
Bcl-2_Expressed	1.74	0.1780	9.81	1.03e-22
Bcl-2_Inducible	1.98	0.1450	13.70	1.64e-42
Memory_Expressed	1.61	0.0815	19.80	2.51e-87
Memory_Inducible	1.63	0.0597	27.40	2.67e-165
Wang-HIV-Avr	1.91	0.0205	93.40	0.00e+00
Wang-HIV-Mse	1.93	0.0198	97.30	0.00e+00

And here we count genes in the upper $1/16^{th}$:

```
Density data too sparse for barplot

                  coef     se     z         p
Bcl-2_Expressed   1.62 0.2150  7.56  4.06e-14
Bcl-2_Inducible   1.97 0.1670 11.80  4.40e-32
Memory_Expressed  1.76 0.1020 17.30  7.03e-67
Memory_Inducible  1.72 0.0762 22.50 1.49e-112
Wang-HIV-Avr      1.87 0.0245 76.20  0.00e+00
Wang-HIV-Mse      1.92 0.0240 80.20  0.00e+00
```

Here the effect of density of CpG islands is studied:

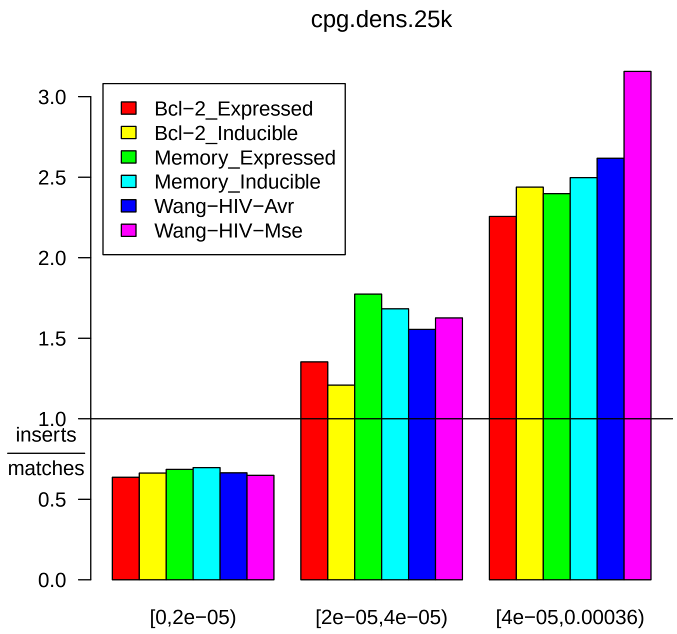

	coef	se	z	p
Bcl-2_Expressed	0.960	0.1380	6.96	3.34e-12
Bcl-2_Inducible	0.953	0.1110	8.62	6.82e-18
Memory_Expressed	1.060	0.0620	17.10	1.87e-65
Memory_Inducible	1.020	0.0450	22.60	6.83e-113
Wang-HIV-Avr	1.070	0.0162	66.20	0.00e+00
Wang-HIV-Mse	1.190	0.0156	76.00	0.00e+00

4.2 50 kilobase Window

In the barplot that follows we examine the association of insertion sites with expression density in a 50 kilobase window surrounding each locus. First, we count just the number of genes represented on the chip.

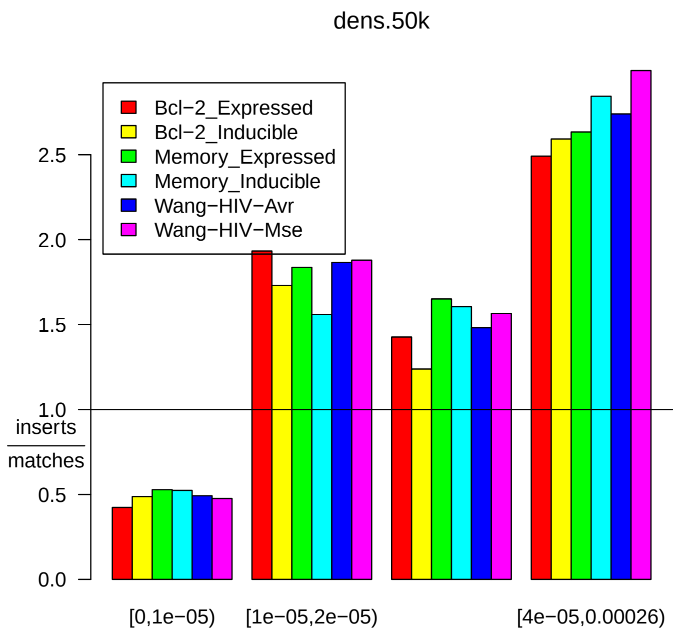

	coef	se	z	p
Bcl-2_Expressed	1.47	0.1580	9.31	1.24e-20
Bcl-2_Inducible	1.57	0.1280	12.20	1.72e-34
Memory_Expressed	1.49	0.0660	22.50	4.15e-112
Memory_Inducible	1.45	0.0475	30.40	1.75e-203
Wang-HIV-Avr	1.53	0.0177	86.10	0.00e+00
Wang-HIV-Mse	1.60	0.0170	94.40	0.00e+00

Here are the results for expression density. First, we count just genes that are in the upper half.

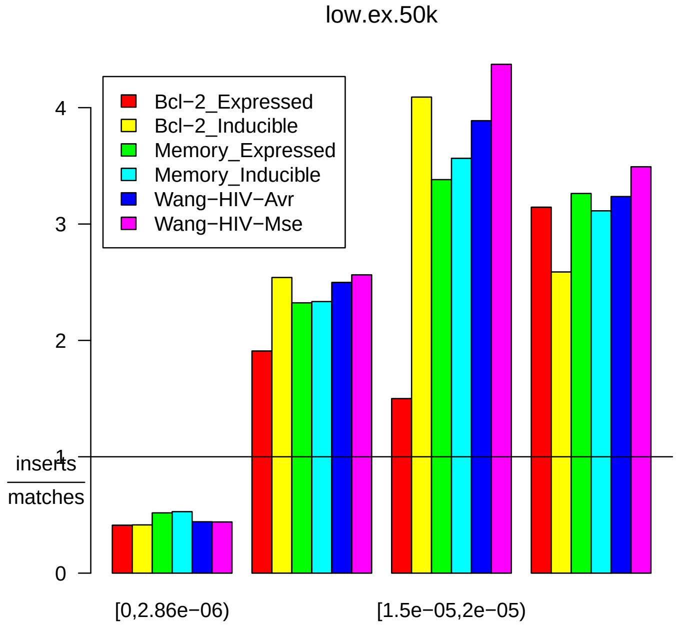

	coef	se	z	p
Bcl-2_Expressed	1.87	0.1600	11.7	1.15e-31
Bcl-2_Inducible	1.88	0.1270	14.8	1.40e-49
Memory_Expressed	1.75	0.0677	25.8	1.13e-146
Memory_Inducible	1.67	0.0484	34.5	4.33e-260
Wang-HIV-Avr	1.91	0.0177	108.0	0.00e+00
Wang-HIV-Mse	1.99	0.0172	116.0	0.00e+00

Now we count genes in the upper $1/8^{th}$:

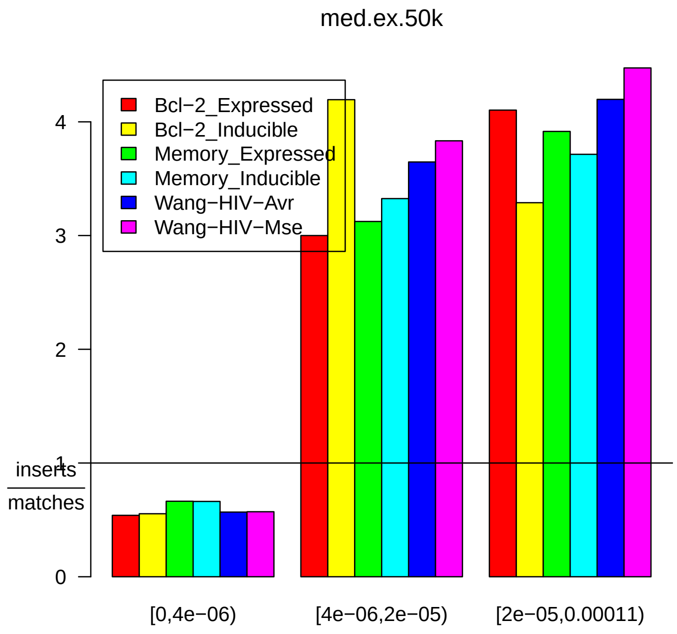

	coef	se	z	p
Bcl-2_Expressed	1.90	0.1630	11.6	2.32e-31
Bcl-2_Inducible	1.97	0.1310	15.0	4.48e-51
Memory_Expressed	1.68	0.0715	23.6	9.53e-123
Memory_Inducible	1.66	0.0515	32.2	4.04e-228
Wang-HIV-Avr	1.95	0.0183	107.0	0.00e+00
Wang-HIV-Mse	2.02	0.0177	114.0	0.00e+00

And here we count genes in the upper $1/16^{th}$:

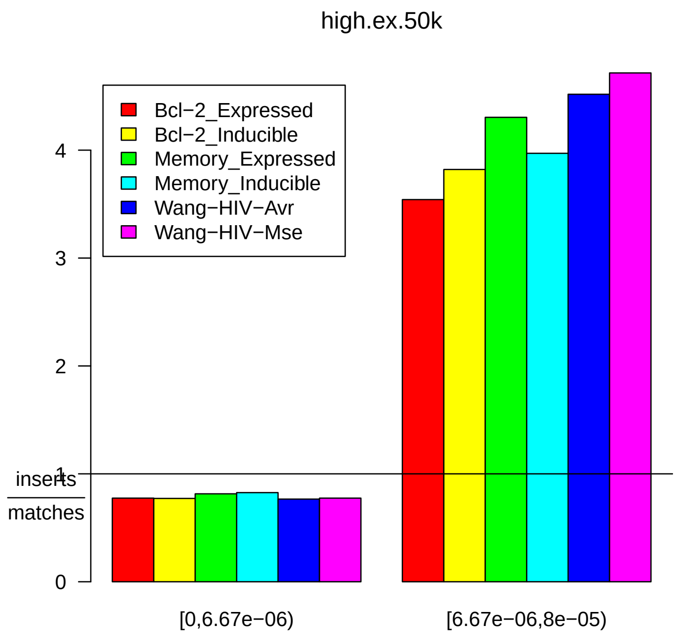

	coef	se	z	p
Bcl-2_Expressed	1.48	0.1720	8.63	6.29e-18
Bcl-2_Inducible	1.82	0.1390	13.00	7.99e-39
Memory_Expressed	1.67	0.0831	20.10	4.89e-90
Memory_Inducible	1.61	0.0606	26.60	3.34e-156
Wang-HIV-Avr	1.82	0.0202	90.10	0.00e+00
Wang-HIV-Mse	1.89	0.0198	95.10	0.00e+00

Here the effect of density of CpG islands is studied:

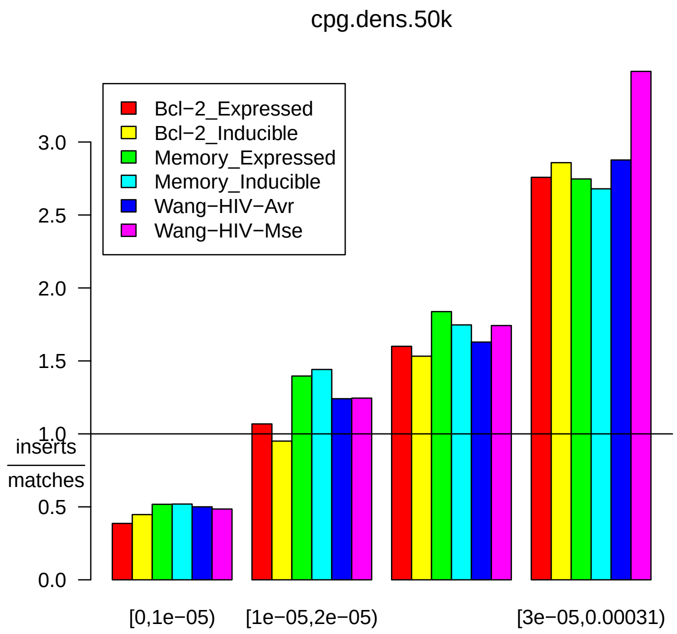

	coef	se	z	p
Bcl-2_Expressed	1.45	0.1620	8.97	2.95e-19
Bcl-2_Inducible	1.25	0.1210	10.30	5.56e-25
Memory_Expressed	1.25	0.0634	19.70	3.22e-86
Memory_Inducible	1.22	0.0458	26.70	1.46e-156
Wang-HIV-Avr	1.25	0.0170	73.60	0.00e+00
Wang-HIV-Mse	1.34	0.0162	82.80	0.00e+00

4.3 100 kilobase Window

In the barplot that follows we examine the association of insertion sites with expression density in a 100 kilobase window surrounding each locus. First, we count just the number of genes represented on the chip.

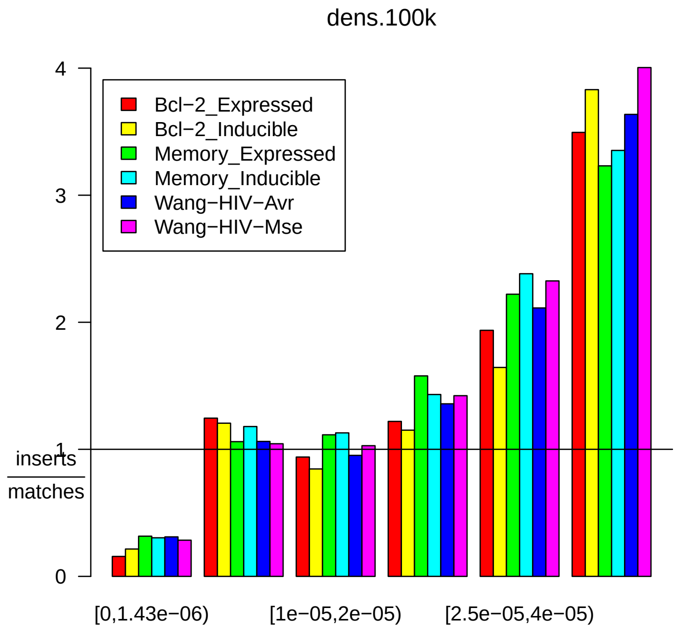

	coef	se	z	p
Bcl-2_Expressed	1.56	0.1520	10.3	8.94e-25
Bcl-2_Inducible	1.57	0.1200	13.1	2.17e-39
Memory_Expressed	1.37	0.0630	21.7	3.19e-104
Memory_Inducible	1.38	0.0461	30.0	1.69e-197
Wang-HIV-Avr	1.47	0.0168	87.3	0.00e+00
Wang-HIV-Mse	1.57	0.0162	97.0	0.00e+00

Here are the results for expression density. First, we count just genes that are in the upper half.

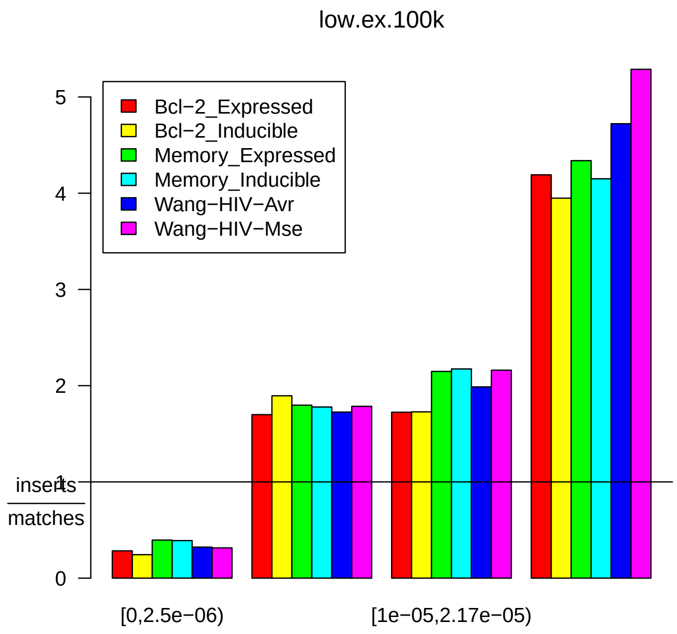

	coef	se	z	p
Bcl-2_Expressed	2.12	0.1830	11.5	7.61e-31
Bcl-2_Inducible	2.39	0.1620	14.7	4.59e-49
Memory_Expressed	1.83	0.0690	26.5	7.28e-155
Memory_Inducible	1.86	0.0517	36.0	8.90e-284
Wang-HIV-Avr	2.06	0.0195	105.0	0.00e+00
Wang-HIV-Mse	2.15	0.0188	114.0	0.00e+00

Now we count genes in the upper $1/8^{th}$:

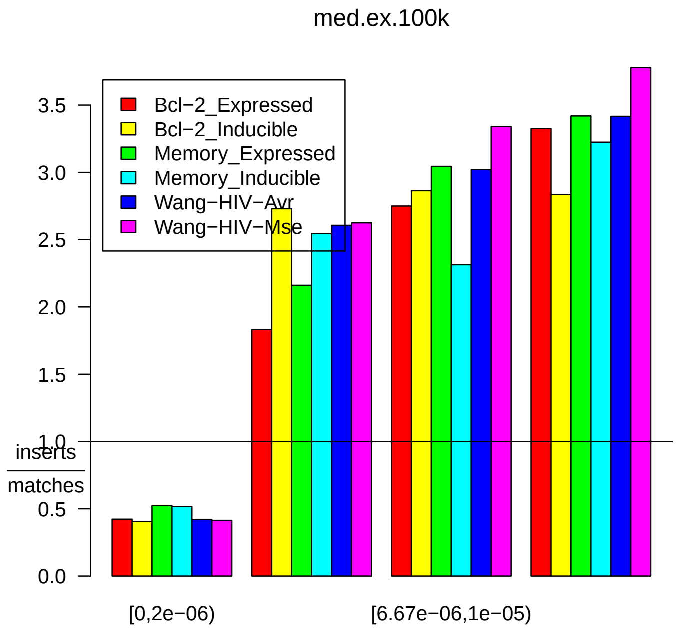

	coef	se	z	p
Bcl-2_Expressed	1.84	0.1550	11.8	2.95e-32
Bcl-2_Inducible	1.95	0.1270	15.3	4.29e-53
Memory_Expressed	1.76	0.0676	26.0	7.12e-149
Memory_Inducible	1.72	0.0485	35.4	3.76e-275
Wang-HIV-Avr	2.03	0.0183	111.0	0.00e+00
Wang-HIV-Mse	2.12	0.0176	120.0	0.00e+00

And here we count genes in the upper $1/16^{th}$:

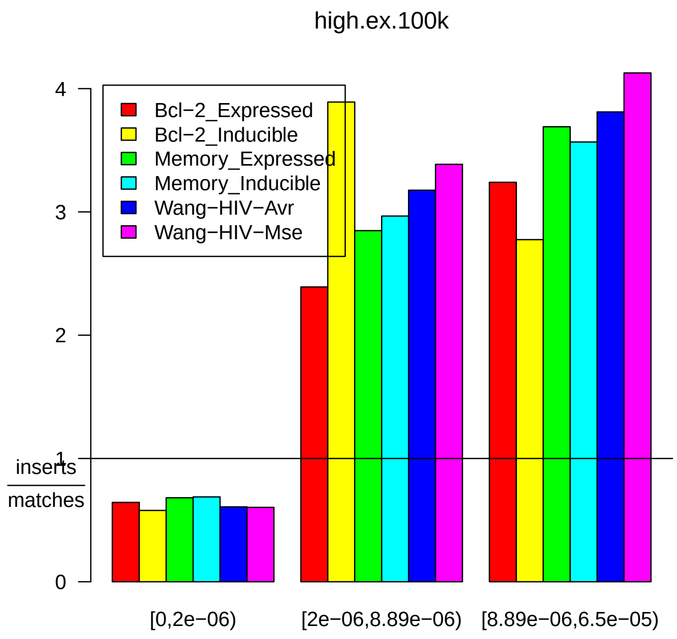

	coef	se	z	p
Bcl-2_Expressed	1.45	0.1530	9.47	2.87e-21
Bcl-2_Inducible	1.80	0.1270	14.10	2.38e-45
Memory_Expressed	1.57	0.0706	22.20	6.99e-109
Memory_Inducible	1.55	0.0514	30.20	2.21e-200
Wang-HIV-Avr	1.75	0.0179	98.00	0.00e+00
Wang-HIV-Mse	1.85	0.0175	106.00	0.00e+00

Here the effect of density of CpG islands is studied:

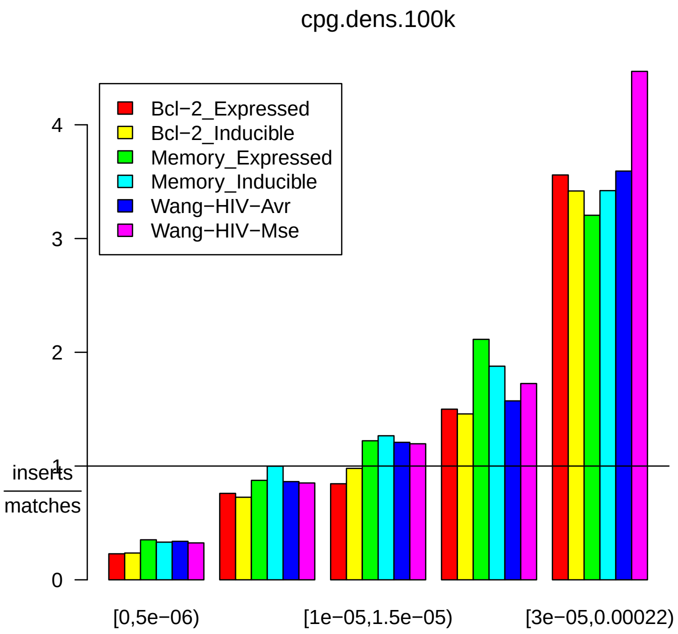

	coef	se	z	p
Bcl-2_Expressed	1.45	0.1540	9.41	5.02e-21
Bcl-2_Inducible	1.58	0.1290	12.30	7.80e-35
Memory_Expressed	1.36	0.0634	21.50	8.07e-103
Memory_Inducible	1.26	0.0453	27.70	3.86e-169
Wang-HIV-Avr	1.32	0.0167	79.10	0.00e+00
Wang-HIV-Mse	1.45	0.0161	90.00	0.00e+00

4.4 250 kilobase Window

In the barplot that follows we examine the association of insertion sites with expression density in a 250 kilobase window surrounding each locus. First, we count just the number of genes represented on the chip.

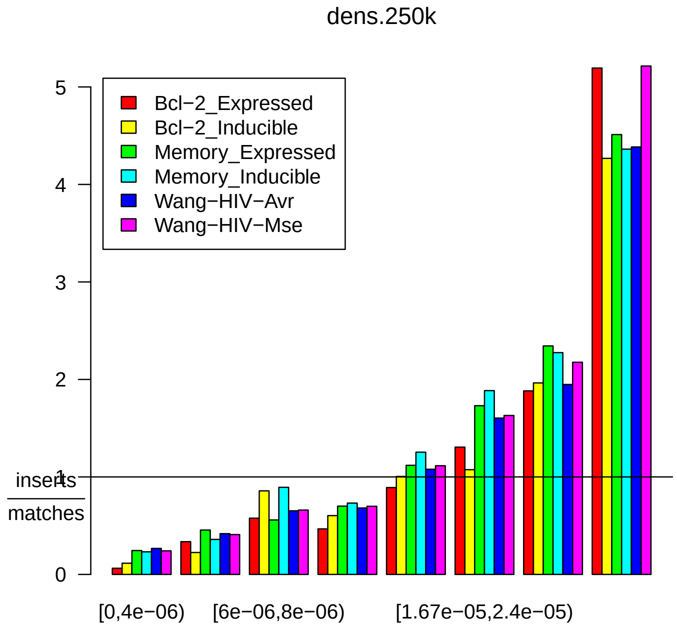

	coef	se	z	p
Bcl-2_Expressed	2.06	0.1970	10.5	1.36e-25
Bcl-2_Inducible	1.89	0.1520	12.4	1.53e-35
Memory_Expressed	1.62	0.0697	23.2	5.10e-119
Memory_Inducible	1.64	0.0510	32.2	2.28e-227
Wang-HIV-Avr	1.57	0.0185	84.6	0.00e+00
Wang-HIV-Mse	1.69	0.0179	94.6	0.00e+00

Here are the results for expression density. First, we count just genes that are in the upper half.

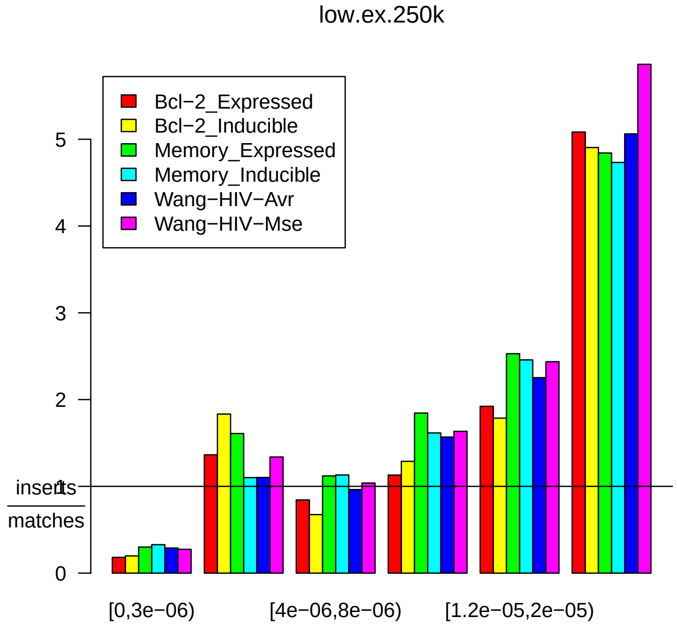

	coef	se	z	p
Bcl-2_Expressed	2.21	0.2060	10.7	9.63e-27
Bcl-2_Inducible	2.15	0.1660	12.9	3.40e-38
Memory_Expressed	1.90	0.0752	25.3	1.30e-141
Memory_Inducible	1.75	0.0532	32.9	1.17e-237
Wang-HIV-Avr	1.88	0.0202	93.1	0.00e+00
Wang-HIV-Mse	2.00	0.0195	103.0	0.00e+00

Now we count genes in the upper $1/8^{th}$:

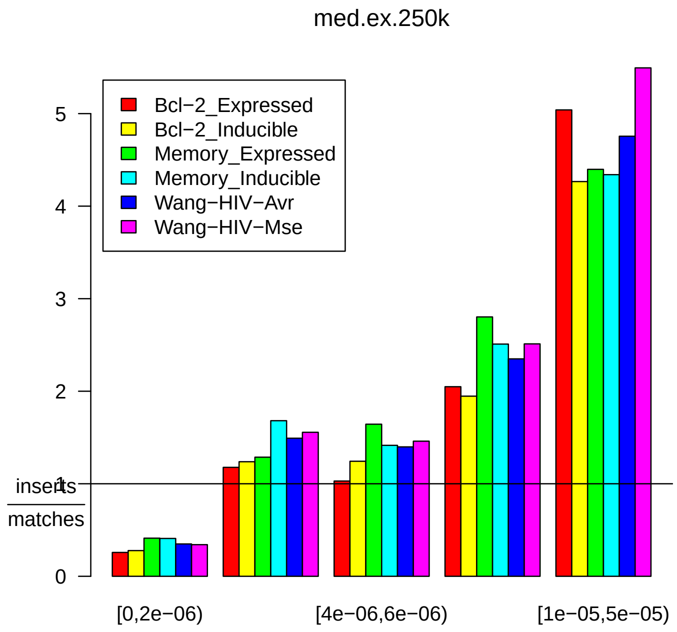

	coef	se	z	p
Bcl-2_Expressed	2.33	0.2090	11.2	5.96e-29
Bcl-2_Inducible	2.29	0.1650	13.9	1.15e-43
Memory_Expressed	1.85	0.0726	25.4	1.15e-142
Memory_Inducible	1.84	0.0533	34.5	2.95e-261
Wang-HIV-Avr	2.04	0.0205	99.7	0.00e+00
Wang-HIV-Mse	2.14	0.0197	109.0	0.00e+00

And here we count genes in the upper $1/16^{th}$:

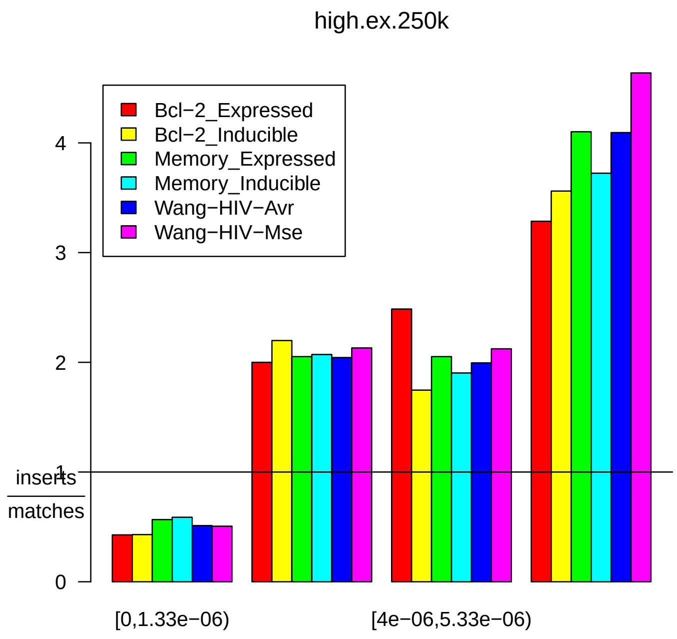

	coef	se	z	p
Bcl-2_Expressed	1.82	0.1580	11.5	1.81e-30
Bcl-2_Inducible	1.97	0.1320	14.9	1.81e-50
Memory_Expressed	1.59	0.0653	24.4	2.18e-131
Memory_Inducible	1.55	0.0474	32.7	7.41e-234
Wang-HIV-Avr	1.75	0.0175	100.0	0.00e+00
Wang-HIV-Mse	1.85	0.0170	109.0	0.00e+00

Here the effect of density of CpG islands is studied:

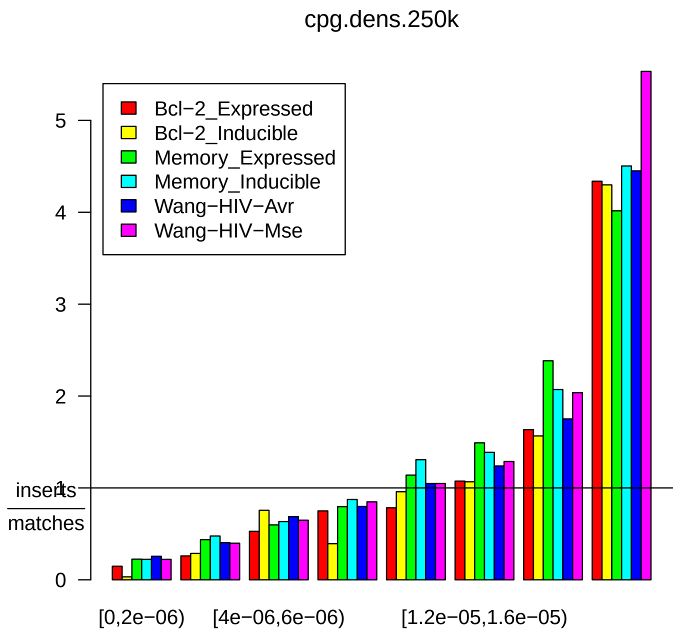

	coef	se	z	p
Bcl-2_Expressed	1.56	0.1640	9.51	1.84e-21
Bcl-2_Inducible	1.84	0.1440	12.70	5.09e-37
Memory_Expressed	1.52	0.0660	23.00	2.11e-117
Memory_Inducible	1.45	0.0476	30.50	8.57e-204
Wang-HIV-Avr	1.38	0.0172	79.90	0.00e+00
Wang-HIV-Mse	1.52	0.0166	91.60	0.00e+00

4.5 500 kilobase Window

In the barplot that follows we examine the association of insertion sites with expression density in a 500 kilobase window surrounding each locus. First, we count just the number of genes represented on the chip.

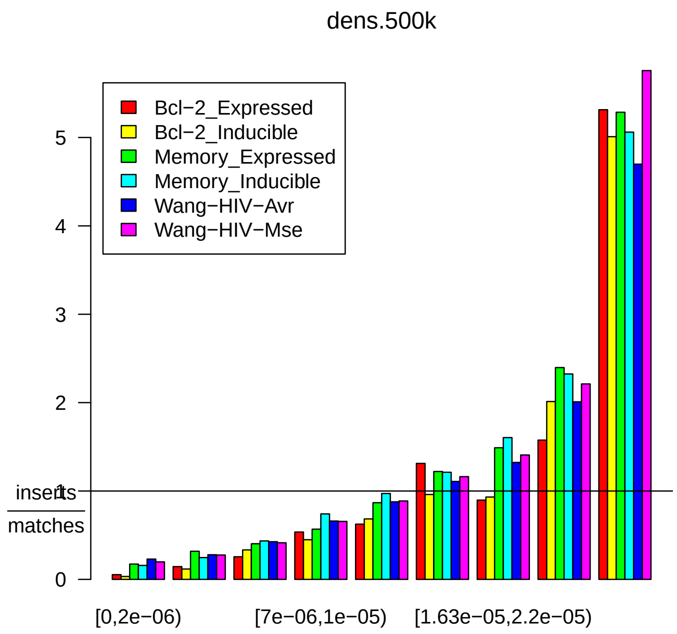

	coef	se	z	p
Bcl-2_Expressed	1.88	0.1820	10.3	6.36e-25
Bcl-2_Inducible	2.06	0.1600	12.9	4.73e-38
Memory_Expressed	1.65	0.0687	23.9	1.13e-126
Memory_Inducible	1.58	0.0496	31.8	2.16e-222
Wang-HIV-Avr	1.52	0.0181	84.4	0.00e+00
Wang-HIV-Mse	1.63	0.0173	94.5	0.00e+00

Here are the results for expression density. First, we count just genes that are in the upper half.

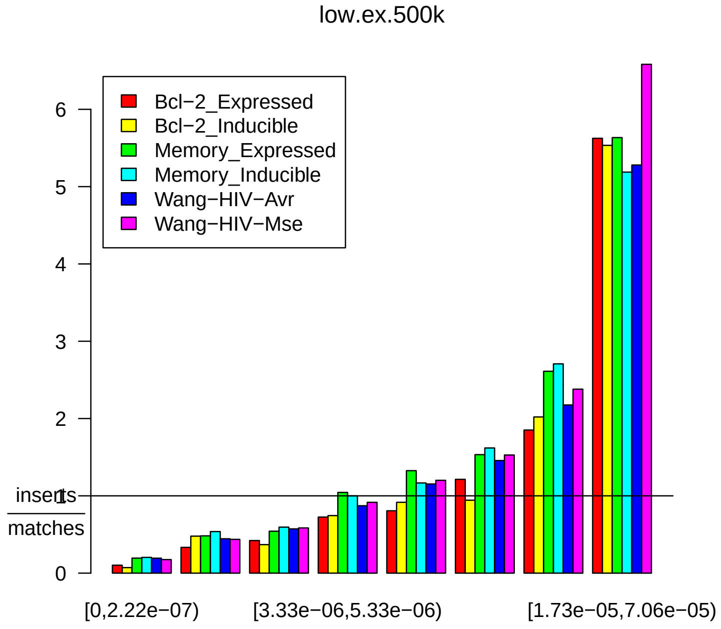

	coef	se	z	p
Bcl-2_Expressed	2.07	0.2010	10.3	5.03e-25
Bcl-2_Inducible	2.28	0.1770	12.8	1.22e-37
Memory_Expressed	1.85	0.0734	25.2	1.04e-139
Memory_Inducible	1.75	0.0532	32.9	7.19e-237
Wang-HIV-Avr	1.76	0.0195	89.9	0.00e+00
Wang-HIV-Mse	1.87	0.0188	99.4	0.00e+00

Now we count genes in the upper $1/8^{th}$:

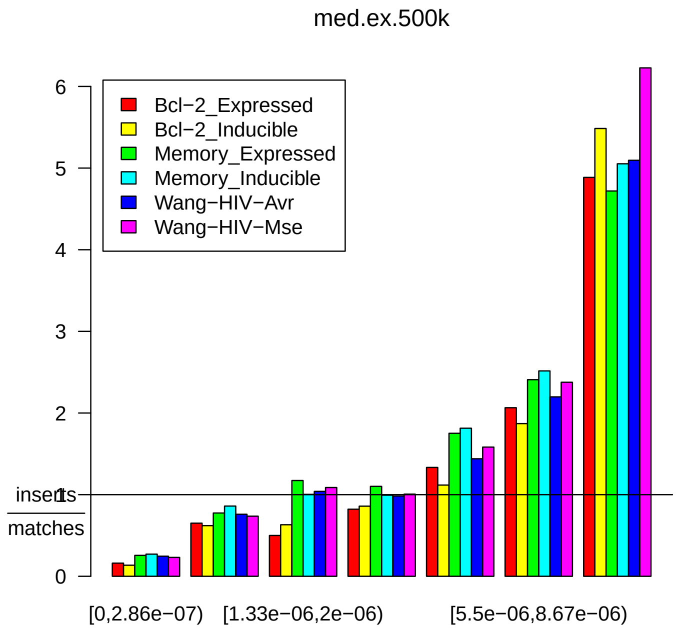

	coef	se	z	p
Bcl-2_Expressed	2.01	0.1960	10.3	9.87e-25
Bcl-2_Inducible	1.93	0.1520	12.7	4.16e-37
Memory_Expressed	1.72	0.0711	24.1	1.82e-128
Memory_Inducible	1.62	0.0513	31.5	4.81e-218
Wang-HIV-Avr	1.69	0.0192	88.1	0.00e+00
Wang-HIV-Mse	1.82	0.0185	98.1	0.00e+00

And here we count genes in the upper $1/16^{th}$:

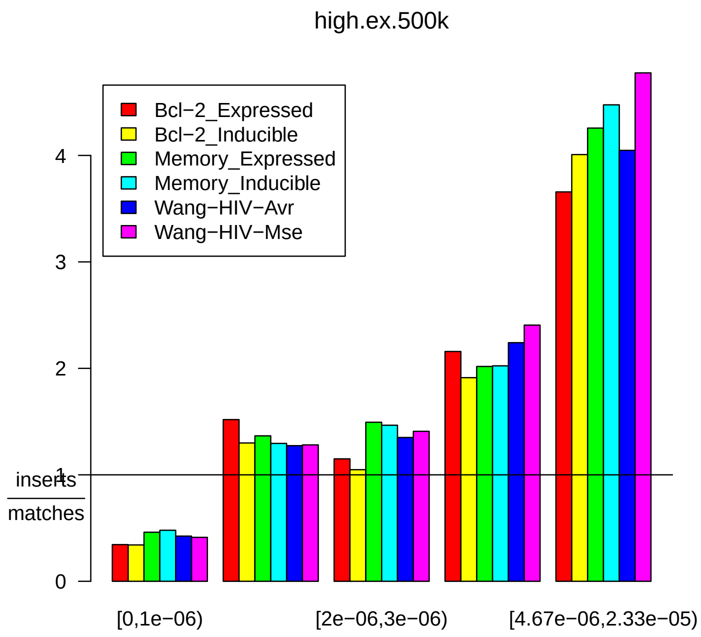

	coef	se	z	p
Bcl-2_Expressed	1.91	0.1820	10.5	1.16e-25
Bcl-2_Inducible	1.94	0.1520	12.7	3.72e-37
Memory_Expressed	1.58	0.0685	23.0	2.67e-117
Memory_Inducible	1.54	0.0502	30.7	1.15e-206
Wang-HIV-Avr	1.67	0.0189	88.2	0.00e+00
Wang-HIV-Mse	1.77	0.0181	97.5	0.00e+00

Here the effect of density of CpG islands is studied:

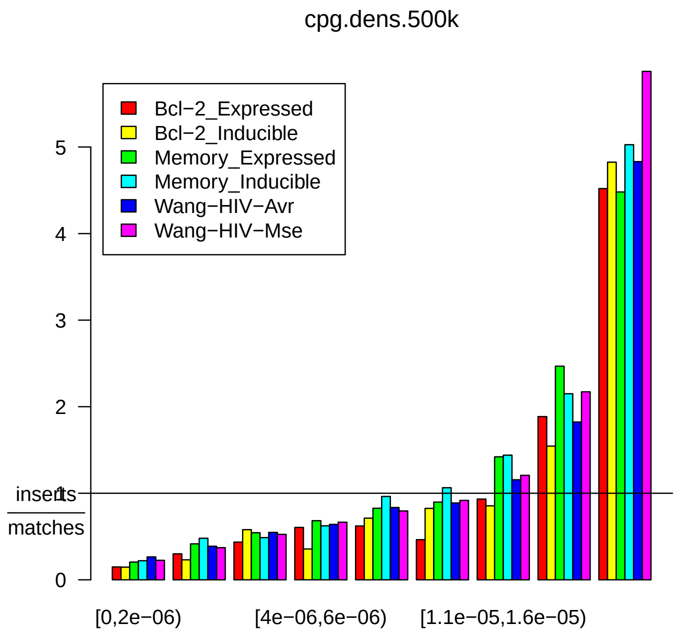

	coef	se	z	p
Bcl-2_Expressed	1.36	0.1640	8.34	7.50e-17
Bcl-2_Inducible	1.68	0.1420	11.80	2.91e-32
Memory_Expressed	1.39	0.0653	21.20	5.20e-100
Memory_Inducible	1.45	0.0484	29.90	6.98e-197
Wang-HIV-Avr	1.30	0.0174	74.80	0.00e+00
Wang-HIV-Mse	1.44	0.0167	86.30	0.00e+00

4.6 1 megabase Window

In the barplot that follows we examine the association of insertion sites with expression density in a 1 megabase window surrounding each locus. First, we count just the number of genes represented on the chip.

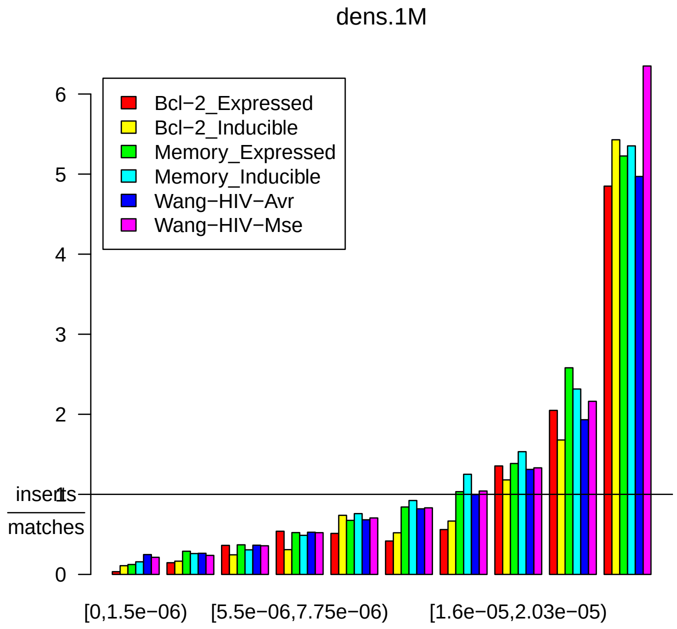

	coef	se	z	p
Bcl-2_Expressed	1.52	0.1700	8.93	4.29e-19
Bcl-2_Inducible	1.62	0.1380	11.70	1.01e-31
Memory_Expressed	1.59	0.0691	23.00	7.95e-117
Memory_Inducible	1.60	0.0507	31.50	2.62e-218
Wang-HIV-Avr	1.42	0.0179	79.30	0.00e+00
Wang-HIV-Mse	1.54	0.0172	89.30	0.00e+00

Here are the results for expression density. First, we count just genes that are in the upper half.

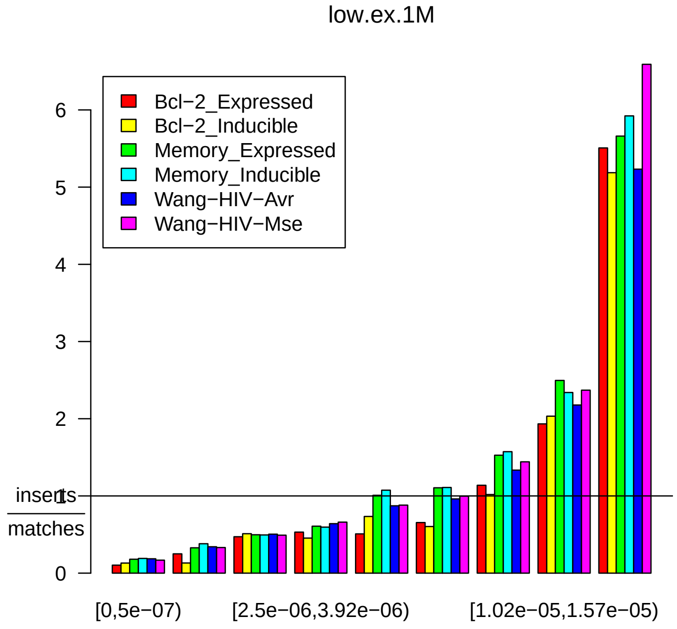

	coef	se	z	p
Bcl-2_Expressed	1.74	0.1830	9.48	2.47e-21
Bcl-2_Inducible	1.87	0.1520	12.30	7.43e-35
Memory_Expressed	1.73	0.0718	24.10	3.31e-128
Memory_Inducible	1.68	0.0523	32.20	8.05e-227
Wang-HIV-Avr	1.58	0.0188	84.30	0.00e+00
Wang-HIV-Mse	1.69	0.0180	93.90	0.00e+00

Now we count genes in the upper $1/8^{th}$:

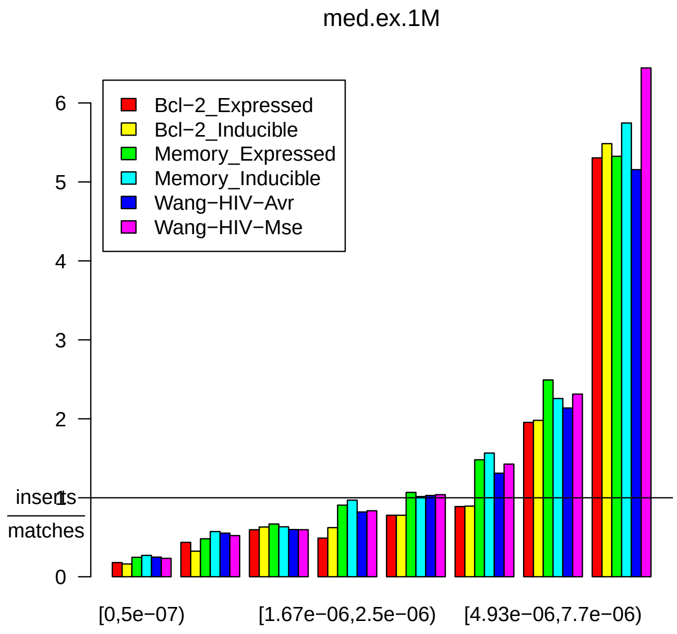

	coef	se	z	p
Bcl-2_Expressed	1.61	0.1710	9.39	5.89e-21
Bcl-2_Inducible	1.67	0.1400	11.90	1.58e-32
Memory_Expressed	1.59	0.0689	23.10	3.64e-118
Memory_Inducible	1.54	0.0502	30.60	7.45e-206
Wang-HIV-Avr	1.54	0.0185	83.10	0.00e+00
Wang-HIV-Mse	1.65	0.0178	92.80	0.00e+00

And here we count genes in the upper $1/16^{th}$:

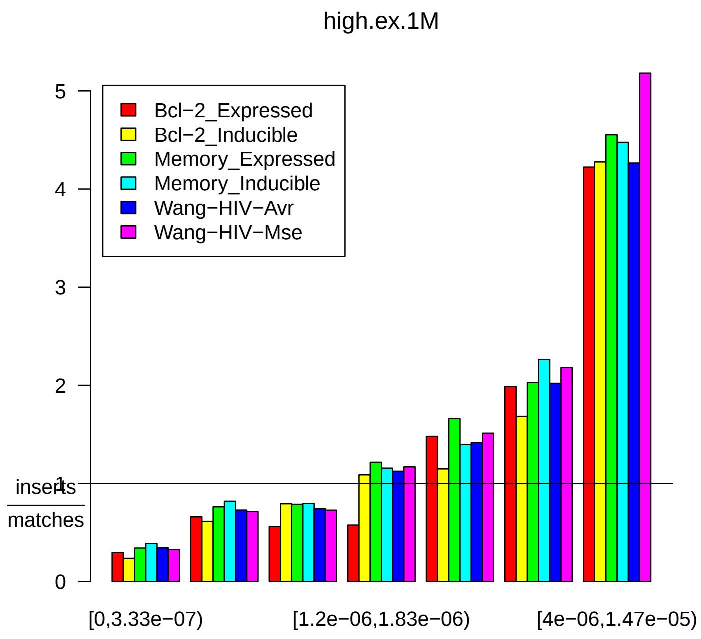

	coef	se	z	p
Bcl-2_Expressed	1.50	0.1670	9.01	2.13e-19
Bcl-2_Inducible	1.74	0.1430	12.10	9.20e-34
Memory_Expressed	1.45	0.0670	21.70	4.24e-104
Memory_Inducible	1.30	0.0480	27.10	4.22e-162
Wang-HIV-Avr	1.41	0.0180	78.20	0.00e+00
Wang-HIV-Mse	1.51	0.0173	87.60	0.00e+00

Here the effect of density of CpG islands is studied:

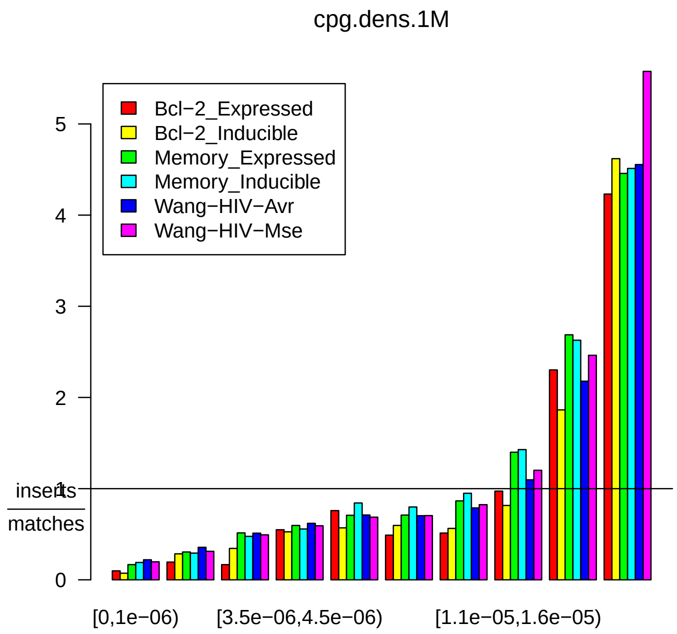

	coef	se	z	p
Bcl-2_Expressed	1.34	0.1640	8.16	3.23e-16
Bcl-2_Inducible	1.42	0.1350	10.50	7.78e-26
Memory_Expressed	1.30	0.0650	20.10	1.86e-89
Memory_Inducible	1.28	0.0475	26.90	6.73e-159
Wang-HIV-Avr	1.18	0.0171	68.70	0.00e+00
Wang-HIV-Mse	1.33	0.0165	80.60	0.00e+00

4.7 2 megabase Window

In the barplot that follows we examine the association of insertion sites with expression density in a 2 megabase window surrounding each locus. First, we count just the number of genes represented on the chip.

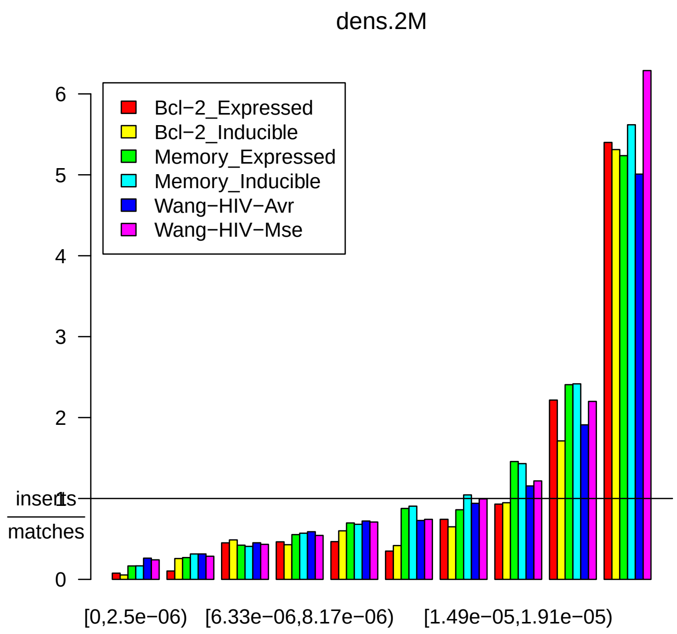

	coef	se	z	p
Bcl-2_Expressed	1.60	0.1750	9.19	4.10e-20
Bcl-2_Inducible	1.42	0.1310	10.90	1.94e-27
Memory_Expressed	1.43	0.0669	21.40	1.05e-101
Memory_Inducible	1.44	0.0494	29.20	1.59e-187
Wang-HIV-Avr	1.28	0.0176	72.80	0.00e+00
Wang-HIV-Mse	1.41	0.0169	83.90	0.00e+00

Here are the results for expression density. First, we count just genes that are in the upper half.

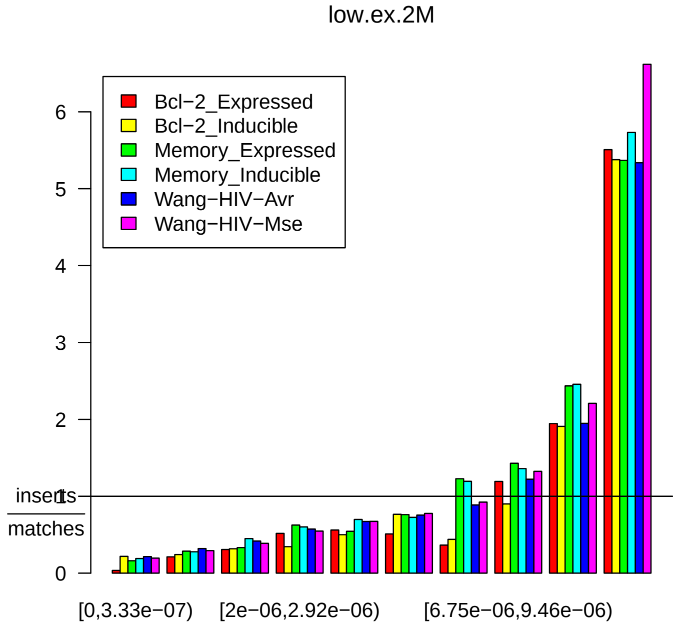

	coef	se	z	p
Bcl-2_Expressed	1.55	0.1740	8.9	5.57e-19
Bcl-2_Inducible	1.57	0.1370	11.5	1.81e-30
Memory_Expressed	1.56	0.0690	22.6	4.73e-113
Memory_Inducible	1.42	0.0491	28.9	9.14e-184
Wang-HIV-Avr	1.35	0.0178	75.8	0.00e+00
Wang-HIV-Mse	1.48	0.0171	86.6	0.00e+00

Now we count genes in the upper $1/8^{th}$:

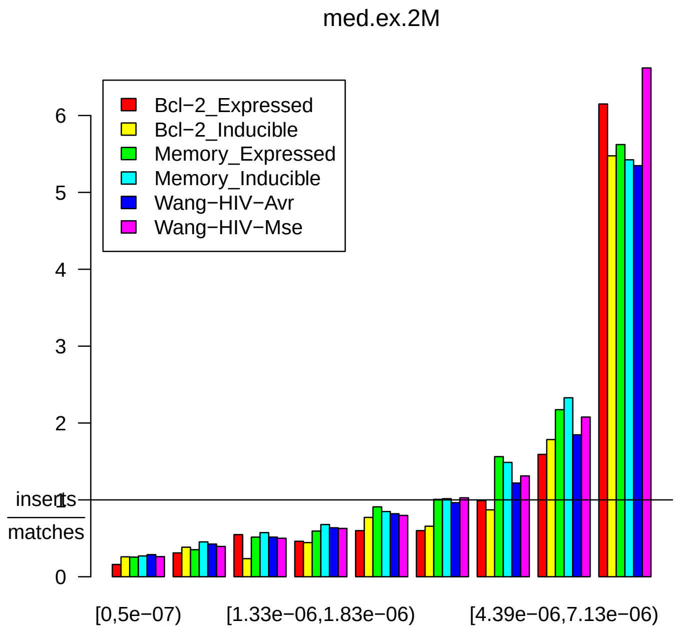

	coef	se	z	p
Bcl-2_Expressed	1.57	0.1710	9.15	5.48e-20
Bcl-2_Inducible	1.68	0.1410	11.90	8.98e-33
Memory_Expressed	1.56	0.0688	22.60	2.42e-113
Memory_Inducible	1.39	0.0487	28.60	4.40e-180
Wang-HIV-Avr	1.38	0.0180	77.00	0.00e+00
Wang-HIV-Mse	1.51	0.0172	87.70	0.00e+00

And here we count genes in the upper $1/16^{th}$:

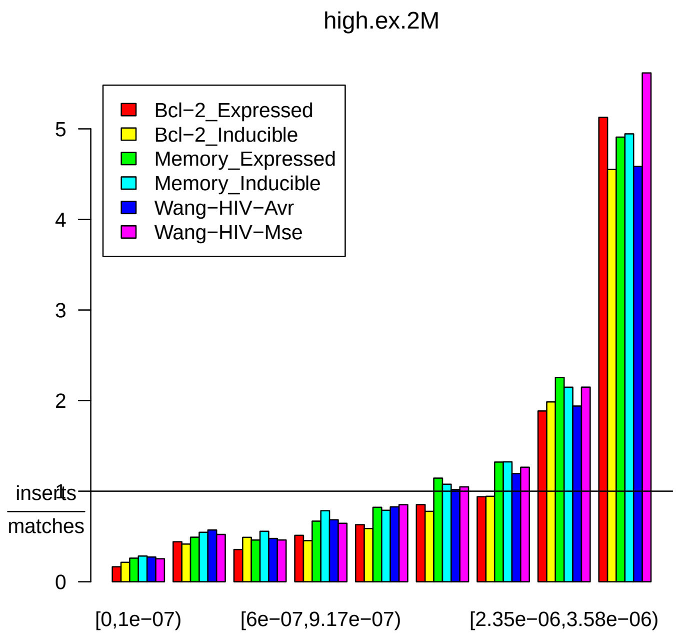

	coef	se	z	p
Bcl-2_Expressed	1.68	0.1820	9.26	2.07e-20
Bcl-2_Inducible	1.52	0.1360	11.10	7.71e-29
Memory_Expressed	1.43	0.0667	21.40	5.04e-102
Memory_Inducible	1.26	0.0474	26.70	1.01e-156
Wang-HIV-Avr	1.31	0.0177	74.30	0.00e+00
Wang-HIV-Mse	1.45	0.0170	85.30	0.00e+00

Here the effect of density of CpG islands is studied:

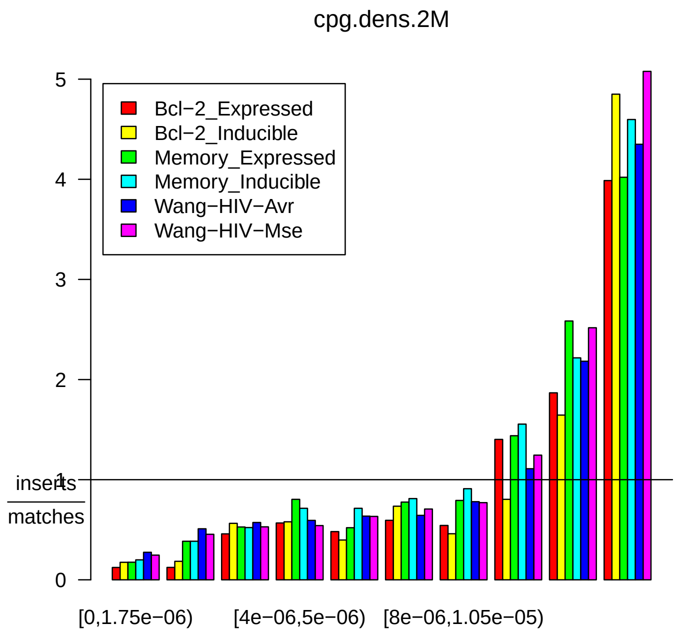

	coef	se	z	p
Bcl-2_Expressed	1.48	0.1690	8.74	2.30e-18
Bcl-2_Inducible	1.34	0.1300	10.30	6.62e-25
Memory_Expressed	1.24	0.0644	19.20	4.21e-82
Memory_Inducible	1.21	0.0467	26.00	2.25e-149
Wang-HIV-Avr	1.11	0.0170	65.60	0.00e+00
Wang-HIV-Mse	1.28	0.0164	78.20	0.00e+00

4.8 4 megabase Window

In the barplot that follows we examine the association of insertion sites with expression density in a 4 megabase window surrounding each locus. First, we count just the number of genes represented on the chip.

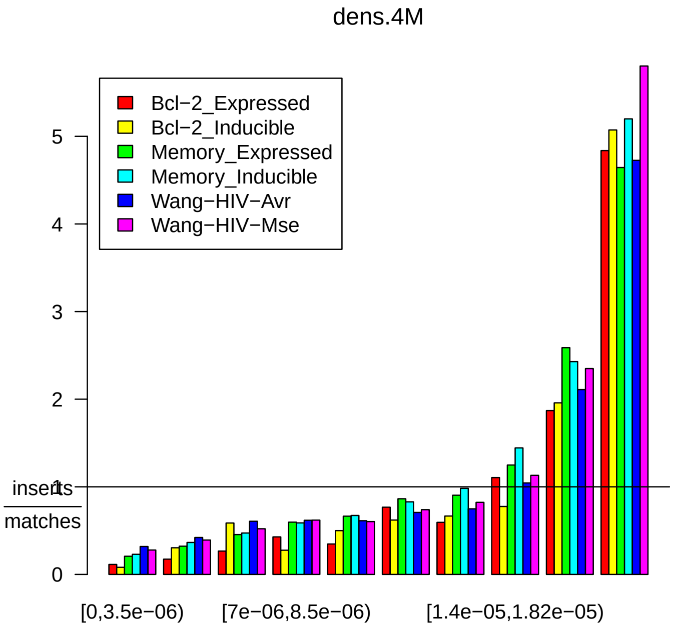

	coef	se	z	p
Bcl-2_Expressed	1.79	0.1840	9.72	2.49e-22
Bcl-2_Inducible	1.49	0.1370	10.90	8.65e-28
Memory_Expressed	1.35	0.0662	20.30	5.76e-92
Memory_Inducible	1.32	0.0479	27.60	8.39e-168
Wang-HIV-Avr	1.13	0.0171	65.80	0.00e+00
Wang-HIV-Mse	1.28	0.0165	77.40	0.00e+00

Here are the results for expression density. First, we count just genes that are in the upper half.

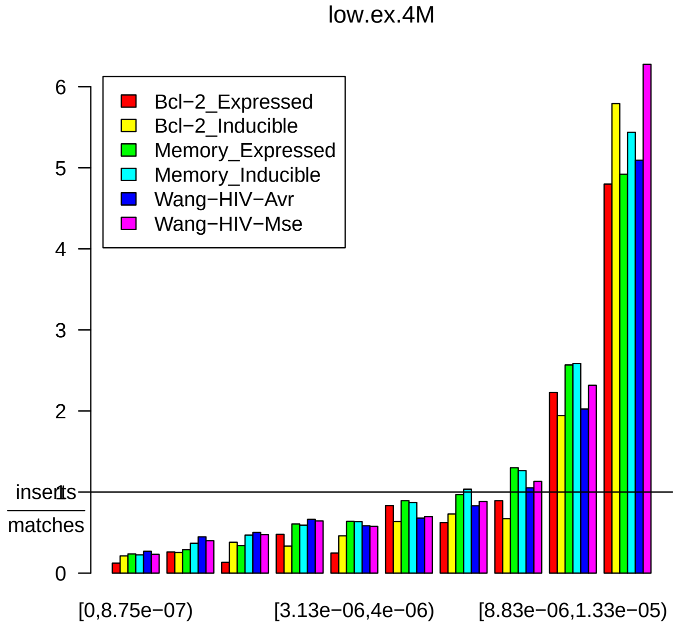

	coef	se	z	p
Bcl-2_Expressed	1.86	0.1880	9.86	6.39e-23
Bcl-2_Inducible	1.57	0.1380	11.30	7.98e-30
Memory_Expressed	1.46	0.0681	21.40	1.77e-101
Memory_Inducible	1.34	0.0481	27.80	4.05e-170
Wang-HIV-Avr	1.19	0.0173	68.70	0.00e+00
Wang-HIV-Mse	1.33	0.0167	79.90	0.00e+00

Now we count genes in the upper $1/8^{th}$:

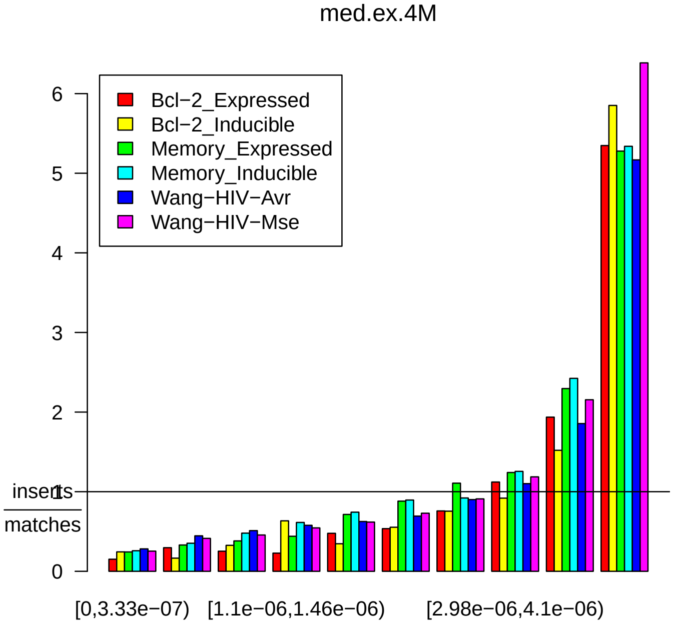

	coef	se	z	p
Bcl-2_Expressed	1.70	0.1790	9.45	3.32e-21
Bcl-2_Inducible	1.53	0.1370	11.10	7.85e-29
Memory_Expressed	1.45	0.0679	21.30	1.04e-100
Memory_Inducible	1.25	0.0472	26.50	8.29e-155
Wang-HIV-Avr	1.20	0.0173	69.20	0.00e+00
Wang-HIV-Mse	1.35	0.0167	80.70	0.00e+00

And here we count genes in the upper $1/16^{th}$:

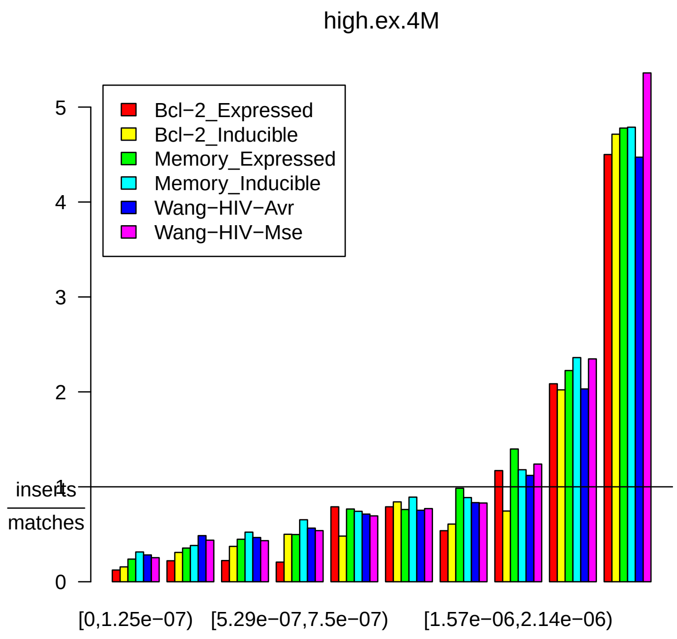

	coef	se	z	p
Bcl-2_Expressed	1.62	0.1760	9.23	2.66e-20
Bcl-2_Inducible	1.44	0.1340	10.80	3.56e-27
Memory_Expressed	1.31	0.0661	19.90	6.60e-88
Memory_Inducible	1.15	0.0464	24.80	1.66e-135
Wang-HIV-Avr	1.16	0.0172	67.30	0.00e+00
Wang-HIV-Mse	1.29	0.0165	78.20	0.00e+00

Here the effect of density of CpG islands is studied:

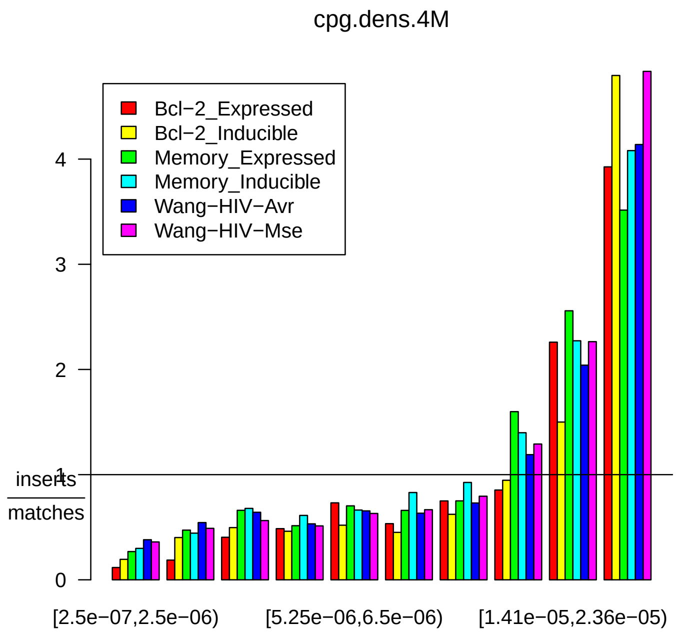

	coef	se	z	p
Bcl-2_Expressed	1.36	0.1680	8.11	5.13e-16
Bcl-2_Inducible	1.31	0.1290	10.10	5.31e-24
Memory_Expressed	1.11	0.0635	17.50	9.45e-69
Memory_Inducible	1.10	0.0459	24.00	8.78e-128
Wang-HIV-Avr	1.03	0.0168	61.30	0.00e+00
Wang-HIV-Mse	1.18	0.0162	73.10	0.00e+00

4.9 8 megabase Window

In the barplot that follows we examine the association of insertion sites with expression density in a 8 megabase window surrounding each locus. First, we count just the number of genes represented on the chip.

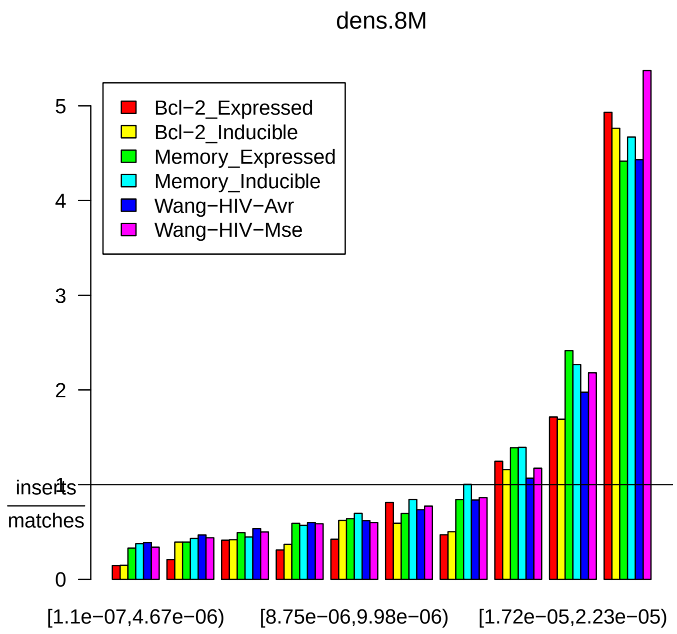

	coef	se	z	p
Bcl-2_Expressed	1.71	0.1850	9.24	2.49e-20
Bcl-2_Inducible	1.33	0.1310	10.20	2.90e-24
Memory_Expressed	1.19	0.0641	18.60	7.51e-77
Memory_Inducible	1.20	0.0470	25.50	9.15e-144
Wang-HIV-Avr	1.11	0.0171	64.80	0.00e+00
Wang-HIV-Mse	1.23	0.0163	75.40	0.00e+00

Here are the results for expression density. First, we count just genes that are in the upper half.

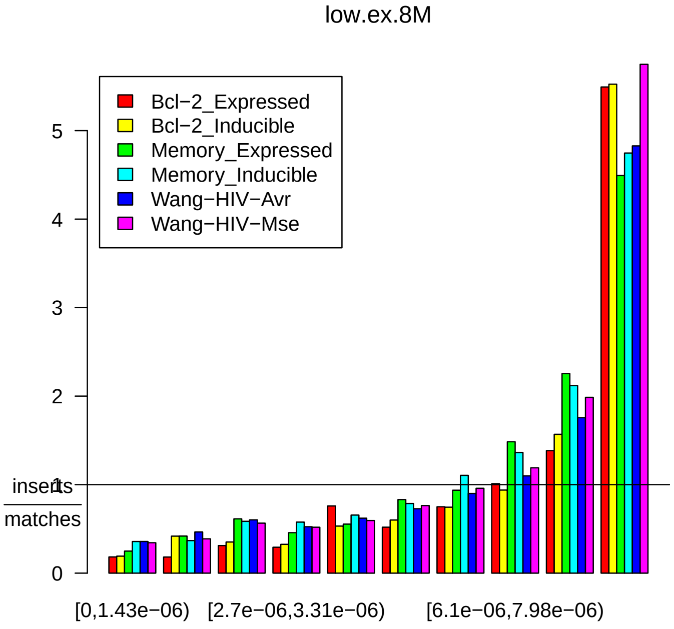

	coef	se	z	p
Bcl-2_Expressed	1.54	0.1770	8.68	4.03e-18
Bcl-2_Inducible	1.44	0.1340	10.70	9.40e-27
Memory_Expressed	1.30	0.0652	19.90	5.16e-88
Memory_Inducible	1.20	0.0473	25.40	4.78e-142
Wang-HIV-Avr	1.13	0.0171	65.90	0.00e+00
Wang-HIV-Mse	1.27	0.0165	77.20	0.00e+00

Now we count genes in the upper $1/8^{th}$:

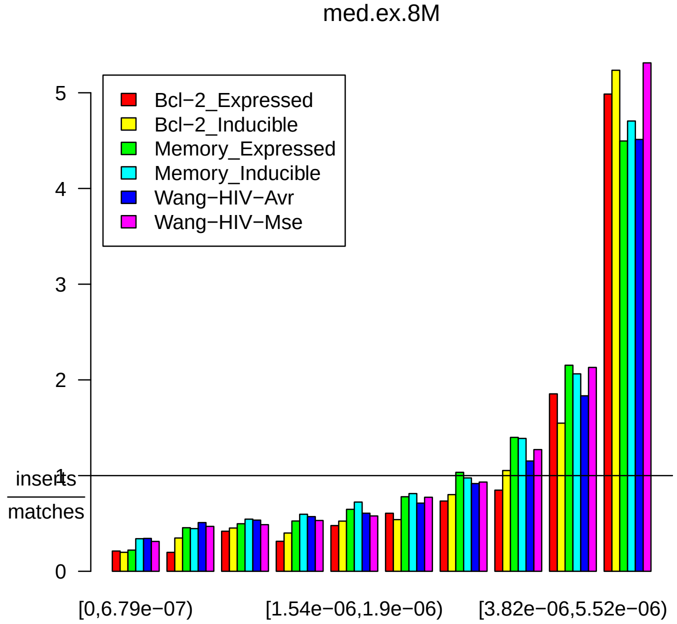

	coef	se	z	p
Bcl-2_Expressed	1.61	0.1770	9.07	1.23e-19
Bcl-2_Inducible	1.37	0.1300	10.60	2.96e-26
Memory_Expressed	1.28	0.0651	19.70	3.91e-86
Memory_Inducible	1.13	0.0463	24.30	1.63e-130
Wang-HIV-Avr	1.13	0.0171	66.00	0.00e+00
Wang-HIV-Mse	1.29	0.0165	77.90	0.00e+00

And here we count genes in the upper $1/16^{th}$:

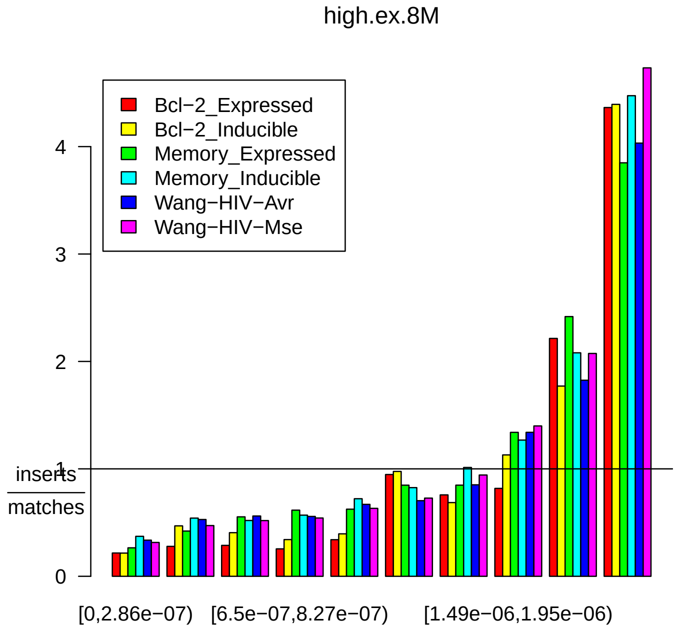

	coef	se	z	p
Bcl-2_Expressed	1.78	0.1890	9.45	3.38e-21
Bcl-2_Inducible	1.44	0.1280	11.20	3.81e-29
Memory_Expressed	1.19	0.0640	18.50	1.18e-76
Memory_Inducible	1.08	0.0458	23.60	2.53e-123
Wang-HIV-Avr	1.08	0.0169	63.80	0.00e+00
Wang-HIV-Mse	1.23	0.0163	75.10	0.00e+00

Here the effect of density of CpG islands is studied:

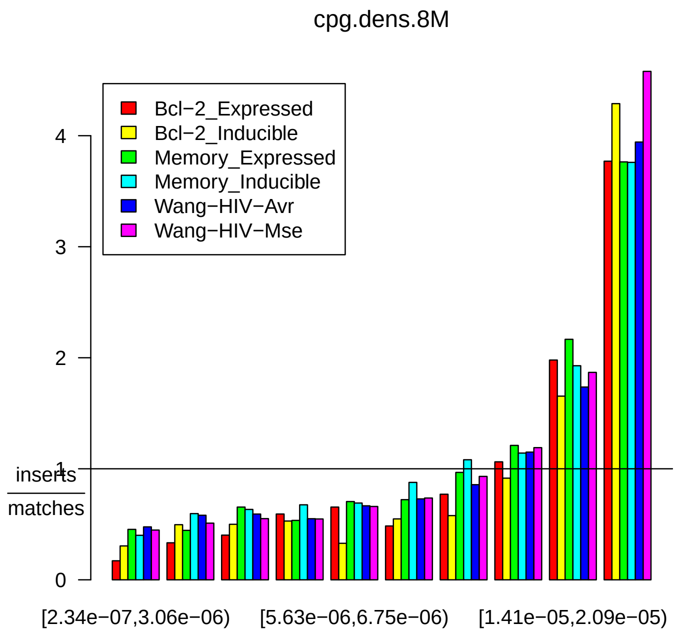

	coef	se	z	p
Bcl-2_Expressed	1.220	0.1630	7.47	8.02e-14
Bcl-2_Inducible	1.290	0.1310	9.83	8.54e-23
Memory_Expressed	1.020	0.0625	16.30	5.30e-60
Memory_Inducible	0.922	0.0445	20.70	2.49e-95
Wang-HIV-Avr	0.955	0.0166	57.40	0.00e+00
Wang-HIV-Mse	1.070	0.0159	67.30	0.00e+00

4.10 16 megabase Window

In the barplot that follows we examine the association of insertion sites with expression density in a 16 megabase window surrounding each locus. First, we count just the number of genes represented on the chip.

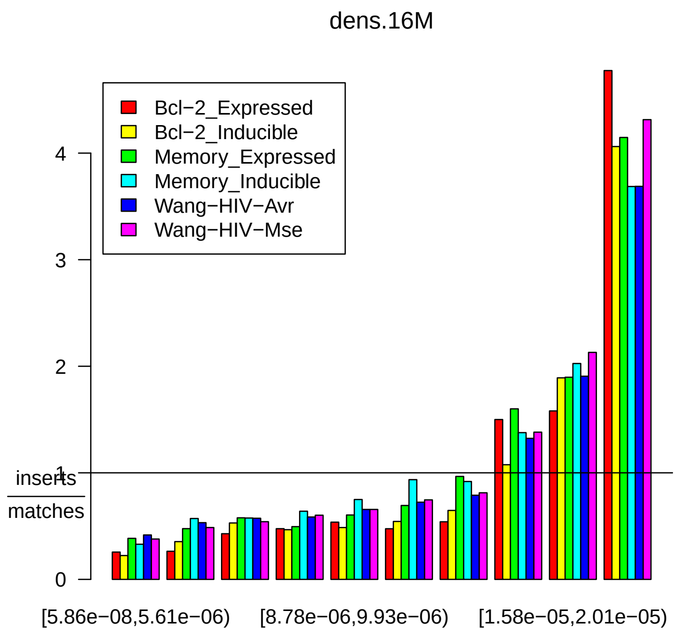

	coef	se	z	p
Bcl-2_Expressed	1.38	0.1690	8.2	2.46e-16
Bcl-2_Inducible	1.32	0.1290	10.2	1.69e-24
Memory_Expressed	1.17	0.0644	18.1	1.38e-73
Memory_Inducible	1.01	0.0454	22.3	2.14e-110
Wang-HIV-Avr	1.02	0.0168	60.7	0.00e+00
Wang-HIV-Mse	1.11	0.0160	69.2	0.00e+00

Here are the results for expression density. First, we count just genes that are in the upper half.

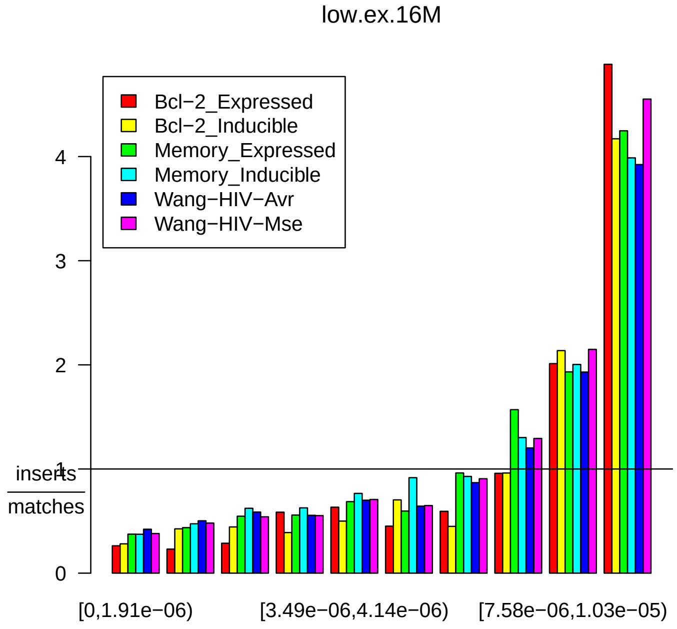

	coef	se	z	p
Bcl-2_Expressed	1.34	0.1650	8.1	5.50e-16
Bcl-2_Inducible	1.33	0.1280	10.3	5.45e-25
Memory_Expressed	1.12	0.0636	17.6	1.09e-69
Memory_Inducible	1.01	0.0451	22.3	1.45e-110
Wang-HIV-Avr	1.02	0.0168	60.8	0.00e+00
Wang-HIV-Mse	1.11	0.0160	69.5	0.00e+00

Now we count genes in the upper $1/8^{th}$:

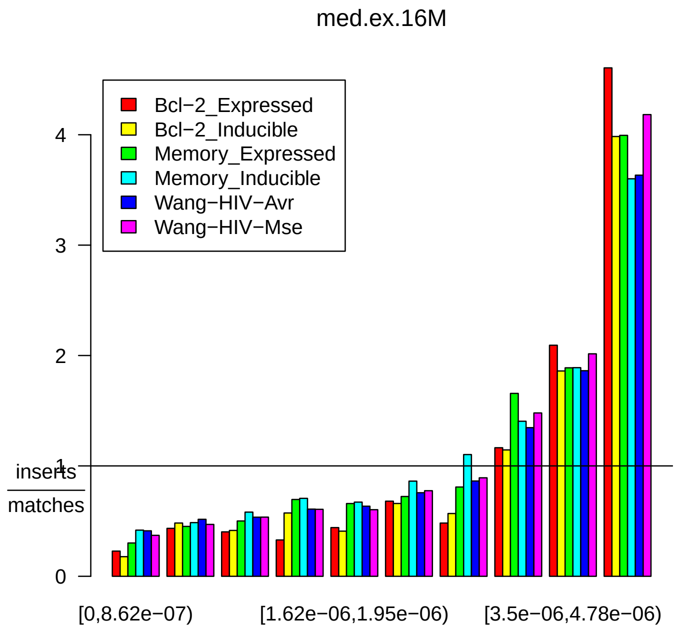

	coef	se	z	p
Bcl-2_Expressed	1.47	0.1700	8.61	7.15e-18
Bcl-2_Inducible	1.33	0.1290	10.30	6.86e-25
Memory_Expressed	1.12	0.0633	17.70	6.26e-70
Memory_Inducible	1.00	0.0452	22.20	2.98e-109
Wang-HIV-Avr	1.05	0.0169	62.30	0.00e+00
Wang-HIV-Mse	1.15	0.0162	71.40	0.00e+00

And here we count genes in the upper $1/16^{th}$:

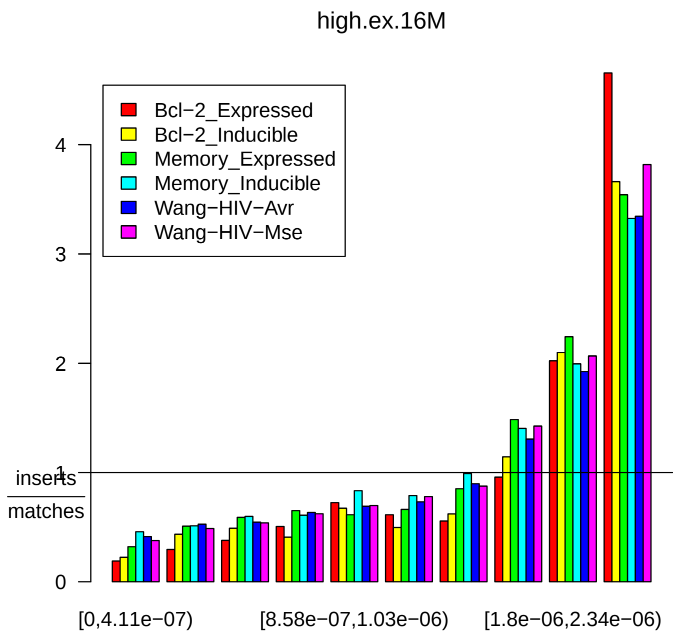

	coef	se	z	p
Bcl-2_Expressed	1.310	0.1620	8.04	8.82e-16
Bcl-2_Inducible	1.180	0.1230	9.58	9.76e-22
Memory_Expressed	1.060	0.0629	16.90	4.37e-64
Memory_Inducible	0.935	0.0448	20.90	1.02e-96
Wang-HIV-Avr	0.994	0.0167	59.50	0.00e+00
Wang-HIV-Mse	1.080	0.0160	67.50	0.00e+00

Here the effect of density of CpG islands is studied:

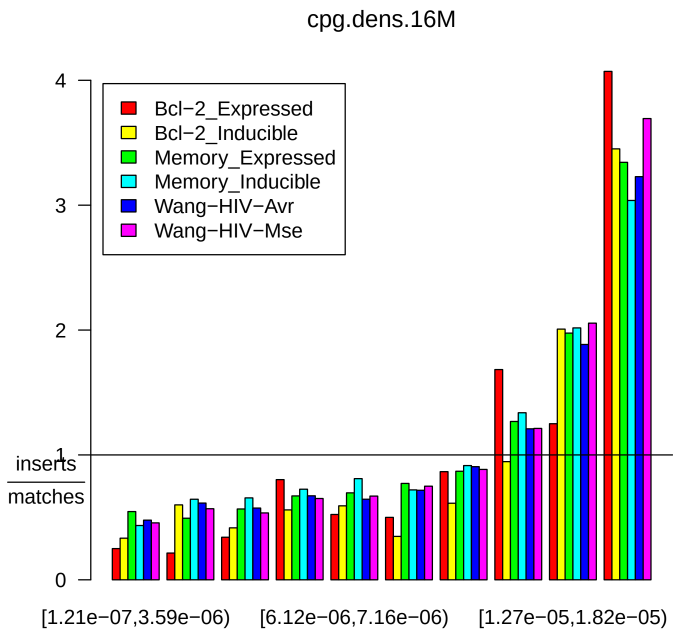

	coef	se	z	p
Bcl-2_Expressed	1.240	0.1590	7.81	5.74e-15
Bcl-2_Inducible	1.070	0.1210	8.81	1.22e-18
Memory_Expressed	0.951	0.0627	15.20	5.40e-52
Memory_Inducible	0.796	0.0440	18.10	4.94e-73
Wang-HIV-Avr	0.905	0.0165	55.00	0.00e+00
Wang-HIV-Mse	0.977	0.0157	62.20	0.00e+00

4.11 32 megabase Window

In the barplot that follows we examine the association of insertion sites with expression density in a 32 megabase window surrounding each locus. First, we count just the number of genes represented on the chip.

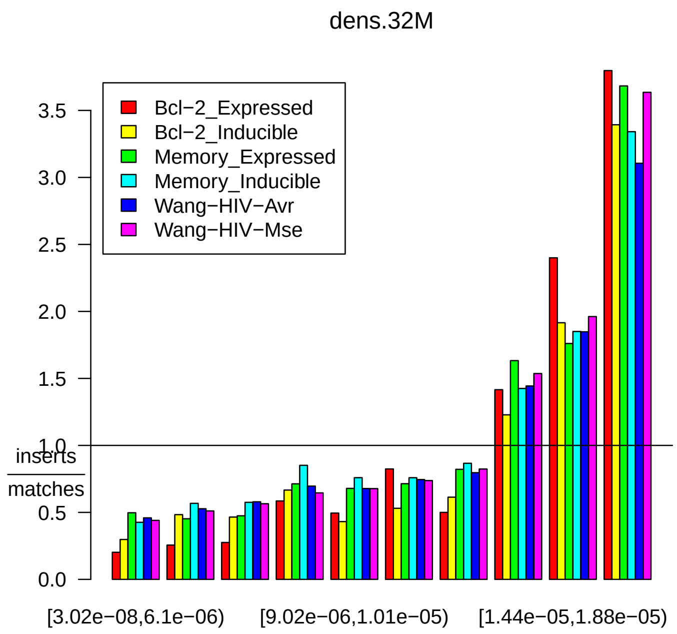

	coef	se	z	p
Bcl-2_Expressed	1.580	0.1760	9.01	2.10e-19
Bcl-2_Inducible	1.200	0.1230	9.72	2.41e-22
Memory_Expressed	1.000	0.0621	16.20	1.11e-58
Memory_Inducible	0.837	0.0443	18.90	1.51e-79
Wang-HIV-Avr	0.926	0.0165	56.00	0.00e+00
Wang-HIV-Mse	1.010	0.0158	63.70	0.00e+00

Here are the results for expression density. First, we count just genes that are in the upper half.

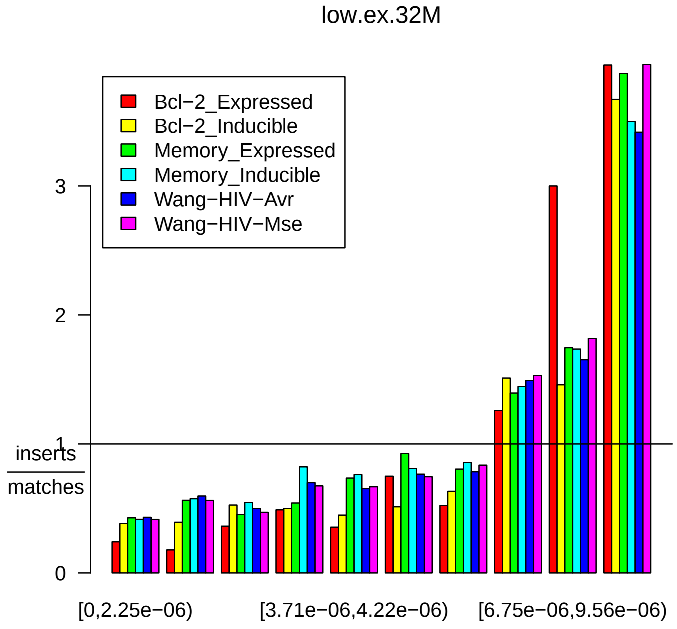

	coef	se	z	p
Bcl-2_Expressed	1.660	0.1780	9.3	1.46e-20
Bcl-2_Inducible	1.280	0.1260	10.2	1.50e-24
Memory_Expressed	1.060	0.0632	16.7	1.18e-62
Memory_Inducible	0.863	0.0443	19.5	2.09e-84
Wang-HIV-Avr	0.957	0.0166	57.6	0.00e+00
Wang-HIV-Mse	1.040	0.0159	65.5	0.00e+00

Now we count genes in the upper $1/8^{th}$:

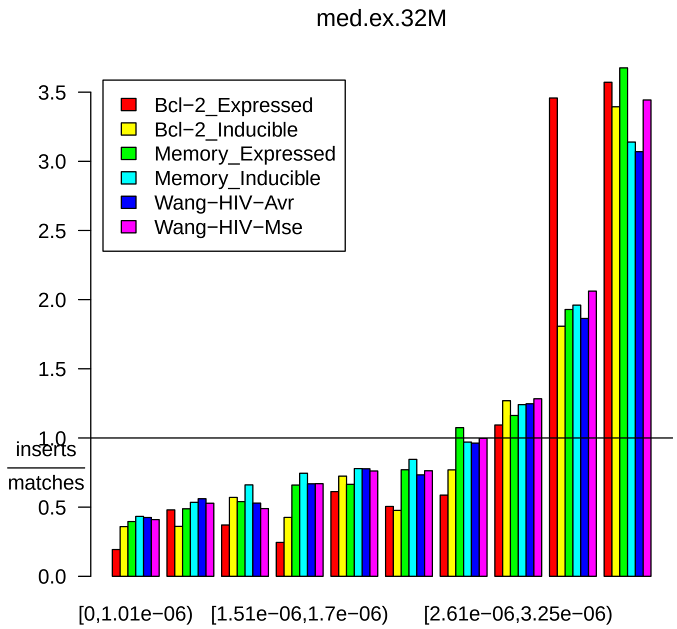

	coef	se	z	p
Bcl-2_Expressed	1.500	0.1690	8.88	6.45e-19
Bcl-2_Inducible	1.160	0.1220	9.46	2.96e-21
Memory_Expressed	1.040	0.0628	16.50	2.67e-61
Memory_Inducible	0.847	0.0443	19.10	1.46e-81
Wang-HIV-Avr	0.919	0.0166	55.50	0.00e+00
Wang-HIV-Mse	1.000	0.0158	63.50	0.00e+00

And here we count genes in the upper $1/16^{th}$:

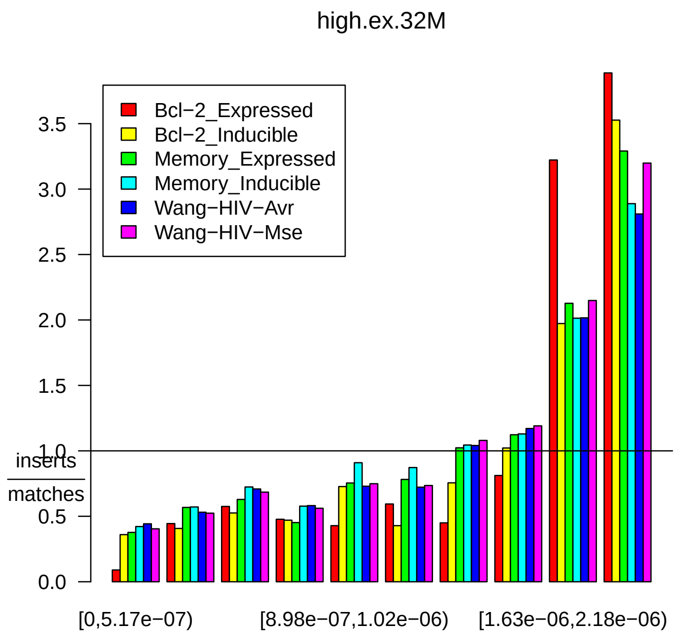

	coef	se	z	p
Bcl-2_Expressed	1.490	0.1720	8.65	5.08e-18
Bcl-2_Inducible	1.100	0.1200	9.23	2.62e-20
Memory_Expressed	1.020	0.0628	16.20	4.94e-59
Memory_Inducible	0.825	0.0443	18.60	1.77e-77
Wang-HIV-Avr	0.898	0.0165	54.50	0.00e+00
Wang-HIV-Mse	0.967	0.0157	61.60	0.00e+00

Here the effect of density of CpG islands is studied:

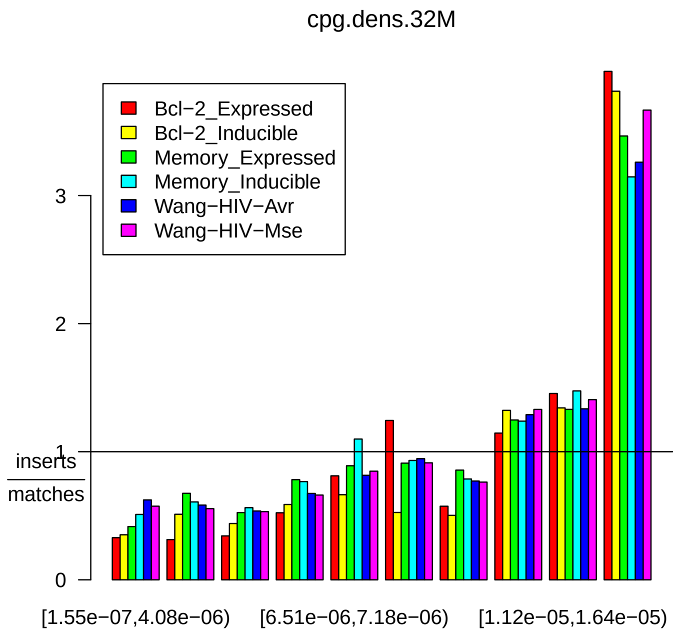

	coef	se	z	p
Bcl-2_Expressed	1.210	0.1580	7.65	1.94e-14
Bcl-2_Inducible	1.040	0.1190	8.77	1.72e-18
Memory_Expressed	0.779	0.0607	12.80	1.05e-37
Memory_Inducible	0.665	0.0438	15.20	3.76e-52
Wang-HIV-Avr	0.779	0.0162	48.10	0.00e+00
Wang-HIV-Mse	0.828	0.0155	53.60	0.00e+00

5 Juxtaposition with Gene Start and End Positions

5.1 Acembly Annotations

In this section we study the effect of juxtaposition in terms of gene start and end positions. The first barplot shows the effect of gene width for those insertions that are located within an Acembly gene. The table following the barplot shows the p-values for a test of the hypothesis that the proportions in each of the categories that define the bars are equal in the insertions and their matches. This p-value is obtained from the $5 \times 2 \times k$ table of counts defined by gene width category, insertion/match status, and stratum (consisting of an insertion and its matched sites) using a likelihood ratio test for the hypothesis of no association between gene width category and insertion/match status. The test used compared the log-linear model [1] with all two-way configurations to that with no gene width category and insertion/match status configuration.

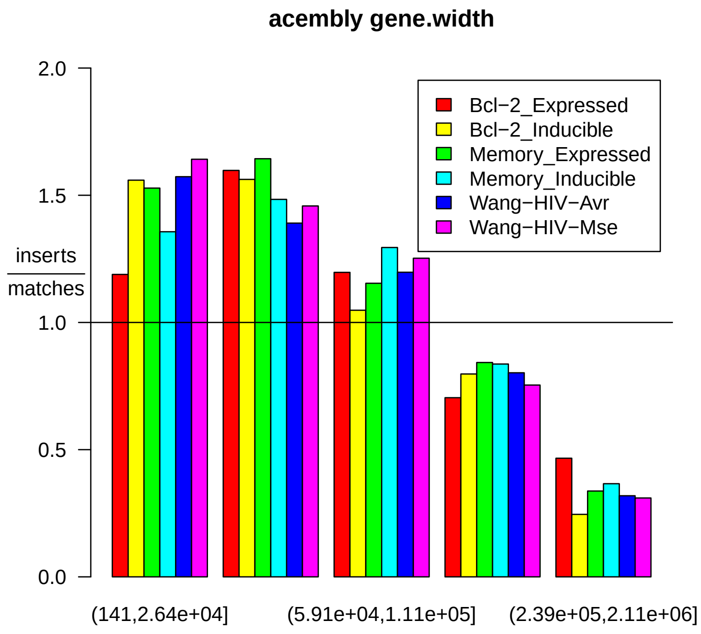

Bcl-2_Expressed	Bcl-2_Inducible	Memory_Expressed	Memory_Inducible
3.32e-07	3.71e-24	3.57e-58	4.98e-94
Wang-HIV-Avr	Wang-HIV-Mse		
0.00e+00	0.00e+00		

The next plot uses the width of a non-gene region for insertions that fall into such regions.

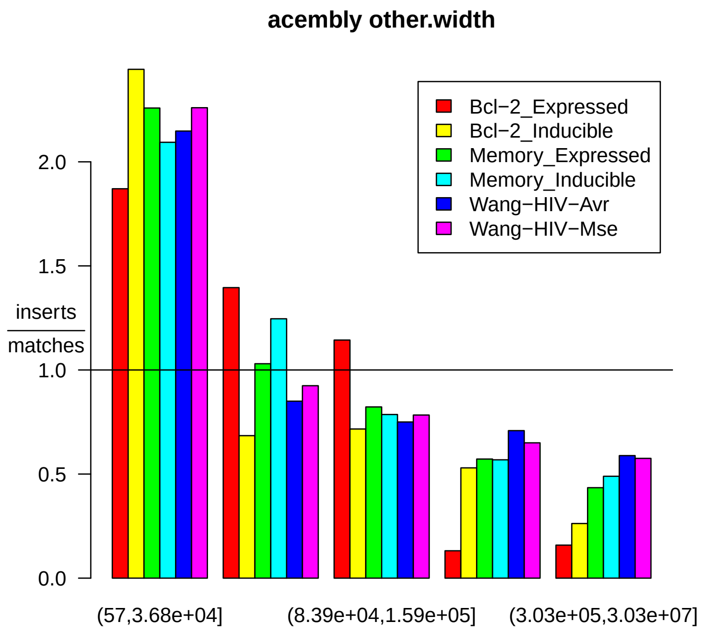


```
Bcl-2_Expressed  Bcl-2_Inducible Memory_Expressed Memory_Inducible
       8.67e-08         6.94e-08         5.08e-18         1.97e-26
    Wang-HIV-Avr     Wang-HIV-Mse
      3.15e-144        5.64e-160
```

The next plot studies the distance to the nearest boundary between a gene and a non-gene region. The distance is expressed as a fraction of the length of the region. Thus, '0.25' refers to one quarter of the distance from the site to nearest boundary divided by the total width of the region.

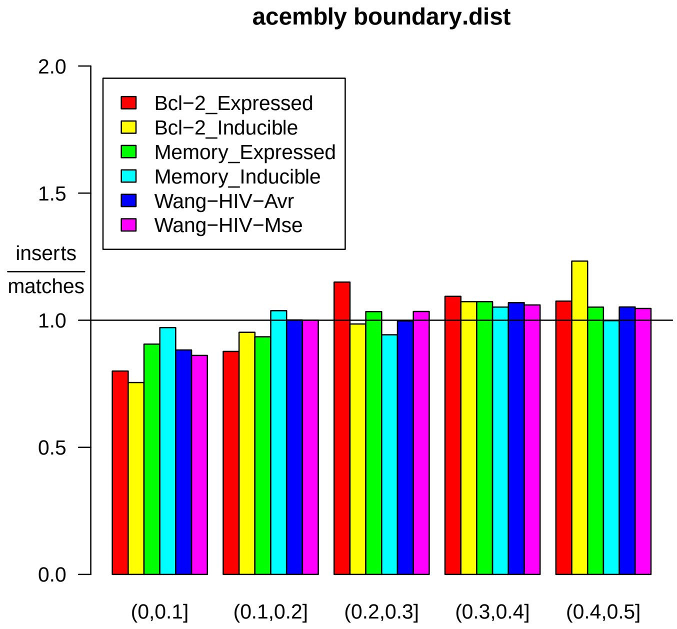


```
Bcl-2_Expressed  Bcl-2_Inducible Memory_Expressed Memory_Inducible
       2.33e-01         1.94e-02         1.38e-01         2.93e-01
   Wang-HIV-Avr     Wang-HIV-Mse
       9.40e-19         2.42e-26
```

This plot studies the effect of nearness to the beginning of a transcript. For sites in genes, it is the distance to the start of the gene divided by the width of the gene. For other sites it is the distance from the site to the nearer gene if that gene boundary is also a transcription starting point. Locations near '0' are relatively near the beginning of transcription, while those near '1' are near the termination of the transcript.

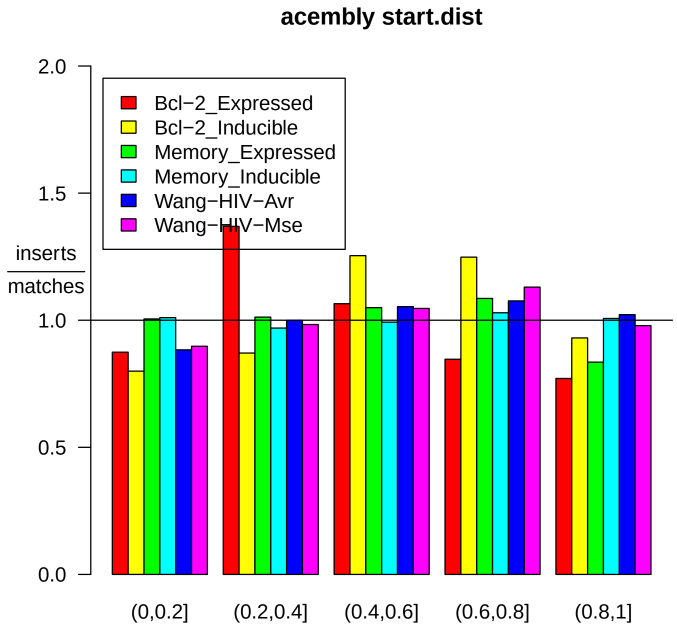


```
Bcl-2_Expressed  Bcl-2_Inducible Memory_Expressed Memory_Inducible
       9.99e-03         1.25e-03         2.51e-02         9.33e-01
   Wang-HIV-Avr     Wang-HIV-Mse
       1.69e-20         1.79e-27
```

5.2 RefSeq Annotations

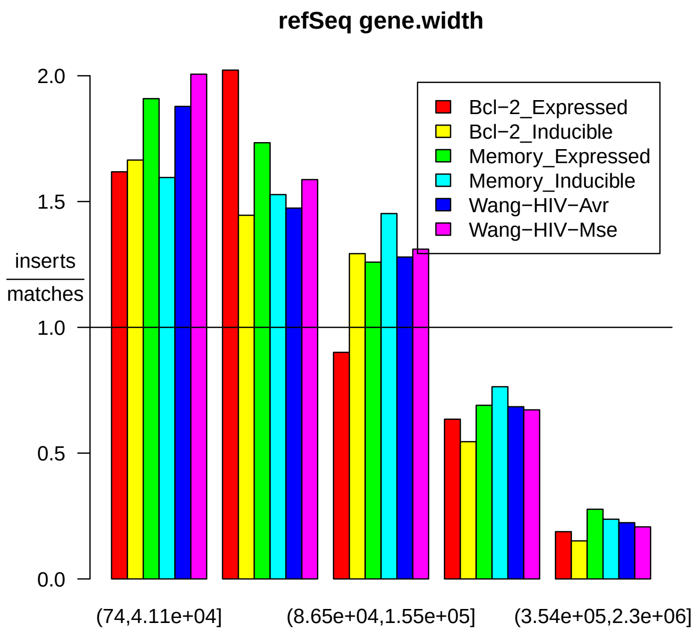

Bcl-2_Expressed	Bcl-2_Inducible	Memory_Expressed	Memory_Inducible
3.75e-14	2.06e-26	2.01e-79	7.35e-131

Wang-HIV-Avr	Wang-HIV-Mse
0.00e+00	0.00e+00

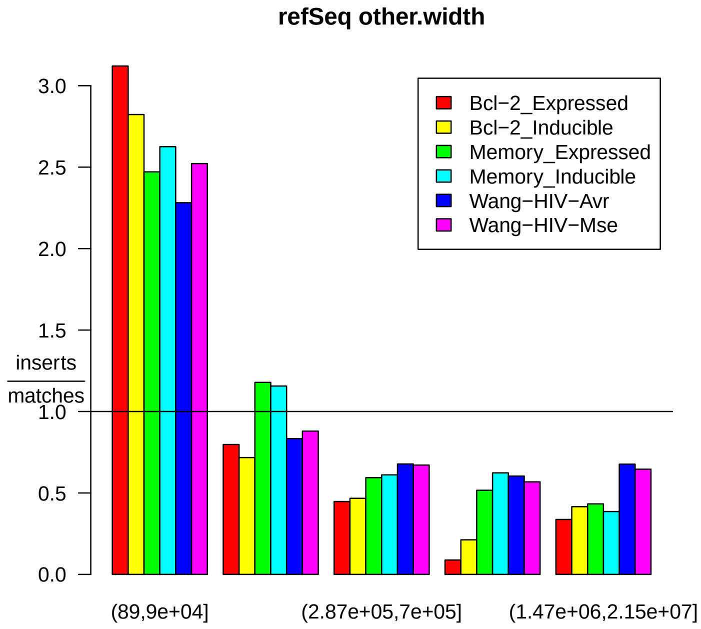


```
Bcl-2_Expressed  Bcl-2_Inducible Memory_Expressed Memory_Inducible
       4.07e-11         6.83e-14         4.43e-33         8.96e-72
   Wang-HIV-Avr     Wang-HIV-Mse
      6.46e-269         0.00e+00
```

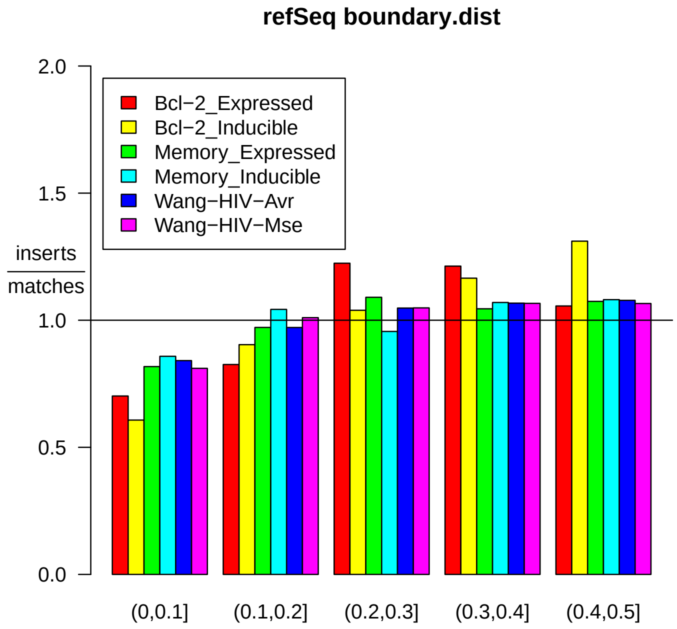


```
Bcl-2_Expressed  Bcl-2_Inducible Memory_Expressed Memory_Inducible
       8.06e-03         5.11e-06         2.13e-03         2.33e-04
   Wang-HIV-Avr     Wang-HIV-Mse
       3.85e-35         7.33e-49
```

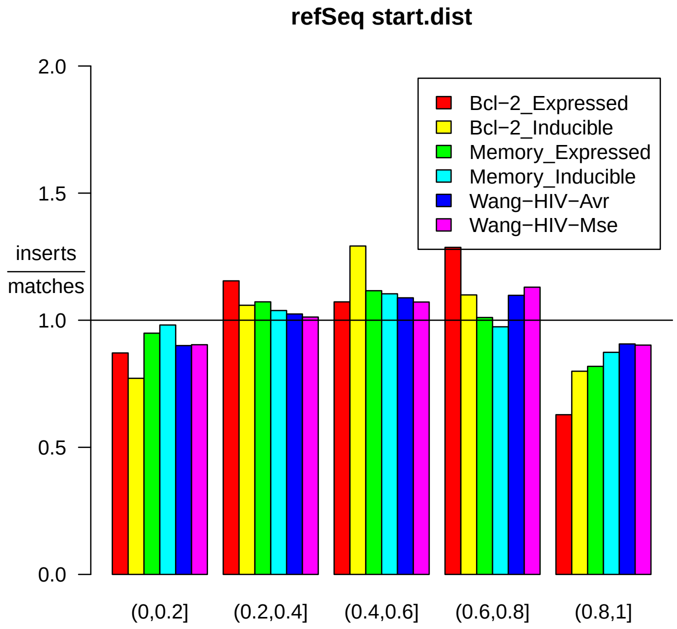


```
Bcl-2_Expressed  Bcl-2_Inducible Memory_Expressed Memory_Inducible
       5.78e-03         3.35e-03         2.71e-02         2.72e-03
   Wang-HIV-Avr     Wang-HIV-Mse
       3.35e-30         3.12e-35
```

5.3 genScan Annotations

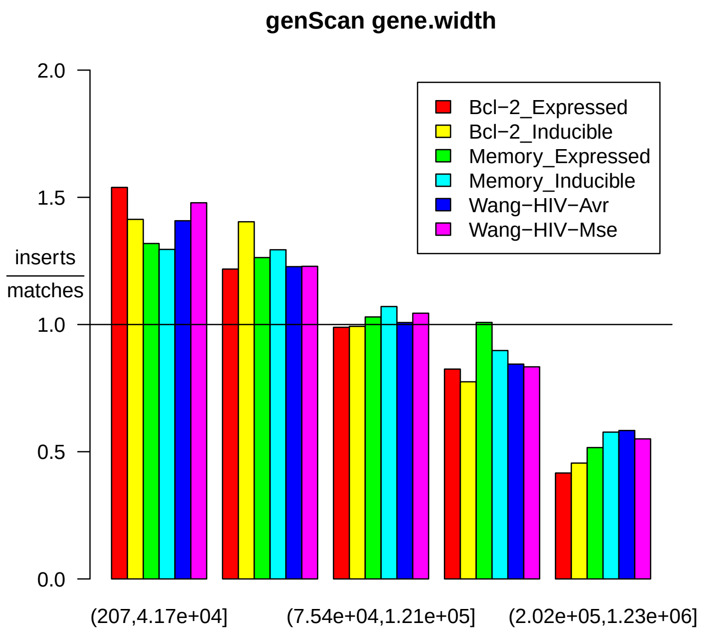


```
Bcl-2_Expressed  Bcl-2_Inducible Memory_Expressed Memory_Inducible
       1.81e-06         8.03e-10         2.00e-24         2.50e-38
   Wang-HIV-Avr     Wang-HIV-Mse
      4.13e-313         0.00e+00
```

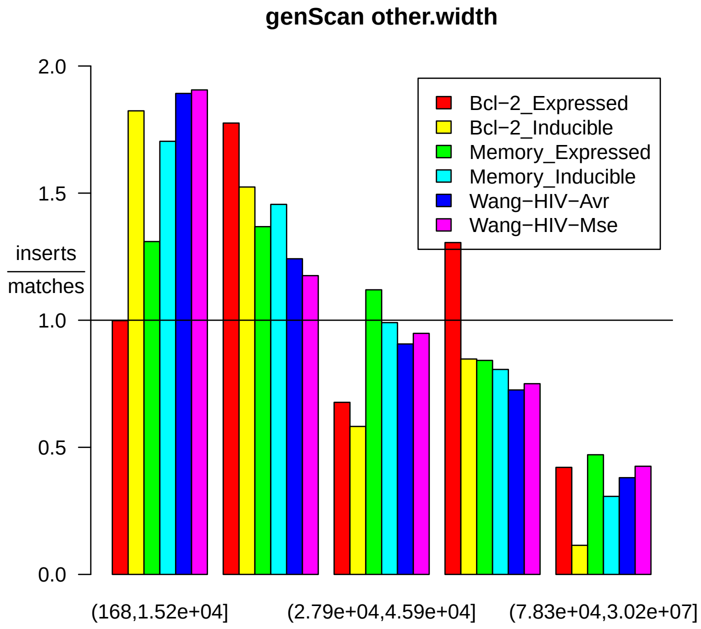

Bcl-2_Expressed	Bcl-2_Inducible	Memory_Expressed	Memory_Inducible
4.07e-02	2.72e-08	1.27e-04	2.00e-24
Wang-HIV-Avr	Wang-HIV-Mse		
1.38e-192	1.52e-170		

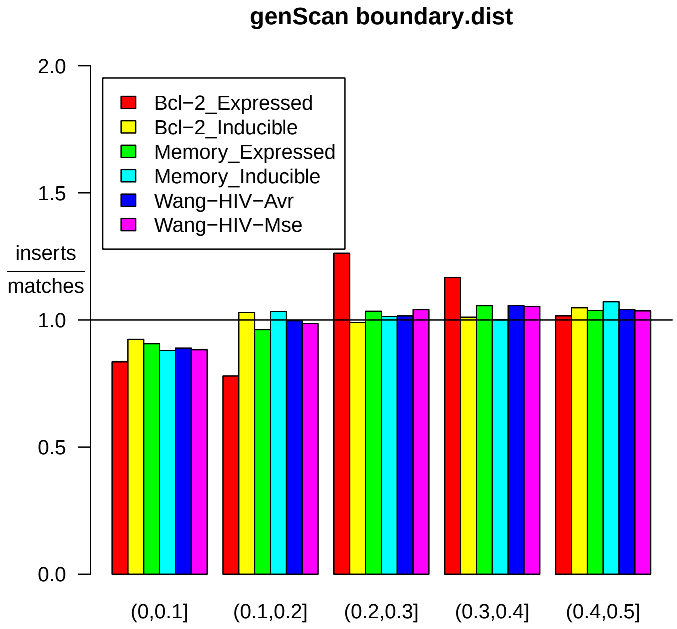


```
Bcl-2_Expressed  Bcl-2_Inducible Memory_Expressed Memory_Inducible
       3.68e-02         9.34e-01         2.73e-01         1.53e-02
   Wang-HIV-Avr     Wang-HIV-Mse
       2.46e-15         3.12e-19
```

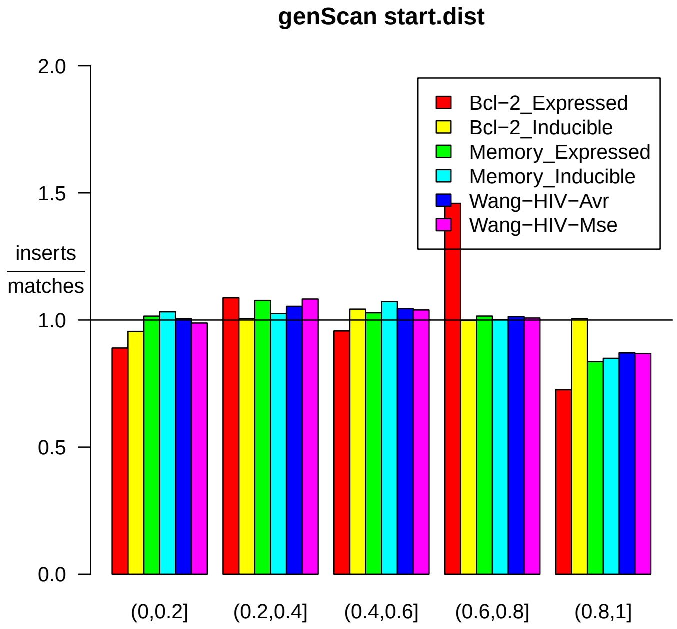


```
Bcl-2_Expressed  Bcl-2_Inducible Memory_Expressed Memory_Inducible
       4.54e-03         9.21e-01         4.57e-02         1.16e-03
   Wang-HIV-Avr     Wang-HIV-Mse
       4.77e-18         5.00e-22
```

5.4 uniGene Annotations

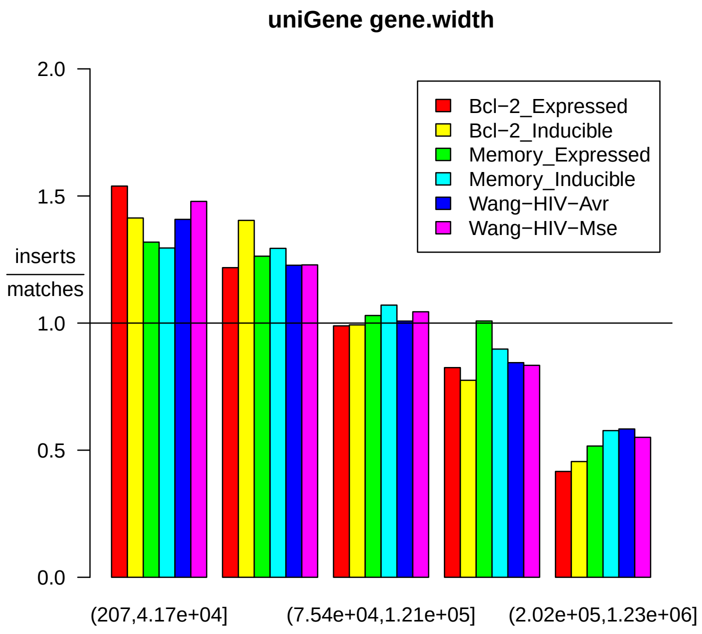

Bcl-2_Expressed	Bcl-2_Inducible	Memory_Expressed	Memory_Inducible
1.81e-06	8.03e-10	2.00e-24	2.50e-38
Wang-HIV-Avr	Wang-HIV-Mse		
4.13e-313	0.00e+00		

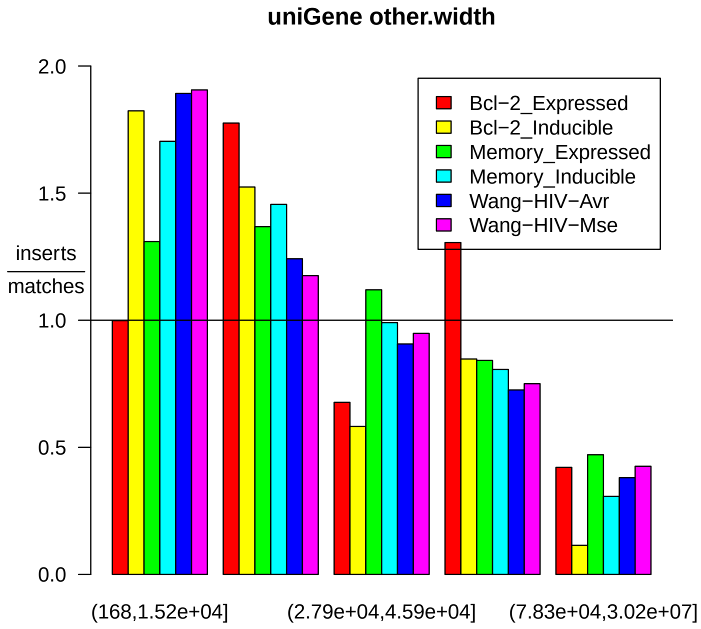

Bcl-2_Expressed	Bcl-2_Inducible	Memory_Expressed	Memory_Inducible
4.07e-02	2.72e-08	1.27e-04	2.00e-24
Wang-HIV-Avr	Wang-HIV-Mse		
1.38e-192	1.52e-170		

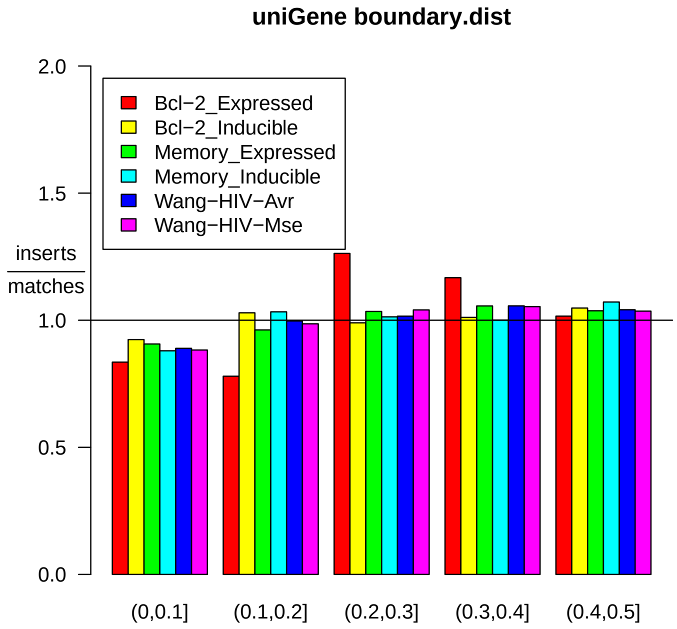

Bcl-2_Expressed	Bcl-2_Inducible	Memory_Expressed	Memory_Inducible
3.68e-02	9.34e-01	2.73e-01	1.53e-02
Wang-HIV-Avr	Wang-HIV-Mse		
2.46e-15	3.12e-19		

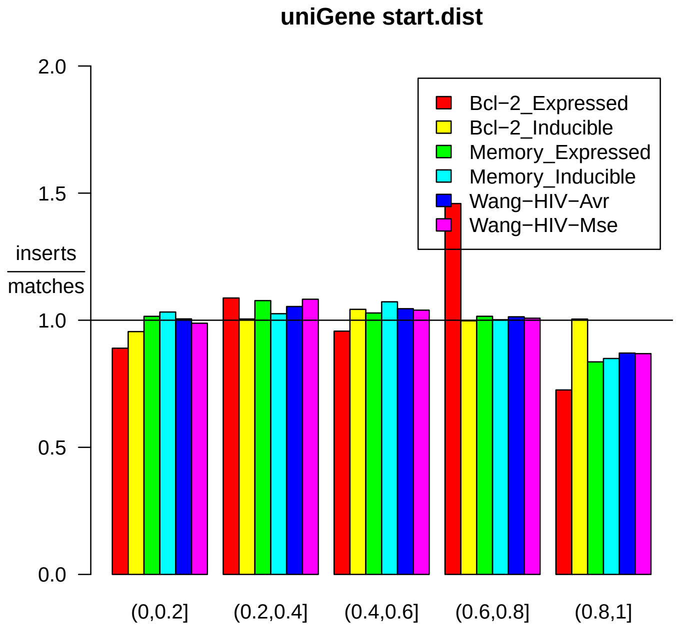

Bcl-2_Expressed	Bcl-2_Inducible	Memory_Expressed	Memory_Inducible
4.54e-03	9.21e-01	4.57e-02	1.16e-03
Wang-HIV-Avr	Wang-HIV-Mse		
4.77e-18	5.00e-22		

6 GC content

Here we study the effect of GC content on insertion. The GC content is taken from the Human Genome Draft at GoldenPath from the table `http://genome.ucsc.edu/goldenPath/hg18/database/gc5Base.txt.gz`.

Following the plot is a table of fitted coefficients based on splitting the GC percent data at the median.

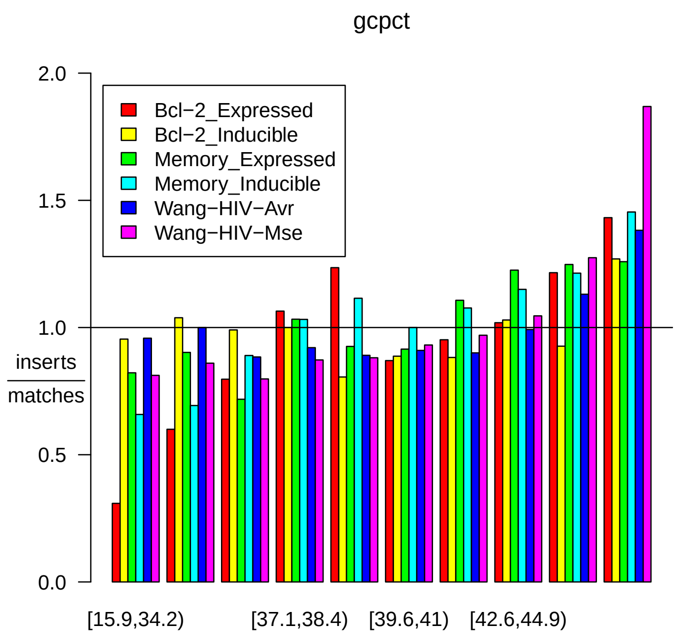

	coef	se	z	p
Bcl-2_Expressed	0.3480	0.1460	2.390	1.70e-02
Bcl-2_Inducible	0.0456	0.1100	0.413	6.80e-01
Memory_Expressed	0.2700	0.0589	4.590	4.43e-06
Memory_Inducible	0.2920	0.0430	6.790	1.11e-11
Wang-HIV-Avr	0.1390	0.0155	8.940	3.87e-19
Wang-HIV-Mse	0.3410	0.0149	22.900	6.39e-116

7 Cytobands

Here we study the association of cytoband with insertion intensity. The data are obtained from
`http://genome.ucsc.edu/goldenPath/hg18/database/cytoBand.txt.gz`.

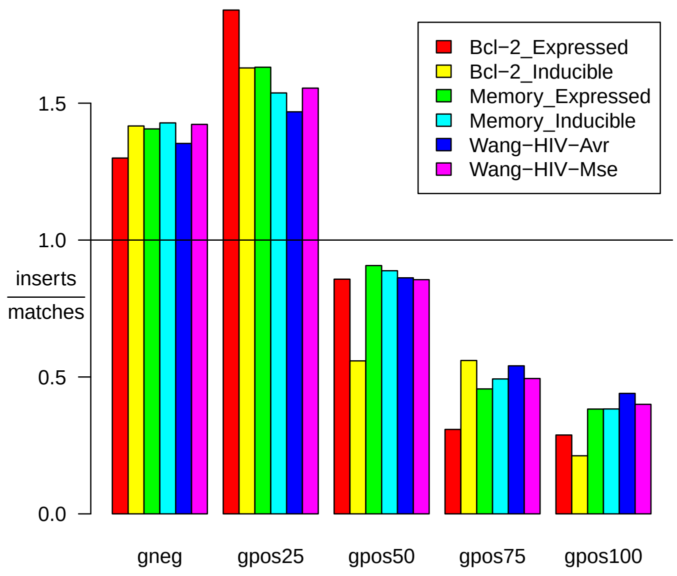

A formal test of significance attains a p-value of $< 2.22e - 16$. Here is the table of coefficients of the log ratio of intensities for true insertion sites versus control insertion sites (comparing each category of Giemsa staining to 'gneg') along with their standard errors, z statistics, and p-values:

```
                   coef     se       z         p
cyto.typegpos100 -1.2200 0.0186 -65.50  0.00e+00
cyto.typegpos25   0.0901 0.0178   5.07  3.92e-07
cyto.typegpos50  -0.4840 0.0156 -31.10 1.03e-211
cyto.typegpos75  -1.0000 0.0189 -53.00  0.00e+00
```

References

[1] Yvonne M.M. Bishop, Stephen E. Fienberg, and Paul W. Holland. *Discrete multivariate analyses: Theory and practice* (MIT Press, 1975).

[2] P. McCullagh and John A. Nelder. *Generalized linear models.* (Chapman & Hall ltd, 1999).

[3] Xiaolin Wu, Yuan Li, Bruce Crise, Shawn M. Burgess "Transcription Start Regions in the Human Genome Are Favored Targets for MLV Integration," *Science,* **300**(5626), (June 2003): 1749-1751.
